# High-Performance Thermoplastics from a Unique Bicyclic Lignin-Derived Diol

**DOI:** 10.1021/acssuschemeng.2c05998

**Published:** 2023-02-06

**Authors:** Xianyuan Wu, Mario De bruyn, Gregor Trimmel, Klaus Zangger, Katalin Barta

**Affiliations:** † Stratingh Institute for Chemistry, University of Groningen, Nijenborgh 4, 9747 AG Groningen, Groningen, The Netherlands; ‡ Department of Chemistry, Organic and Bioorganic Chemistry, University of Graz, Heinrichstrasse 28/II, 8010 Graz, Austria; § Institute for Chemistry and Technology of Materials (ICTM), NAWI Graz, Graz University of Technology, Stremayrgasse 9, 8010 Graz, Austria

**Keywords:** lignin-derived diol, fully bio-based polyesters, good thermal properties, good recyclability, high-energy-density jet fuels

## Abstract

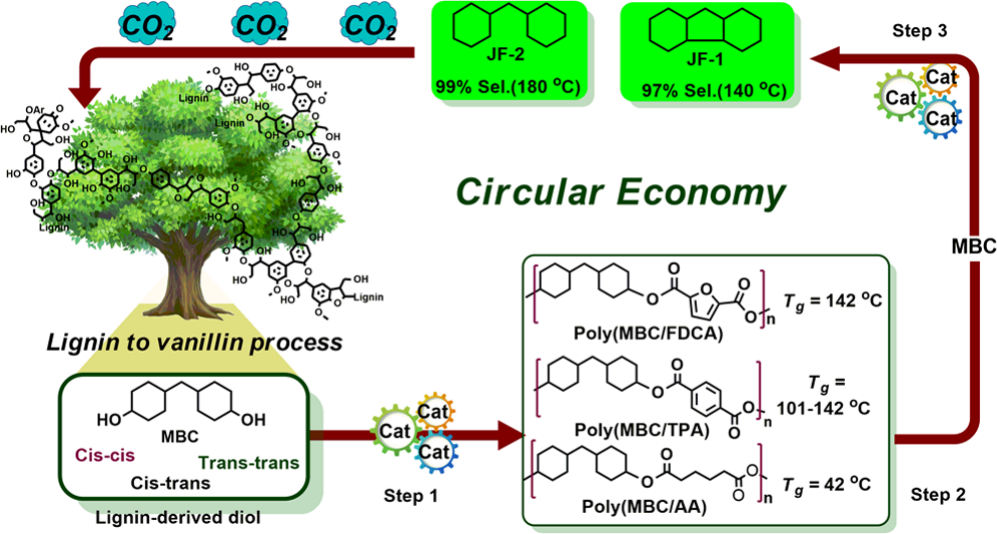

Polyesters are an important class of thermoplastic polymers, and there is a clear demand to find high-performing, recyclable, and renewable alternatives. In this contribution, we describe a range of fully bio-based polyesters obtained upon the polycondensation of the lignin-derived bicyclic diol 4,4′-methylenebiscyclohexanol (MBC) with various cellulose-derived diesters. Interestingly, the use of MBC in combination with either dimethyl terephthalate (DMTA) or dimethyl furan-2,5-dicarboxylate (DMFD) resulted in polymers with industrially relevant glass transition temperatures in the 103–142 °C range and high decomposition temperatures (261–365 °C range). Since MBC is obtained as a mixture of three distinct isomers, in-depth NMR-based structural characterization of the MBC isomers and thereof derived polymers is provided. Moreover, a practical method for the separation of all MBC isomers is presented. Interestingly, clear effects on the glass transition, melting, and decomposition temperatures, as well as polymer solubility, were evidenced with the use of isomerically pure MBC. Importantly, the polyesters can be efficiently depolymerized by methanolysis with an MBC diol recovery yield of up to 90%. The catalytic hydrodeoxygenation of the recovered MBC into two high-performance specific jet fuel additives was demonstrated as an attractive end-of-life option.

## Abstract 2

This work describes the synthesis of fully bio-based and high-performance polyesters, which can be further recycled into jet fuels.

## Introduction

Given their lightweightness and versatile properties, polyesters [e.g., poly(ethylene terephthalate) (PET)] have assumed an ever more dominating and beneficial role in our society for use in packaging materials, textiles, fibers, and single-use bottles, reaching an estimated annual production of up to 70 million tons.^1−3^ As the main downside, this has led to the accumulation of an estimated 530 million tons of polyester plastic waste in landfills and oceans.^1,4,5^ Therefore, there is an urgent need to implement circular economy approaches with regard to polymers through the development of fully bio-based and recyclable polyester plastics and appropriate upcycling or reconversion strategies.^6−13^ This requires the development of novel strategies that allow us to source novel bio-based monomers from abundantly available renewable starting materials.^14−17^ Moreover, the polymers produced from such virgin monomers should reach a similar or better performance in applications compared to their synthetic analogues, but at the same time, should be readily degradable.

**Figure 1:**
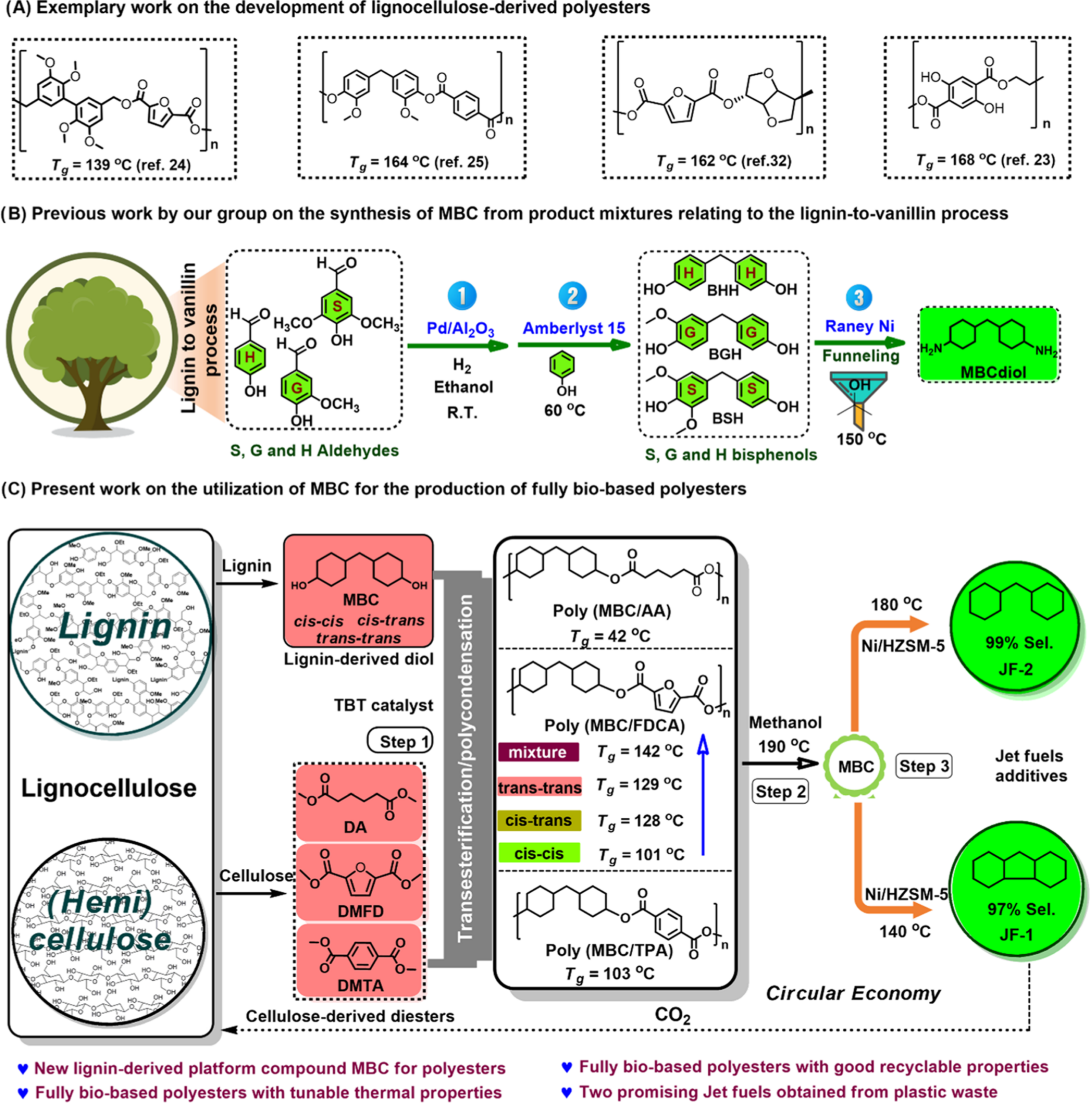
Overview of bio-based polyesters derived from lignocellulose: (A) representative pioneer work on the development of fully lignocellulose-derived polyesters; (B) our previous work on the synthesis of MBC from product mixtures relating to the lignosulfonate-to-vanillin process; and (C) overview of the here-presented work: step 1: copolymerization of MBC with the methyl esters of cellulose-derived TPA, FDCA, and AA, respectively, yielding poly(MBC/TPA), poly(MBC/FDCA), and poly(MBC/AA), step 2: methanolysis of the obtained MBC-based polyesters to their original monomers, and step 3: hydrodeoxygenation (HDO) of recovered MBC into promising jet fuel additives.

This remains a significant challenge as many fossil-resource-based plastics display moderate glass transition temperatures (*T*_g_), around or exceeding 90 °C,^18^ a feature not commonly found with bio-derived polymers.^19−22^ In this respect, notable examples, shown in Figure 1A, are the pioneering works by Short et al. on the design of poly(ethylene dihydroxyterephalates) with *T*_g_ values up to 168 °C.^23^ Llevot and co-workers reported on the copolymerization of a diol derived from vanillin with 2,5-furandicarboxylic acid (FDCA), yielding polyesters with *T*_g_ values up to 139 °C,^24^ and Curia et al. showed that polyesters made from lignin-derived bisguaiacols and suitable diesters could reach high *T*_g_ values (up to 164 °C) and high thermal stabilities (>300 °C, Figure 1A).^25^

Here, we aimed for the utilization of a new lignin-derived building block MBC, in the development of novel, recyclable, and fully bio-based plastic alternatives. MBC is an aliphatic, bicyclic rigid diol, which has been previously obtained by our group^26^ from industrially relevant product mixtures relating to the lignosulfonate-to-vanillin process (Figure 1B).^27−30^ Its synthesis entails the selective catalytic hydrogenation of prepurified aromatic aldehydes into their corresponding benzyl alcohols using Pd/Al_2_O_3_, followed by an Amberlyst-15-mediated coupling of the latter compounds with phenol, yielding a mixture of bisphenols, and the subsequent selective Raney nickel-catalyzed demethoxylation/hydrogenation of these bisphenol mixtures into the single aliphatic diol MBC, the latter constituting a catalytic funneling strategy.

It has been reported that polyesters made with cyclic monomers tend to have rigid molecular chains and hence display higher *T*_g_ values.^31−35^ Exemplary are the FDCA/isosorbide, pimeloyl chloride/betulin, and FDCA/1,4-cyclohexanedimethanol (CHDM)/2,2,4,4-tetramethyl-1,3-cyclobutanediol (CBDO) polyesters, which hold respective *T*_g_ values of 162,^32^ 165,^31^ and 103 °C (Figure 1A).^35^ Given the symmetric bicyclic nature of MBC and its inherent aliphatic alcohol functionalities, we assumed that this bio-derived building block could hold great potential for the synthesis of high*-T*_g_ bio-derived polyesters. Moreover, the possibility of using different MBC isomers could be valuable for the development of polymers with tunable properties. Less well developed, yet with relevance to biomass-derived monomers, is the stereochemical enhancement of polymer properties. This comprises main-chain stereochemistry, stereocomplexation, and cis–trans isomerism. Exemplary to the latter is the general observation that the higher trans content of aliphatic ring-containing monomers (e.g., CHDM; 1,4-cyclohexanedicarboxylic acid; 1,4-diaminocyclohexane) in a polymeric chain tends to give higher crystallinity, *T*_g_, and *T*_m_.^36^

Based on the above, we set out for the synthesis of unique, fully bio-based polyesters composed of MBC (Figure 1C)^6−13,37−42^ and cellulose-derived methyl esters of terephthalic acid (TPA),^14,15^ FDCA,^17^ or adipic acid (AA).^43^ The former two polymers display industrially interesting *T*_g_ values in the 103–142 °C range. Additionally, all here-presented polymers are characterized by high melting/decomposition temperatures in the 260–365 °C range, parameters which are also markedly influenced by the type of MBC isomer used. Polymers displaying both a *T*_g_ and a melting point *T*_m_ are classified as semicrystalline.

Finally, we subjected the prepared polyesters to methanolysis and demonstrated efficient recovery of the individual monomers. Moreover, a catalytic strategy for the conversion of recovered MBC to two promising bio-based jet fuel additives is being described.^44−49^ Given the high GHG emissions linked to aviation, and the industry’s desire to reduce these by half by 2050, this is currently an important topic.^50^ Overall, this work presents versatile, property-tunable, and recyclable fully bio-based polyesters, which display excellent thermal properties and thus hold great promise for future industrial applications.

## Experimental Section

### Preparation of the Ni/HZSM-5 Catalyst

The synthesis of a 20 wt % Ni/HZSM-5 catalyst was performed by a simple impregnation method. In a typical procedure, a solution containing 8.5 mmol (2.392 g) of Ni(NO_3_)_2_·6H_2_O in deionized water (5 mL) was dropwise added to a solution containing 2 g of the activated HZSM-5 support in water (5 mL) at 40 °C under vigorous stirring overnight. The resulting composite was dried in an oven at 100 °C overnight and calcined in a furnace at 550 °C in air for 4 h. The catalyst was then reduced in H_2_ flow at 550 °C for 2 h in a tube furnace. The obtained Ni/HZSM-5 catalyst was characterized by XRD, NH_3_-TPD, and SEM.

### Synthesis of Renewable Polyesters from the MBC Diol

The two-step melt polymerization (esterification and polycondensation) was performed using an equal molar ratio of the MBC diol and the comonomers in the presence of tetrabutyl titanate (TBT) as a catalyst. In short, a 100 mL three-neck flask was charged with 2.5 mmol of MBC, 2.5 mmol of dimethyl terephthalate (DMTA), and 1 mol % TBT catalyst, equipped with a magnetic stirrer and a reflux condenser. The esterification reaction was performed at 190 °C/N_2_ for 1 h under a nitrogen flow. Then, the reaction temperature was increased to 230 °C, and the pressure was slightly reduced to 1 mbar using an oil pump for 1 h. After that, the reaction mixture was cooled to RT, and the pressure was returned to atmospheric pressure by the introduction of nitrogen gas. The obtained solid was dissolved in CHCl_3_ and subsequently precipitated in an excess of methanol to yield purified polymers, which were characterized by NMR, GPC, DSC, TGA, and FTIR spectroscopy.

### Methanolysis of the Synthesized Polyesters

The mild depolymerization of the synthesized poly(MBC/TPA), poly(MBC/FDCA), and poly(MBC/AA) was carried out in a 100 mL high-pressure Parr autoclave, equipped with an overhead stirrer. Typically, the autoclave was charged with the polymer (0.2 g) and methanol (30 mL). The reactor was sealed, and pure nitrogen was used to flush the reactor three times. The reactor was heated to 190 °C and stirred at 400 rpm for 4 h. After the reaction, the reactor was cooled to room temperature. Then, the reaction mixture was concentrated under reduced pressure. The depolymerized crude mixture was purified by silica gel column chromatography (gradient elution dichloromethane/methanol = 100:0 to 80:20).

### Hydrodeoxygenation (HDO) of MBC into JF-1 and JF-2

The HDO of MBC was performed in a 100 mL high-pressure Parr autoclave, equipped with an overhead stirrer. Typically, the Parr autoclave was charged with MBC (1 mmol), 50 mg of the Ni/HZSM-5 catalyst, dodecane (10 mg), and methanol (30 mL). The reactor was sealed and pressurized with 30 bar H_2_. The reactor was heated and stirred at 400 rpm for 4 h. After the reaction, the reactor was cooled to room temperature. Then, 0.1 mL of solution was collected with a syringe and injected into the GC-MS or GC-FID after filtration with a PTFE filter (0.42 μm). The yield to JF-1 (2,3,4,4a,4b,5,6,7,8,8a,9,9a-dodecahydro-1H-fluorene) and JF-2 (dicyclohexylmethane) was calculated based on a flame ionization detector (FID), with the response factors having been estimated by the effective carbon number method (ECN).

## Results and Discussion

### Analysis and Characterization of Lignin-Derived MBC

**Figure 2:**
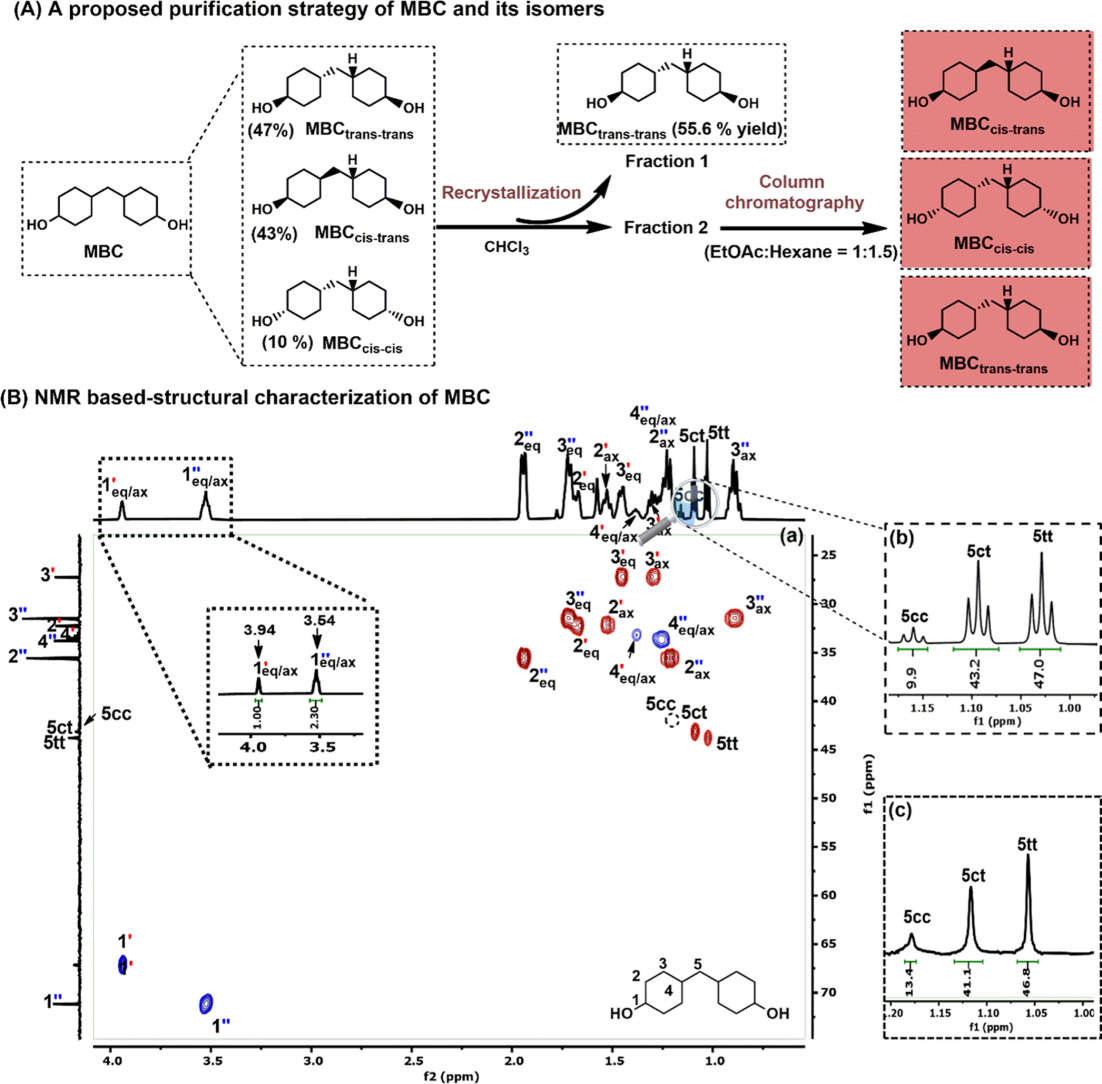
Synthesis, purification, and analysis of MBC and its isomers. (A) The production and isolation of MBC and its isomers. (B) NMR-based structural characterization of MBC: (a) 2D HSQC characterization of the MBC isomer mixture, (b) determination of the MBC isomer ratio in regular ^1^H NMR by means of MBC’s bridging methylene protons, and (c) determination of the MBC isomer ratio by means of ^1^H pure shift NMR. 1**′** refers to 1cis–cis and 1cis–trans and 1**″** refers to 1trans–trans and 1cis–trans. eq stands for equatorial H and ax stands for axial H. More information on the NMR characterization of MBC is available in the Supporting Information.

Previously, we have described catalytic strategies to obtain the bicyclic aliphatic diol (MBC) from the lignosulfonate-to-vanillin process via a series of efficient reaction steps involving catalytic funneling, as well as the use of MBC for the construction of novel bio-based polybenzoxazines. Herein, we set out to explore the potential of this unique, semi-rigid building block for the production of bio-based polyesters. As MBC exists as a mixture of three geometrical isomers, notably cis–cis, cis–trans, and trans–trans MBC (Figure 2A), an in-depth NMR characterization was performed. Given the appreciable complexity of the ^1^H NMR spectrum of MBC (Figures 2B and S1), with virtually all signals showing extensive coupling and/or partial-to-full overlap, the unequivocal determination of the MBC isomer ratio was found challenging. In this respect, the cyclohexane bridging methylene protons proved most instructive, as they appeared as relatively isolated multiplets in the 1–1.2 ppm range (Figure 2B). From these methylene signals, the relative composition of the original MBC isomer mixture could be determined as 10:43:47, respectively referring to cis–cis/cis–trans/trans–trans MBC isomers (Figure 2B-b). This was further confirmed using ^1^H pure shift NMR, an NMR technique, which applies broad-band decoupling to enhance the resolution of proton spectra by removing all homonuclear scalar couplings, the direct result being the collapse of complex multiplets into singlets (Figure 2B-c).^51,52^ The use of ^1^H pure shift NMR strongly benefits correct integration, especially of the low-intensity MBC_cis–cis_ signals.

Interestingly, MBC_trans–trans_ could be easily isolated in excellent purity and moderate yield (55.6%), by recrystallization from CHCl_3_. The full NMR spectroscopic characterization of pure MBC_trans–trans_ is given in Figures S3–S5. Separation of the MBC_cis–cis_ and MBC_cis–trans_ isomers was found possible using column chromatography. The full NMR spectroscopic characterization of the pure MBC_cis–cis_ and MBC_cis–trans_ isomers is shown in Figures S6–S11. Lastly, the unequivocal assignment of the trans–trans, cis–cis, and cis–trans connotations to the pure MBC isomers was performed using nuclear overhauser effect spectroscopy (NOESY), summarized in the Supporting Information.

### Synthesis, Analysis, and Characterization of Fully Bio-Based Polyesters Using MBC as the Starting Material

**Figure 3:**
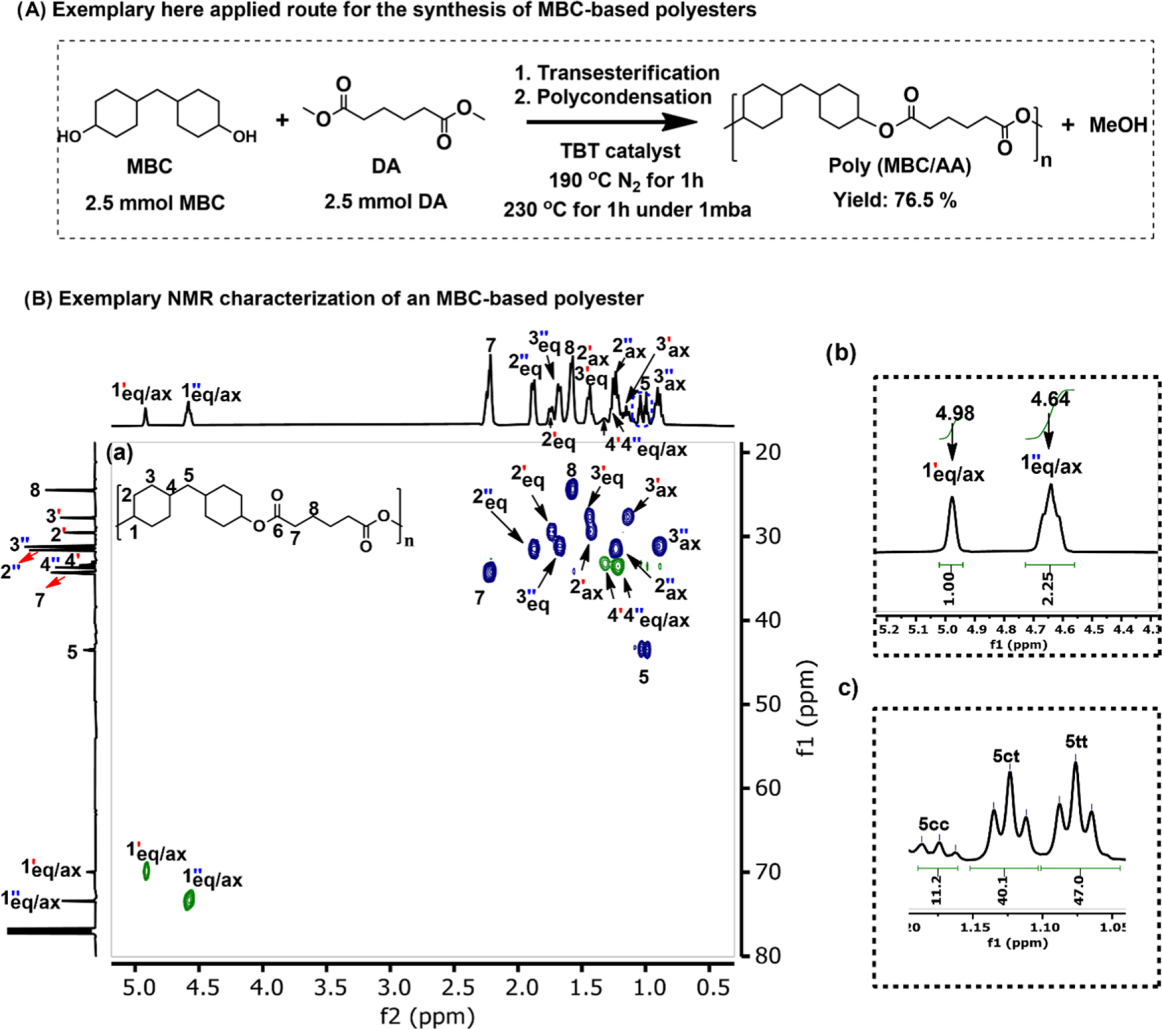
General Synthetic procedure and structural characterization for poly(MBC/AA). (A) Exemplary depiction of the here applied synthesis to poly(MBC/AA). (B) NMR characterizations of poly(MBC/AA): (a) 2D HSQC of poly(MBC/AA), (b) integration of the ^1^H C**H**–O NMR signals in poly(MBC/AA), and (c) ^1^H NMR-based determination of the MBC isomer ratio in poly(MBC/AA) by means of the bridging methylene protons (5H). The NMR characterizations of poly(MBC/TPA) and poly(MBC/FDCA) are available from the Supporting Information.

Next, a range of bio-based polyesters were prepared by solvent-free titanium-catalyzed transesterification^53^ and polycondensation of MBC (as a mixture of isomers) with three different cellulose-derived diesters, namely dimethyl terephthalate (DMTA), dimethyl furan-2,5-dicarboxylate (DMFD), and dimethyl adipate (DA). These are, respectively, denoted as poly(MBC/TPA), poly(MBC/FDCA), and poly(MBC/AA). Figure 3A shows the here applied polymerization procedure for the specific case of poly(MBC/AA). The choice of Ti as the polymerization catalyst was inspired by its general high activity in polyester formation and the existence of ample geological reserves of this metal.^54^ As shown in Table 1, the latter polymers were obtained in good-to-excellent numerical yields, spanning the 65–94% range. FTIR analysis of the final polymeric products confirmed successful polyesterification with the clear absence of the MBC-related diol moiety (i.e., OH stretching vibration at 3200–3500 cm^–1^)^25^ and the distinct presence of an ester carbonyl stretching band at around 1720 cm^–1^, as shown in Figure S54.^25^ A full structural analysis of the different synthesized polymers by various NMR spectroscopic methods (^1^H NMR, ^13^C NMR, 2D HSQC, and 2D COSY) is discussed in the Supporting Information and specifically shown in Figures S16–S35. Most instructively, upon polymerization, the MBC ^1^H C**H**–OH signals at 3.54 ppm (cis–cis; cis–trans) and 3.94 ppm (cis–trans; trans–trans; Figure 2B-a) undergo a remarkable downfield shift beyond 4.5 ppm (Figure 3B-a). More specifically, the respective ^1^H C**H**–O signals of poly(MBC/TPA), poly(MBC/FDCA), and poly(MBC/AA) were recorded at 4.93/5.28 ppm (Figure S29), 4.90/5.22 ppm (Figure S25), and 4.64/4.98 ppm (Figure 3B-b). Such a downfield shift of these respective ^1^H NMR signals is in line with the formation of ester bonds, therewith confirming effective and successful polymerization. By comparing Figures 2B-a and 3B-b, the relative ratio of the C**H**–O peaks remains the same at 1:2.3, suggesting non-preferential incorporation of the MBC isomers in the polymer chain. Convincingly, integration of MBC’s bridging methylene protons in poly(MBC/AA) shows a relative ratio of (11:40:47; Figure 3B-c), which is perfectly in line with the previously determined MBC isomer ratio (Figure 2B-b and c).

**Table 1:** Molecular-Weight Distributions and Thermal Property Data for Synthesized Polyesters^a^

entry	products	yield^b^ [%]	*M*_w_^c^ [g·mol^–1^]	*M*_n_^c^ [g·mol^–1^]	*Đ*	*T*_m_^d^ [°C]	*T*_d_^d^ [°C]	*T*_g_^e^ [°C]
1	poly(MBC/TPA)	93.9	8270	2890	2.9	261	272	103
2	poly(MBC/FDCA)	84.7	18 500	10 300	1.8	275	284	142
3	poly(MBC_cis-cis_/FDCA)	66.4	10 600	4630	2.3	263	277	101
4	poly(MBC_cis-trans_/FDCA)	77.8	10 200	5240	1.9	290	294	128
5	poly(MBC_trans-trans_/FDCA)	65.1	8230	4340	1.9	325	342	129
6	poly(MBC/AA)	76.5	25 400	9700	2.6	365 (broad)		42

a Reaction conditions: 2.5 mmol of diol, 2.5 mmol of comonomers, 1 mol % titanium (IV) butoxide (TBT) catalyst, 190 °C N_2_/1 h, 230 °C/1 h under vacuum 1 mbar.

b Yield (%) = weight of the collected product/weight of the theoretical product.

c Molecular weight distribution was determined by GPC.

d *T*_m_ = melting temperature and *T*_d_ = temperature of decomposition—as determined by TGA/DSC characterization.

e *T*_g_ was determined by DSC characterization.

GPC analysis revealed Mw values between 8000 and 26 000 g mol^–1^, proving effective polymerization (Table 1 and Figures S48–S53). The comparison of the *M*_w_ values and the obtained yields is challenging, as polycondensation is very sensitive to stoichiometry and hence to the purity of the involved monomers, and this determines the true reaction stoichiometry. Indeed, while MBC_trans–trans_ is highly pure due to the crystallization process, MBC_cis–trans_ and MBC_cis–cis_ are more challenging to separate from the other MBC isomers and residual solvents, hence representing a somewhat lower purity. Further optimizations of the reaction conditions will be carried out in the future as to obtain high Mw polyesters from which the mechanical properties can be determined.

The DSC analysis of poly(MBC/TPA) and poly(MBC/FDCA) revealed respective *T*_g_ values of 103 and 142 °C (Table 1, entries 1 and 2). In addition, the TGA/DSC analysis of the latter two polymers showed respective melting temperatures (*T*_m_) of 261 and 275 °C, and respective decomposition temperatures (*T*_d_) of 272 and 284 °C, further underscoring their potential industrial relevance.

The influence of the MBC pure isomers on the thermochemical properties was investigated for the poly(MBC/FDCA) case (Table 1). It was found that polymers made with a pure MBC isomer showed a lower glass transition temperature (*T*_g_) compared to the original poly(MBC/FDCA) (Figures S38–S41). More specifically, the *T*_g_ decreased along the following series: poly(MBC/FDCA) [*T*_g_ = 142 °C] > poly(MBC_cis–trans_/FDCA) ≈ poly(MBC_trans–trans_/FDCA) [*T*_g_ = 128–129 °C] > poly(MBC_cis–cis_/FDCA) [*T*_g_ = 101 °C], the latter *T*_g_ value representing a drop of 41 °C compared to that of poly(MBC/FDCA). Apart from poly(MBC_cis–cis_/FDCA), the use of pure MBC isomers increased the melting temperature (*T*_m_) and the decomposition temperature, the trend being: poly(MBC_cis–cis_/FDCA) [*T*_m_ = 263 °C; *T*_d_ = 277 °C] < poly(MBC/FDCA) [*T*_m_ = 275 °C; *T*_d_ = 284 °C] < poly(MBC_cis–trans_/FDCA) [*T*_m_ = 290 °C; *T*_d_ = 294 °C] < poly(MBC_trans–trans_/FDCA) [*T*_m_ = 325 °C; *T*_d_ = 342 °C] (Figures S42–S47). Thus, the use of pure MBC isomers shifted both *T*_m_ and *T*_d_, respectively, from 263 to 325 °C (62 °C difference) and from 277 to 342 °C (65 °C difference).

Furthermore, poly(MBC_trans–trans_/FDCA) and poly(MBC_cis–trans_/FDCA) effectively show higher *T*_m_ values than poly(MBC/FDCA). This indicates that the relative stereochemistry (trans–trans, cis–trans, and cis–cis) in the MBC monomer plays a distinctly important role, although the molecular weight values of the respective polymers are also different.

Interestingly, the TGA/DSC analysis of these polymers generally reveals a dual peak around the decomposition temperature (Figures S42–S47), the exception being poly(MBC/AA) where it concerns a broad decomposition peak (Figure S47). This hints at the effective existence of a melting point, be it though very closely situated to the decomposition point. In this respect, XRD analysis confirmed the existence of semicrystallinity in all analyzed polymers (Figure S55).^55^ It is further noteworthy that the XRD patterns of poly(MBC/TPA) and poly(MBC_trans–trans_/FDCA) show a more pronounced fine structure, indicating a more developed crystallinity. As to poly(MBC_trans–trans_/FDCA), this is in line with the general literature statement that with aliphatic cyclic monomers, higher trans contents in the polymeric chain will display higher crystallinity.^36^ Of note here is also that the MBC_trans–trans_ isomer is most prone to crystallization.

Nearly all here synthesized polymers are soluble in THF and chloroform, the exception being poly(MBC_trans–trans_/FDCA) which is only soluble in chloroform. This is tentatively explained by the higher crystallinity of poly(MBC_trans–trans_/FDCA), which is potentially the result of additional extensive furan interactions between the polymer chains. In this respect, it is noteworthy that the furan ring has a dipole moment of 0.70 Debye, which favors dipolar interactions.^56^ The insolubility of polymers engaged in extensive interchain interactions has been observed before, for instance, with aromatic polyboronates.^57^

Poly(MBC/AA) displayed a low *T*_g_ of 42 °C, which is attributed to the more flexible AA chain. Poly(MBC/AA) is also semicrystalline in nature, which is in line with other fully aliphatic polyesters containing AA,^58^ and it displays a high decomposition temperature (*T*_d_) of 365 °C (Table 1, entry 6).

### Potential for Practical Applications and Comparisons with Other Polymer Classes

**Figure 4:**
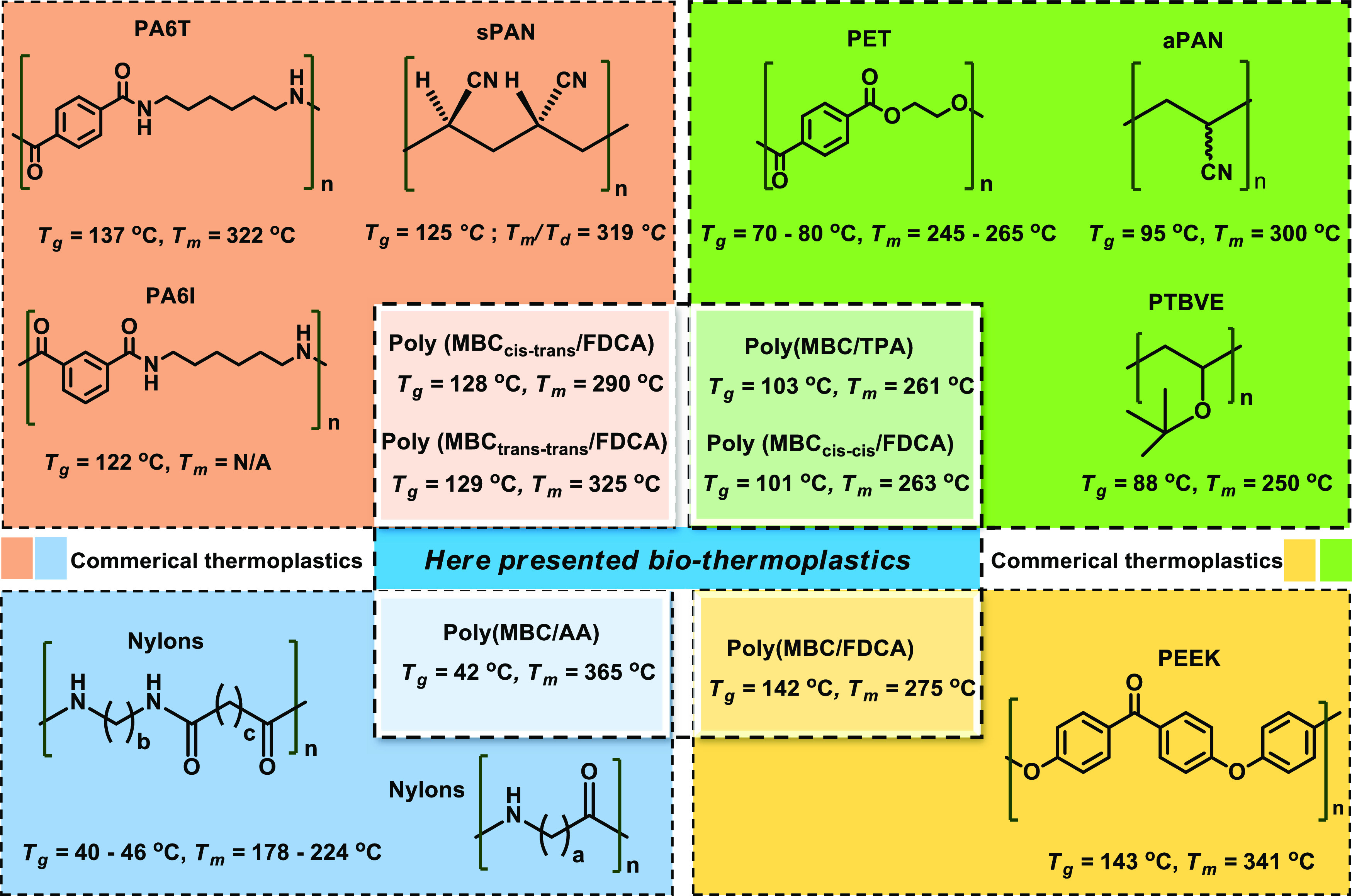
Survey of the potential substitution of certain polymer types by the here-developed MBC-based polyesters.

Due to the unique steric properties of the lignin-derived MBC building block, many of the here-presented polyesters display interesting properties, pointing toward very promising practical applications, even with respect to different classes of polymers, as summarized in Figure 4. Thus, it is noteworthy that the *T*_g_ and *T*_m_ of poly(MBC/TPA) and poly(MBC_cis–cis_/FDCA) are close to the ones of polyethylene terephthalate (PET),^59^ atactic polyacrylonitrile (aPAN),^60,61^ and poly(*tert*-butyl vinyl ether) (PTBVE).^62^ The *T*_g_ and *T*_m_ of poly(MBC_trans–trans_/FDCA) and poly(MBC_cis–trans_/FDCA) resemble those of syndiotactic polyacrylonitrile (sPAN)^62^ but equally those of the heat-resistant polyamides poly(hexamethylene teraphthalamide) (PA6T)^63^ and poly(hexamethylene isophthalamide) (PA6I).^64^ Moreover, a wide variety of PA6T copolyamides with *T*_g_ values between 90 and 141 °C and *T*_m_ values between 235 and 325 °C have been reported,^65^ spanning the same *T*_g_ and *T*_m_ ranges of the here-presented MBC-based polymers. Finally, poly(MBC/FDCA) has a similar *T*_g_ as polyether ether ketone (PEEK),^66^ and poly(MBC/AA) resembles a range of polyamides (Nylons) such as nylon 11,^67,68^ nylon 12,^68,69^ and nylon 6/10.^68^

Thus, the here-developed sustainable polymers can mimic the thermal properties (*T*_g_, *T*_m_, *T*_d_) of certain N-containing polymers (polyamides, polyacrylonitrile), yet by only involving C, H, and O atoms in the polymeric backbone. For a classic thermoplastic material, a melting point near the decomposition point may be a limitation. However, as the polymers are soluble, applications such as fibers are well within reach. In this respect, it is also noteworthy that the major use of PET is as fibers (2021: 60.5 Mio t fibers^70^ vs 24.3 Mio t plastics^71^), witnessing the PET fiber trademark names Dacron (DuPont) and Terylene (Imperial Chemical Industries Ltd.).^72^

### Recycling and Upcycling Strategies

**Figure 5:**
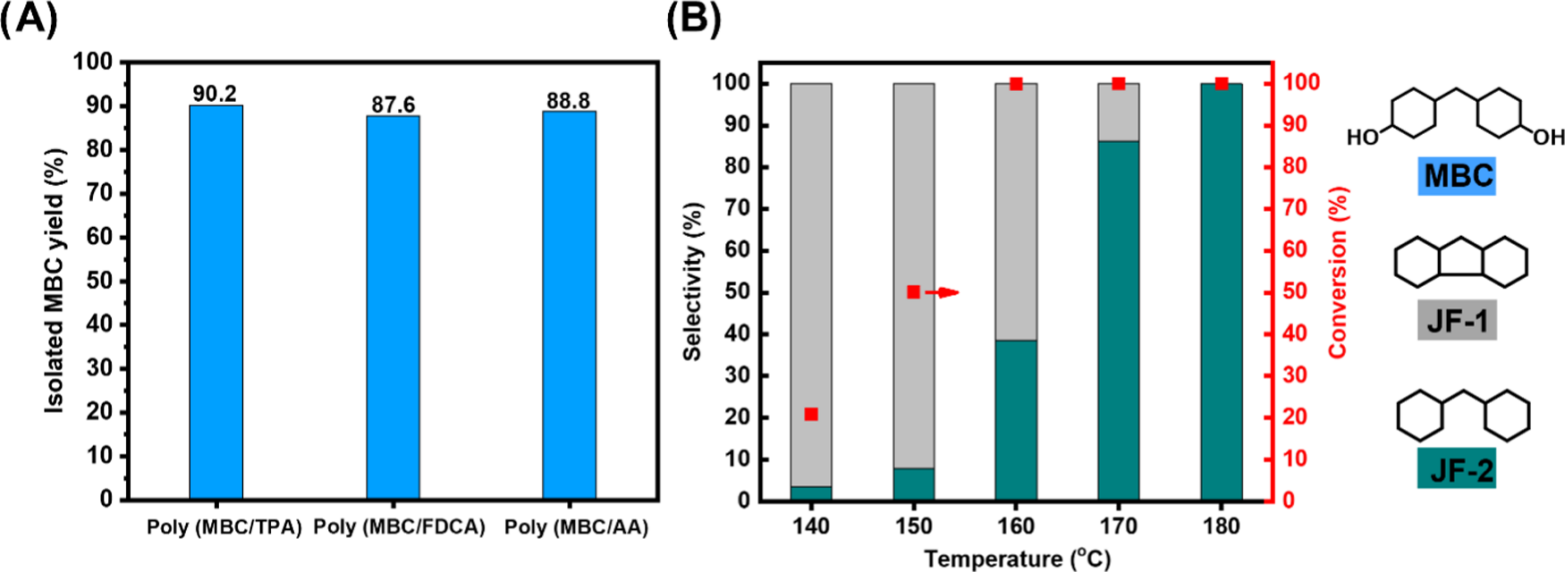
(A) Methanolysis of poly(MBC/TPA), poly(MBC/FDCA), and poly(MBC/AA). Reaction conditions: 200 mg of polymer, 30 mL of methanol, 190 °C, 4 h, 1 bar N_2_, 10 mg of dodecane. (B) Influence of the reaction temperature on the hydrodeoxygenation of MBC over the Ni/HZSM-5 catalyst. Reaction conditions: 1 mmol of MBC, 30 mL of cyclohexane, 10 mg of dodecane, 4 h. The conversion levels were determined via a calibration curve. The selectivities were determined by the effective carbon number (ECN) method.^75^

With polymer recyclability being very important, we also investigated the proneness of the here-developed polyesters to depolymerization. It was found that all here-presented polymers could be fully and easily degraded into their respective monomers by methanolysis, without the deliberate addition of additives. That way, MBC could be recovered in 90% yield (Figure 5A), independent of the type of polymer (for representative GC-FID traces, see Figure S56). This is an important observation as the above-mentioned PTBVE, PAN, and PEEK polymeric materials are not prone to facile degradation/recycling, since the synthesis of PTBVE and PAN involves C–C bond formation through, respectively, cationic vinyl polymerization^73^ and radical polymerization,^74^ while in the case of PEEK, highly stable diphenyl ether bonds are created.

**Figure 6:**
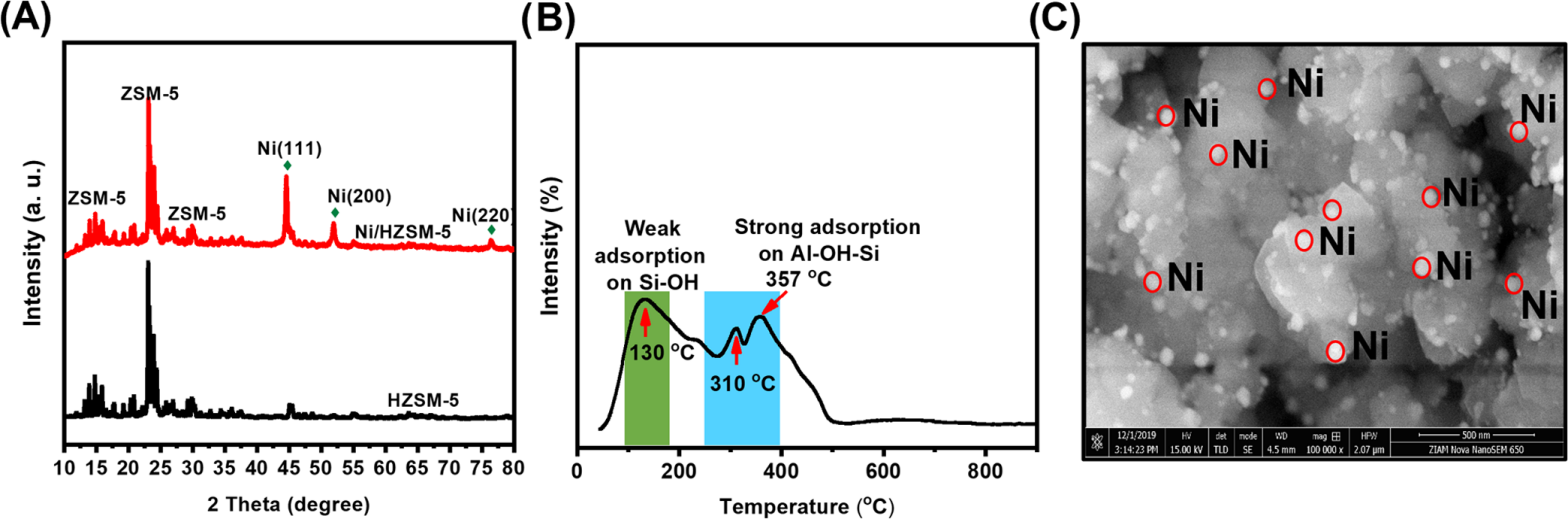
General characterization of the Ni/HZSM-5 catalyst: (A) X-ray diffraction (XRD) analysis, (B) temperature-programmed desorption with NH_3_ (NH_3_-TPD), and (C) scanning electron microscopy (SEM) image of the Ni/HZSM-5 catalyst. The marked white spots are nickel nanoparticles.

Alternatively, we also investigated the catalytic transformation of (recovered) MBC into a valuable and high-energy dense jet fuel. For that purpose, MBC was subjected to consecutive dehydration/hydrogenation steps over an in-house prepared Ni/HZSM-5 catalyst. The applied Ni/HZSM-5 catalyst was extensively characterized using XRD (Figure 6A), NH_3_-TPD (Figure 6B), and SEM (Figure 6C). As shown in Figure 6B, NH_3_-TPD reveals the presence of both weak and strong acid sites. More specifically, the desorption peak in the 100–200 °C region points at the weak adsorption of NH_3_ on Si–OH Brönsted acid centers,^76^ while the desorption peak of NH_3_ in the 300–400 °C region can be attributed to the strong adsorption of NH_3_ on mainly Al–OH–Si.^74^ The XRD analysis of the Ni/HZSM-5 catalyst reveals the clear presence of distinct diffraction peaks at 2θ = 44, 53, and 77°, which points to the presence of a pure nickel metal crystal phase (Figure 6A),^77^ and SEM (Figure 6C) reveals the presence of highly dispersed Ni nanoparticles, which can easily engage in the hydrogenation of dienes.

It was found that at 140 °C, MBC could be selectively (97%) converted into perhydrofluorene (JF-1; IUPAC name = 2,3,4,4a,4b,5,6,7,8,8a,9,9a-dodecahydro-1H-fluorene) in 20% conversion (Figures 5B and S57a). Additionally, the operation of this reaction at 180 °C yielded dicyclohexylmethane (JF-2) quantitatively as the sole product (>99% yield) (Figures 5B and S57b). This is a most interesting observation, as the density of JF-1 (0.96 g mL^–1^) is markedly higher than that of JF-2 (0.88 g mL^–1^), even exceeding the density of the most performing JP-10 jet fuel available to date (0.94 g mL^–1^).^78^ The higher density of JF-1 vis-à-vis JF-2 explains the higher heat value of the former (40.1 MJ L^–1^) versus the latter (36.8 MJ L^–1^).^79^ While the specific catalytic formation of JF-1 and JF-2 from MBC has not been reported to date, the concurrent occurrence of JF-1 and JF-2 has been reported by Nie et al. in a study on the hydrogenation/ hydrodeoxygenation of 2-benzylphenol (2BP),^79^^79^ where it was found that 2BP was first, and invariably, hydrogenated over Pd/C to 2-benzylcyclohexanol (2BCH). The further presence of an acidic zeolite (e.g., HZSM-5) then transforms 2BCH into JF-2, while the sole application of Pd/C affects 2BCH ring closure to JF-1.^79^ Conversely, the here-observed Ni/HZSM-5-catalyzed formation of JF-1 (from MBC) runs at a lower reaction temperature and involves an HZSM-5-mediated dehydration of MBC to a set of dienes and a Ni-mediated ring closure.^80^ Separate application of sole HZSM-5 to MBC showed only the formation of a range of dienes (Figure S59). Application of a higher temperature predominately led to JF-2 because of rapid hydrogenation of any unsaturated intermediates precluding any cyclization. On the potential of JF-1 and JF-2 as neat unblended jet fuels, the currently available literature lists unfavorable freezing points (JF-1 at −20 °C) and viscosities.^79^ However, 50/50 blending of JF-1 with JP-10 has been shown to give a high-performance jet fuel with favorable properties, notably, a density of 0.95 g mL^–1^ (20 °C), a viscosity of 17.4 mm^2^ s^–1^ (20 °C), and a freezing point below −75 °C.^79^ In this respect, it is also noteworthy that JP-10 is very expensive (7091 $/ton) and only available in limited quantities,^46^ making a favorable case for blending with a potentially less expensive fuel. All this underscores the relevance of bio-derived JF-1 as a high-density additive to jet fuels and hence also the practical value of this reconversion strategy in relation to establishing a viable circular strategy for our high-performance, bio-based polyesters.

## Conclusions

In this paper, we presented the development and characterization of a versatile set of bio-based semicrystalline polyesters, which due to their high *T*_g_ and *T*_m_/*T*_d_ values could offer sustainable alternatives to atactic/syndiotactic polyacrylonitrile (PAN), polyether ether ketone (PEEK), polyethylene terephthalate (PET), poly(*tert*-butyl vinyl ether) (PTBVE), and certain classes of polyamides (high-heat-resistant ones and nylon). Central to this invention is the usage of a bio-based bicyclic aliphatic diol monomer (MBC), which can impart interesting properties to the obtained thermoplastic products, and the isomerism of which is capable of tuning the polymer properties. Indeed, it was found that the use of pure MBC isomers, vis-à-vis the original MBC mixture, allowed for fine-tuning of the *T*_g_ and *T*_m_/*T*_d_ values. The limitation of close melting and decomposition temperatures for the here-presented polymers is mitigated by polymer solubility, which opens the possibility of fiber applications. From a sustainability point of view, the presented polyesters are balanced, with MBC being directly derived from lignin and the diester being cellulose-derived. Follow-up studies of mechanical properties will shed further light on the further capability of the here-presented polymers to substitute for any of the before-mentioned polymers/polymer classes in specific applications. All polymers can be efficiently depolymerized using methanolysis, yielding recovered MBC in up to 90% isolated yield. While methanolysis is a commonly applied method to the depolymerization of polyesters, this is nonetheless a most interesting achievement, as some of the here-presented polyesters could potentially rival polymer classes, which are not susceptible to methanolysis. Alternatively, the efficient conversion of MBC to a competitive bio-derived aviation fuel additive was also described. Overall, this is an elegant example of a sustainable biorefinery concept, one with pluridisciplinary outputs and a clear recyclability vision.

## Supporting Information Available

Supplementary experiment sections; result and discussion; raw 2D HSQC NMR spectra; mass spectra of the identified compounds; and other supplementary tables and figures (PDF)
