# Supplementary material for: High-Performance Thermoplastics from a Unique Bicyclic Lignin-Derived Diol
Source: ACS Sustain Chem Eng. 2023 Feb 6;11(7):2819–29. doi: 10.1021/acssuschemeng.2c05998 (PMC9945171; doi:10.1021/acssuschemeng.2c05998)
Supplement: Supplementary file 1 — sc2c05998_si_001.pdf [file sc2c05998_si_001.pdf]

Supporting information

High-Performance Thermoplastics from a Unique Bicyclic Lignin-derived diol

Xianyuan Wu [†]*, Mario De bruyn* [‡]*, Gregor Trimmel*[§]*, Klaus Zangger*[‡] *and Katalin Barta*[†‡]*

[†]Stratingh Institute for Chemistry, University of Groningen, Nijenborgh 4, 9747 AG Groningen Groningen, The Netherlands.

[‡]Department of Chemistry, Organic and Bioorganic Chemistry, University of Graz, Heinrichstrasse 28/II, 8010, Graz, Austria

[§]Institute for Chemistry and Technology of Materials (ICTM), NAWI Graz, Graz University of Technology, Stremayrgasse 9, 8010, Graz, Austria.

*Correspondence: katalin.barta@uni-graz.at

Number of pages: 37

Number of tables: 1

Number of figures: 59

Table of contents

1. General information S3

1.1 Materials and reagents S5

1.2 Preparation of the model compounds S5

1.3 Conversion, selectivity and yield calculation S5

2. Experimental section S7

Table S1 Recent advances on the development of FDCA based polyesters with different T_g value S7

Figure S1-S11 NMR spectra of MBC and its isomers S8

Figure S12-S15 NMR-based structural determination of the cis/trans configurations of the pure MBC isomers S14

Figure S16-S35 NMR spectra of poly(MBC/TPA), poly(MBC/FDCA) and poly(MBC/AA) S17

Figure S36-S41 DSC thermograms of poly(MBC/TPA), poly(MBC/FDCA) and poly(MBC/AA) S27

Figure S42-S47 TGA plots of poly(MBC/TPA), poly(MBC/FDCA) and poly(MBC/AA) S30

Figure S47-S53 GPC traces of poly(MBC/TPA), poly(MBC/FDCA) and poly(MBC/AA) S33

Figure S54 FTIR spectroscopy of poly(MBC/TPA), poly(MBC/FDCA) and poly(MBC/AA) S35

Figure S55 XRD patterns of poly(MBC/TPA), poly(MBC/FDCA) and poly(MBC/AA) S35

Figure S56 GC-FID traces of crude product mixtures obtained from methanolysis of the poly(MBC/TPA), poly(MBC/FDCA) and poly(MBC/AA) S36

Figure S57-S58 GC-FID/MS traces of crude hydrodeoxygenation mixture of MBC over Ni/HZSM-5 catalyst S36

Figure S59 GC-FID traces of crude hydrodeoxygenation mixture of MBC over HZSM-5 catalyst S37

Supplementary References **38**

1. General information

Column chromatography was performed using Merck silica gel type 9385 230–400 mesh and typically dichloromethane and methanol or EtOAc and pentane as eluent.

Thin layer chromatography (TLC): Merck silica gel 60, 0.25 mm. The individual components were visualized by UV or $KMnO_4$ staining.

Gas Chromatography (GC) was used for product identification as well as determination of conversion and selectivity values. Product identification was performed by GC-MS (5975C MSD) equipped with an HP-5 MS column, and helium as carrier gas.The temperature program started at 50 °C for 5 min, heated by 10 $°C\cdot min^{-1}$ to 325 °C and held for 5 min. Conversion and products selectivity were determined by GC-FID (Agilent 8890 GC) equipped with an HP-5MS column using nitrogen as carrier gas.

Nuclear Magnetic Resonance (NMR) spectroscopy:

^{1}H, and ^{13}C NMR spectra were recorded on a Bruker Avance III 300 MHz (300 and 75 MHz, respectively) and 2D NMR spectra were recorded on a Bruker Avance III 700 MHz with Cryoplatform and a 5mm Triple-Resonance cryoprobe (700 and 175 MHz, respectively). ^{1}H,^{13}C NMR and 2D NMR spectra were recorded at RT. Chemical shift values are reported in ppm with the solvent resonance as the internal standard ($CDCl_3$: 7.26 for ^{1}H, 77.0 for ^{13}C; CD_3OD: 3.31 for ^{1}H, 49.0 for ^{13}C; DMSO-d_6: 2.50 for ^{1}H, 39.5 for ^{13}C). Data are reported as follows: chemical shifts, multiplicity (s = singlet, d = doublet, t = triplet, q = quartet, br. = broad, m = multiplet), coupling constants (Hz), and integration.

Structural determination of the conformation on the 6-membered rings of MBC: All NMR spectra were recorded on a Bruker Avance III 700 MHz NMR spectrometer equipped with a 5 mm TCI cryoprobe with z-axis gradients at 298 K. For the selectively decoupled 1D ^{1}H spectra we used continuous wave homodecoupling during acquisition. The ^{1}H-^{13}C HSQCs were recorded using a multiplicity-edited sensitivity-enhanced version with 32 scans for each of the 256 increments, amounting to 2.5 hours of instrument time. For the NOESY spectra 32 scans were accumulated for each of the 1024 transients (total instrument time 12 hours). All spectra were apodized using 60° phase shifted squared sine-bell window functions applied in spectral dimensions and zero filling to twice the number of acquired data points prior to Fourier transformation using TopSpin 3.1.

Gel Permeation Chromatography (GPC) was conducted at the Graz University of Technology on a Shimadzu instrument equipped with two separating columns from MZ-Gel SD plus (8×300 mm, 5µm) plus 1×precolumn (8×50mm, 5µm). The columns were operated at ambient temperature with a flow-rate of 1 $mL\cdot min^{-1}$ of chloroform. Detection was accomplished at ambient temperature using a RID-20A Differential Refractive Index Detector in series. The molecular weight determination was performed using polystyrene standards of known molecular weight distribution.

Differential Scanning Calorimetry (DSC) was conducted at the Graz University of Technology on a Perkin Elmer DSC 8500. In a typical procedure, the sample (5-10 mg) was weighed into a DSC aluminum pan and then capped with a lid. The sample was sealed and heated from 25 to 250 °C with a heating rate of 20 °C·min^{-1}. Then it was cooled to 25 °C with a heating rate of 20 °C·min^{-1}. Subsequently, a second heating scan to 250 °C with the same heating rate was performed. All of the experiments were performed under an N_2 flow with a flow rate of 20 mL·min^{-1}.

Simultaneous thermoanalysis (TGA/DSC) was performed at the Graz University of Technology on a Netzsch Jupiter STA 449C simultaneous thermoanalyzer and was used to determine T_D and T_m of the polymers. Typically, the sample (1-3 mg) was weighed into a platinum pan. The sample was heated from 20 to 550 °C with a heating rate of 10 °C·min^{-1} and a N_2 flow rate of 20 mL·min^{-1}. The temperatures were recorded when 5 % weight loss ($T_{5\%}$) and 90% weight loss rate ($T_{90\,\%}$) occurred.

Inductively coupled plasma mass spectrometry (ICP-MS) was performed at the University of Graz on an Agilent 7900 ICP-MS. Typically, the samples were solubilized with 5 mL HNO_3 in the MLS ultraclave and then heated to 250 °C for 30 mins before analysis by **ICP-MS.**

X-ray diffraction (XRD) was performed at the Graz University of Technology on a RIGAKU Miniflex 600 with D/Tex Ultra detector with a CuKα radiation (λ=1.5418 Å). XRD patterns were collected in reflection geometry in the 2-theta range between 0° and 30°.

Temperature-programmed adsorption with NH_3 (NH_3-TPD) was performed at the University of Groningen on a Micromeritics TPD 2900 apparatus, using 10 vol. % NH_3 in Ar. The Ni/ HZSM-5 sample were pretreated in He at 383 K for 1 h. After cooling down the catalyst was adsorbed by NH_3/Ar for 0.5 h at RT and the temperature program started at a ramping rate of 10 K min^{-1} to 1173 K. The gas from the reactor outlet was dewatered using a cold trap (isopropanol/liquid nitrogen) and subsequently analyzed by a TCD detector.

Scanning electron microscopy (SEM) images were recorded at the University of Groningen using a Philips XL-30-FEG at an accelerating voltage of 5 – 15 kV. Specimens were deposited as powders on aluminum pin flat stubs.

Attenuated total reflection-Fourier-transform infrared spectroscopy (FTIR) were performed at the University of Groningen using a VERTEX 70 spectrometer in the wave number range of 400-4000 cm^{-1} with a resolution of 4 cm^{-1}, equipped with an ATR geometry.

1.1 Materials and reagents

2,5-Furandicarboxylic acid (FDCA) (> 98.0%), dimethyl terephthalate (DMTA) (> 99.0%), diphenyl carbonate (DPC) (> 99.0%), dimethyl adipate (DA) (> 99.0 %), $Ni(NO_3)_2 \cdot 6H_2O$ (> 99.9 %), Titanium (IV) butoxide (TBT) (> 97.0%) were purchased from Sigma-Aldrich and TCI chemicals company. Chemicals were used as received, unless otherwise specified. NH_4-ZSM-5 with a Si/Al=40 ratio was purchased from Fisher Scientific and preactivated in a furnace at 550 °C in air for 6 h before use – hence yielding HZSM-5.

1.2 Preparation of the model compounds

Preparation of the MBC diol: The synthesis of 4,4-methylenebiscyclohexanol (MBC) was carried out according to a previously reported procedure.[1] In a typical procedure, a 1L high pressure Parr autoclave was charged with 2 g Raney nickel catalyst, 5 g bisphenol F, 100 mL isopropanol, and equipped with mechanical stirring. The reactor was sealed, purged 3 times with H_2 and then pressurized with H_2 (40 bar). The reactor was heated to 150 °C for 4 h under stirring at 400 rpm. After completion of the reaction, the reactor was cooled to RT. Then 0.1 mL solution was collected through a syringe and injected to GC-MS or GC-FID after filtration through a PTFE filter (0.45 µm). The Raney nickel was separated from the solution by centrifugation and subsequent decantation and additionally washed with isopropanol (3×30 mL). Then the isopropanol soluble fractions were combined and the solvent was removed under reduced pressure. The product 4, 4'-methylenebiscyclohexanol was obtained as a white solid (5.194 g, 24.5 mmol) in a 98% yield as a mixture of isomers (cis-cis: cis-trans: trans: trans with the ratio of 10: 43: 47), based on ^{1}H NMR.

Isolation of $MBC_{trans\text{-}trans}$ by recrystallization: In a typical procedure, a 100 mL round bottom flask, equipped with a magnetic stirring bar, was charged with 5 g of $MBC_{cis\text{-}cis:\ cis\text{-}trans:\ trans\text{-}trans}$ and 50 mL chloroform. The mixture was heated to 50 °C under stirring at 500 rpm until all MBC was solubilized. Then, the mixture was allowed to cool in the fridge for two days to yield solid crystals (2.5 g) by filtration. The pre-recrystallized solid crystals were subjected to secondary recrystallization to yield pure **$MBC_{trans\text{-}trans}$** (1.3 g).

1.3 Conversion, selectivity and yield calculation

i For copolymerization of MBC with DMTA, DMFD and DA

$$\text{Yield (\%)} = \frac{\text{Mass of the obtained polymers}}{\text{Mass of theoretically obtained polymers}} \times 100\%$$

ii For methanolysis depolymerization of polymers:

$$\text{Conversion (\%)} = \frac{\text{Mass of (original polymer} - \text{remaining polymer)}}{\text{Mass of initial polymer}}$$

$$\text{Monomers yield (\%)} = \frac{\text{Mass of the obtained MBC}}{\text{Theoretical mass of monomers from polymers}} \times 100\%$$

iii For calculation of yield to hydrocarbon alkanes

$$\text{F (R} - \text{wt)} = \frac{\text{Mw of MBC} \times \text{ECN of dodecane}}{\text{Mw of dodecane} \times \text{ECN of product}}$$

ECN of dodecane = 12 (carbon number)

ECN of product = Carbon number of hydrocarbons

$$\text{F (R} - \text{wt)} = \frac{\text{Peak area counts for dodecane} \times \text{wt. of products}}{\text{Peak area counts for products} \times \text{wt. of dodecane}}$$

Abbreviations

MBC: 4,4-methylenebiscyclohexanol

FDCA: 2,5-furandicarboxylic acid DMFD: dimethyl 2,5-furandicarboxylate

TPA: terephthalic acid DMTA: dimethyl terephthalate

DA: dimethyl succinate AA: adipic acid

TBT: titanium (IV) butoxide

HDO: hydrodeoxygenation

JF-1: 2,3,4,4a,4b,5,6,7,8,8a,9,9a-dodecahydro-1H-fluorene

JF-2: cyclohexyl-cyclohexane

2. Experimental section

Table S1. Recent advances on the development of FDCA based polyesters with different T_g value

Abbreviation	Chemical Structure	Catalyst	Tem. (°C)	T_g (°C)	Ref.
PEF	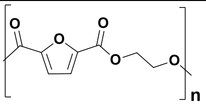	Sb_2O_3	240-250	80	2
PPF	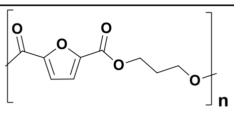	TBT TTIP	220-230	52	3
PBF	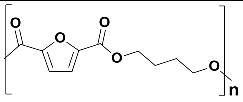	TBT TTIP	220-230	39	3
PCF	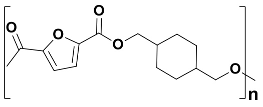	$Zn(OAc)_2$ Sb_2O_3	240-300	82	4
PIsF	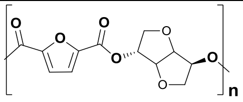	TBT	210-230	161.9	5
PMePF	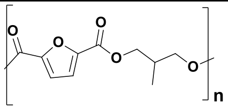	TBT	210-230	53	5
Poly(BG FDCA)	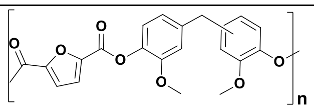	$SOCl_2$ DMF	80	157	6
PBFC	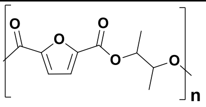	$Sn(Oct)_2$	220	113	7
P6	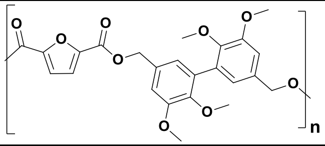	$Ti(OBu)_4$	200	139	8
PHF	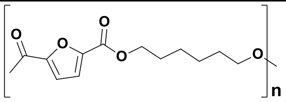	TBT	235	10	9
PPF	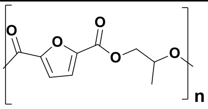	TBT	235	92	9
PNF	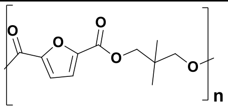	Sb_2O_3	220	73	10
PHMBF	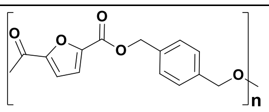	Pyridine TCE	RT	87	2
Poyl (FDCA/MBC)	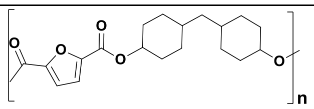	TBT	230	101-142	This work

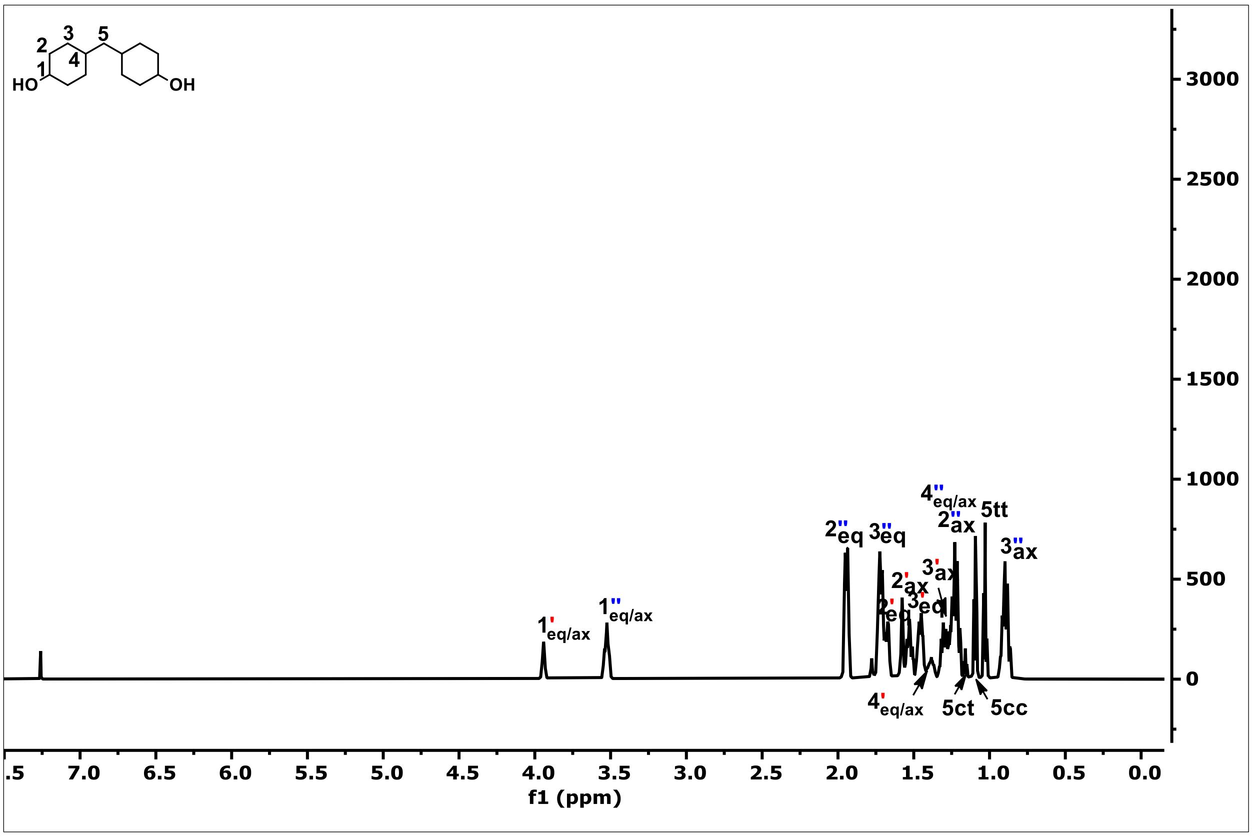

Figure S1 ^{1}H NMR spectrum of $MBC_{cis\text{-}cis,\ cis\text{-}trans,\ trans\text{-}trans}$

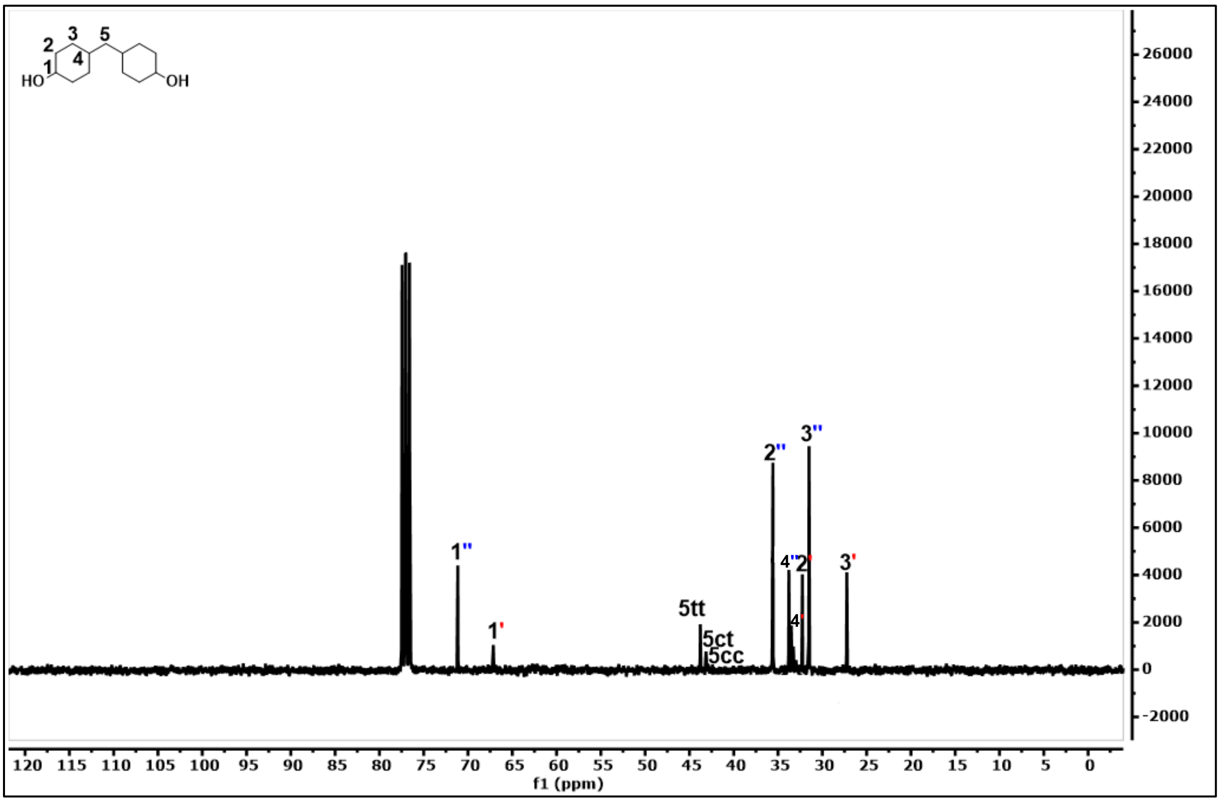

Figure S2 ^{13}C NMR spectrum of $MBC_{cis\text{-}cis,\ cis\text{-}trans,\ trans\text{-}trans}$ (quantitative ^{13}C NMR)

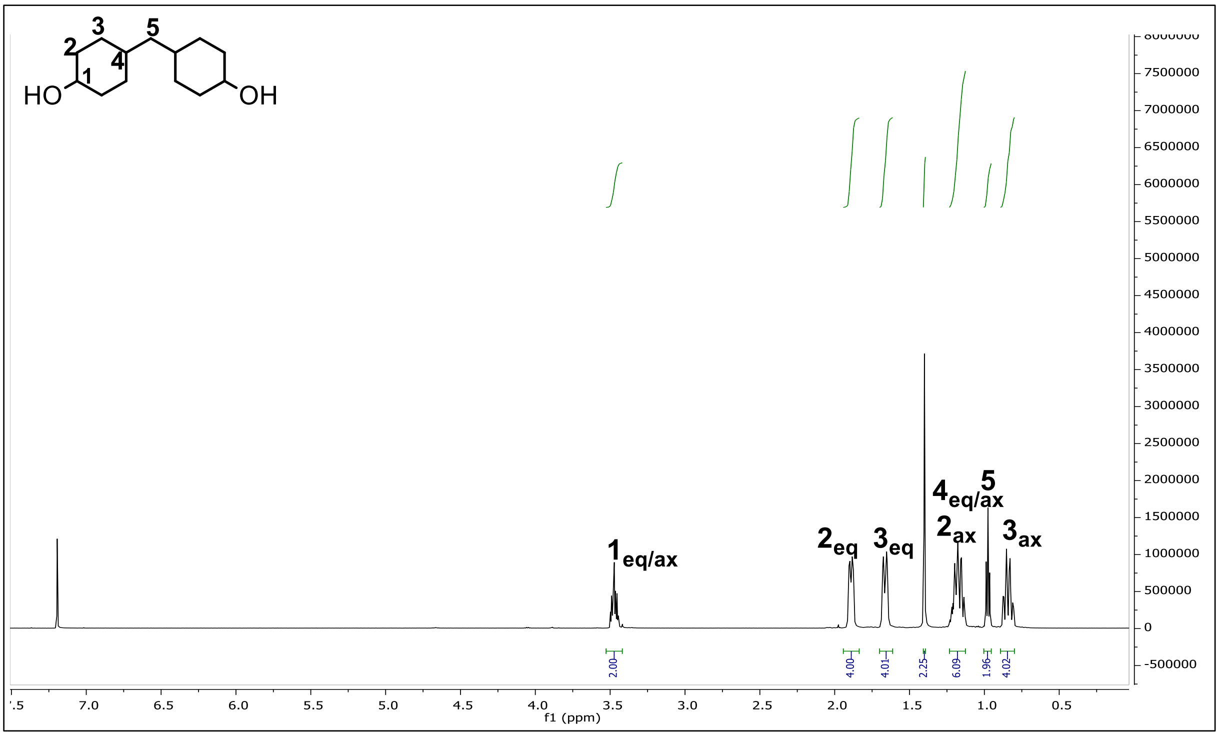

Figure S3 ^{1}H NMR spectrum of $MBC_{trans\text{-}trans}$

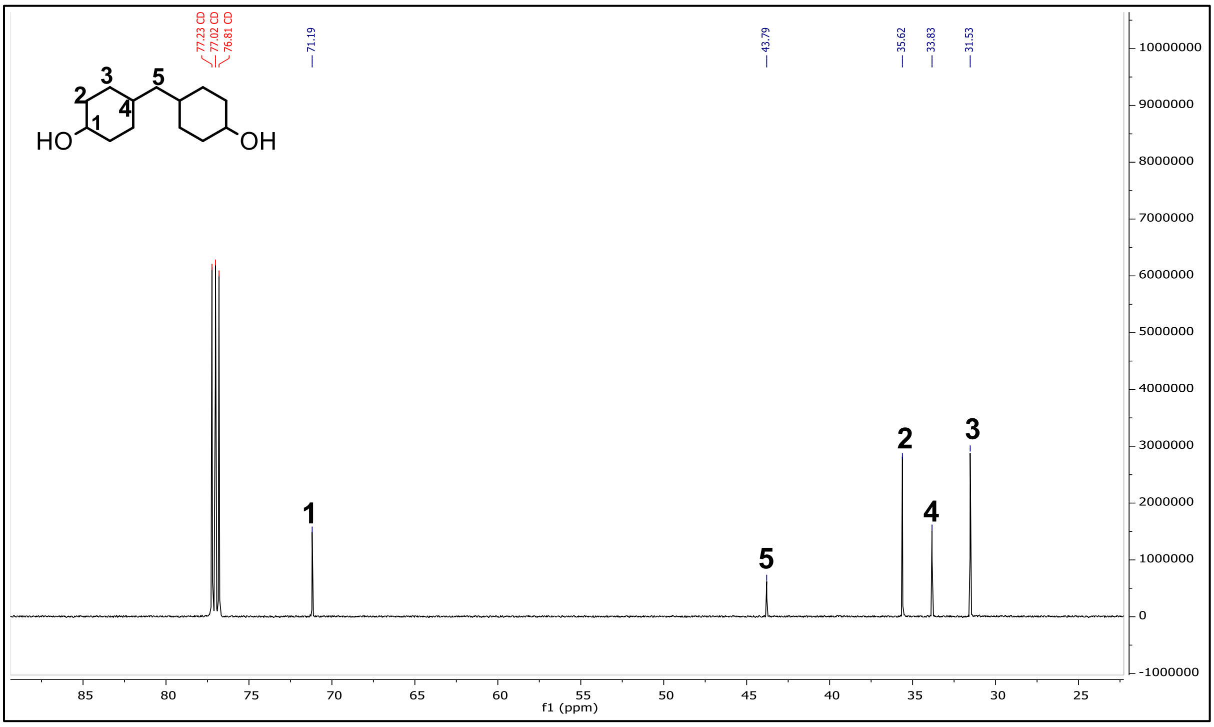

Figure S4 ^{13}C NMR spectrum of $MBC_{trans\text{-}trans}$

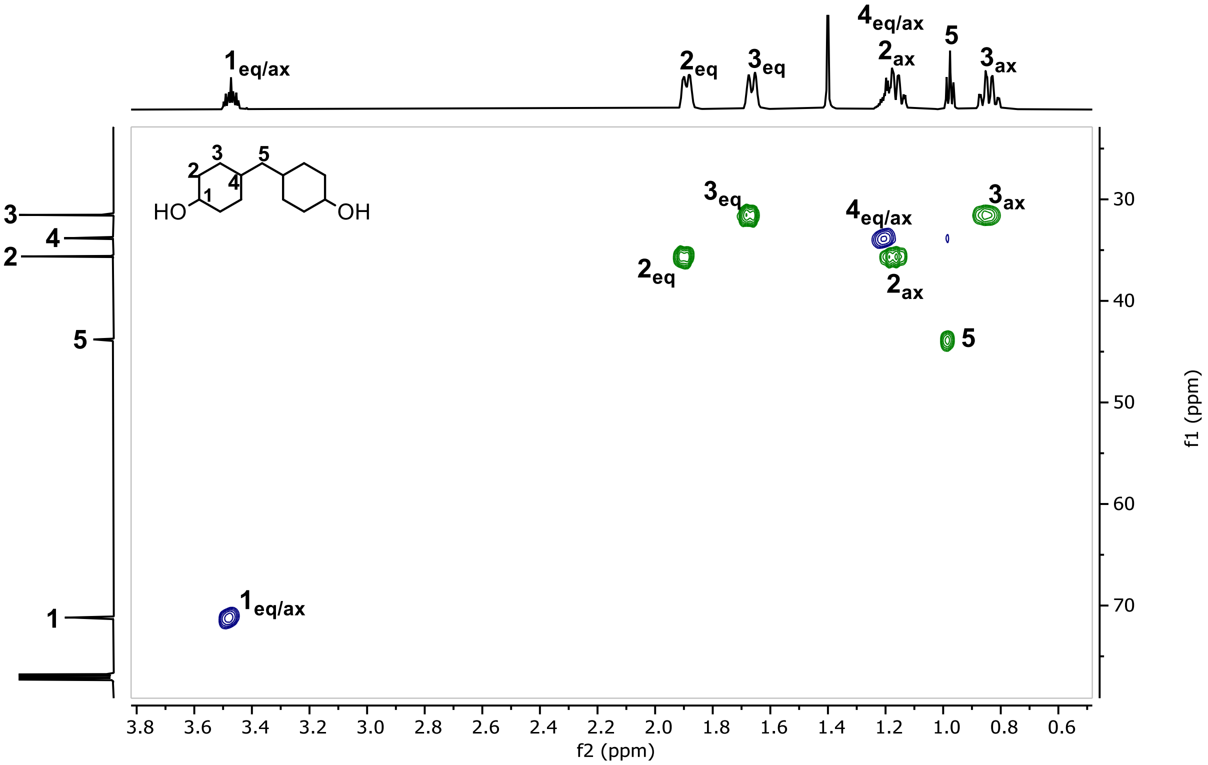

Figure S5 2D HSQC spectrum of $MBC_{trans\text{-}trans}$

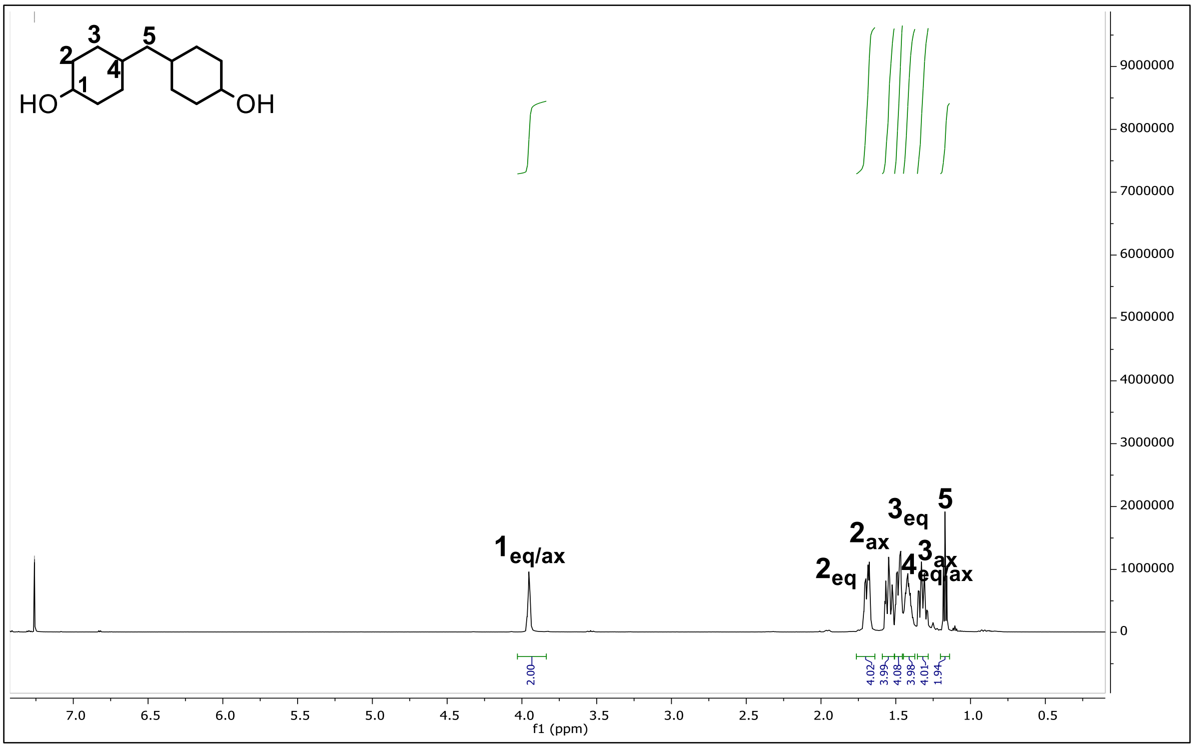

Figure S6 ^{1}H NMR spectrum of $MBC_{cis\text{-}cis}$

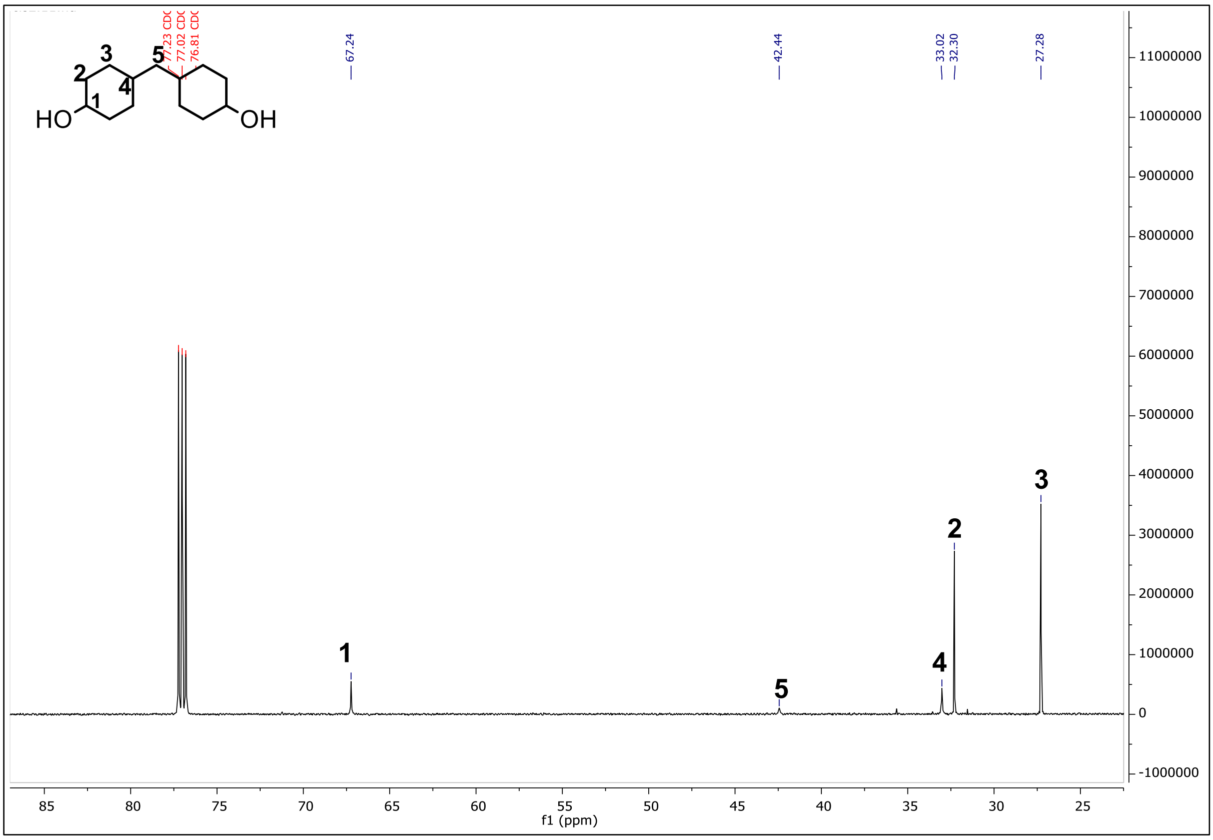

Figure S7 ^{13}C NMR spectrum of $MBC_{cis\text{-}cis}$

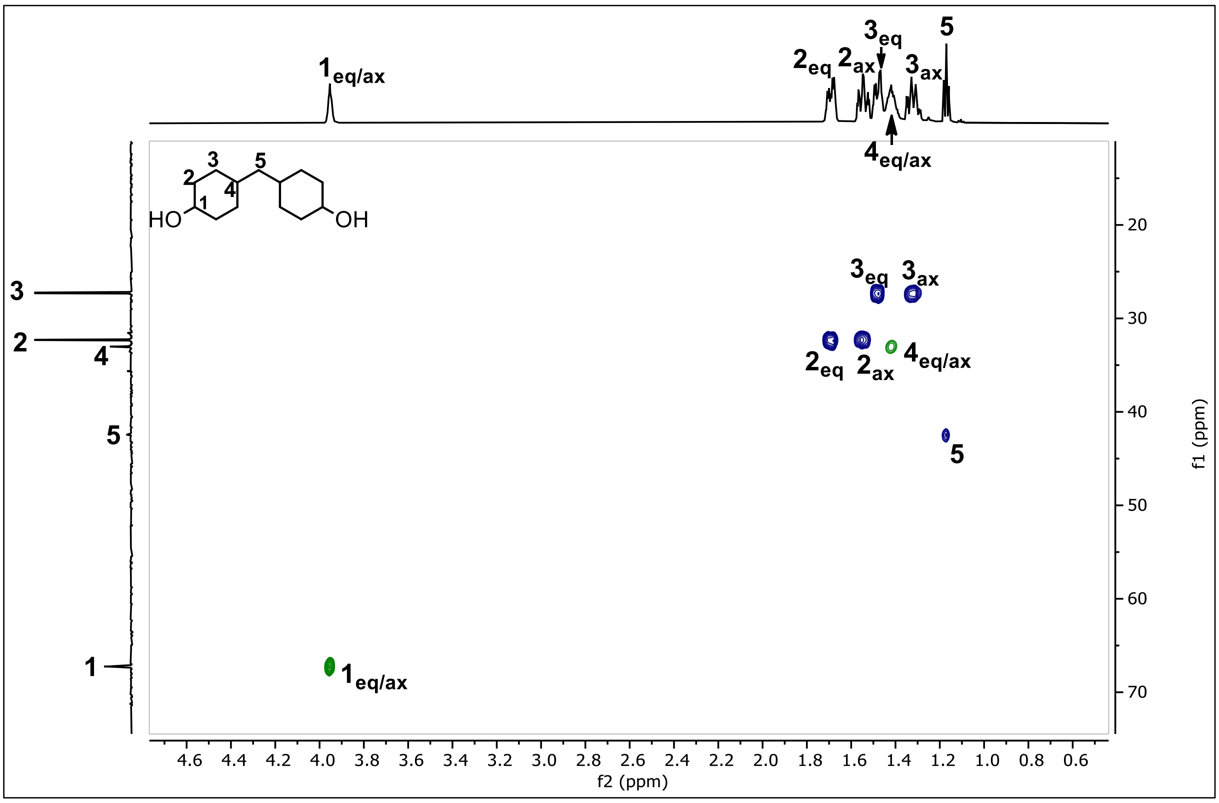

Figure S8 2D HSQC spectrum of $MBC_{cis\text{-}cis}$

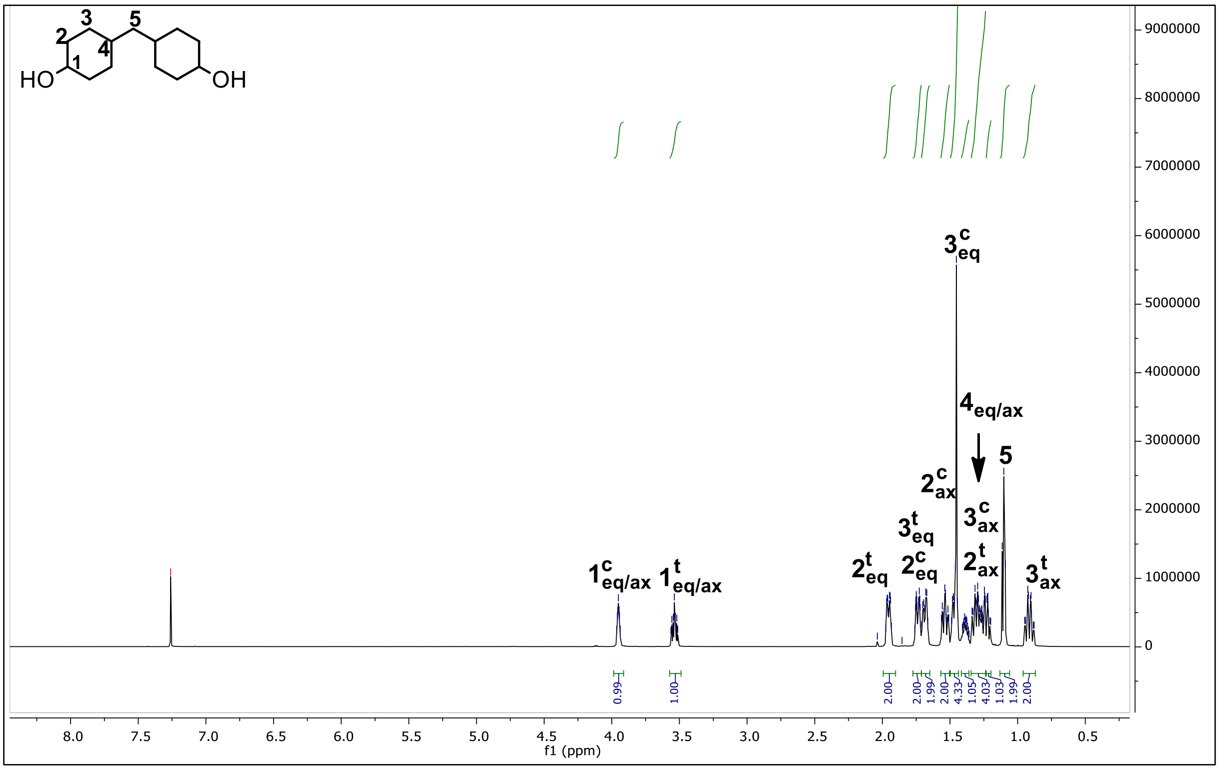

Figure S9 ^{1}H NMR spectrum of $MBC_{cis\text{-}trans}$

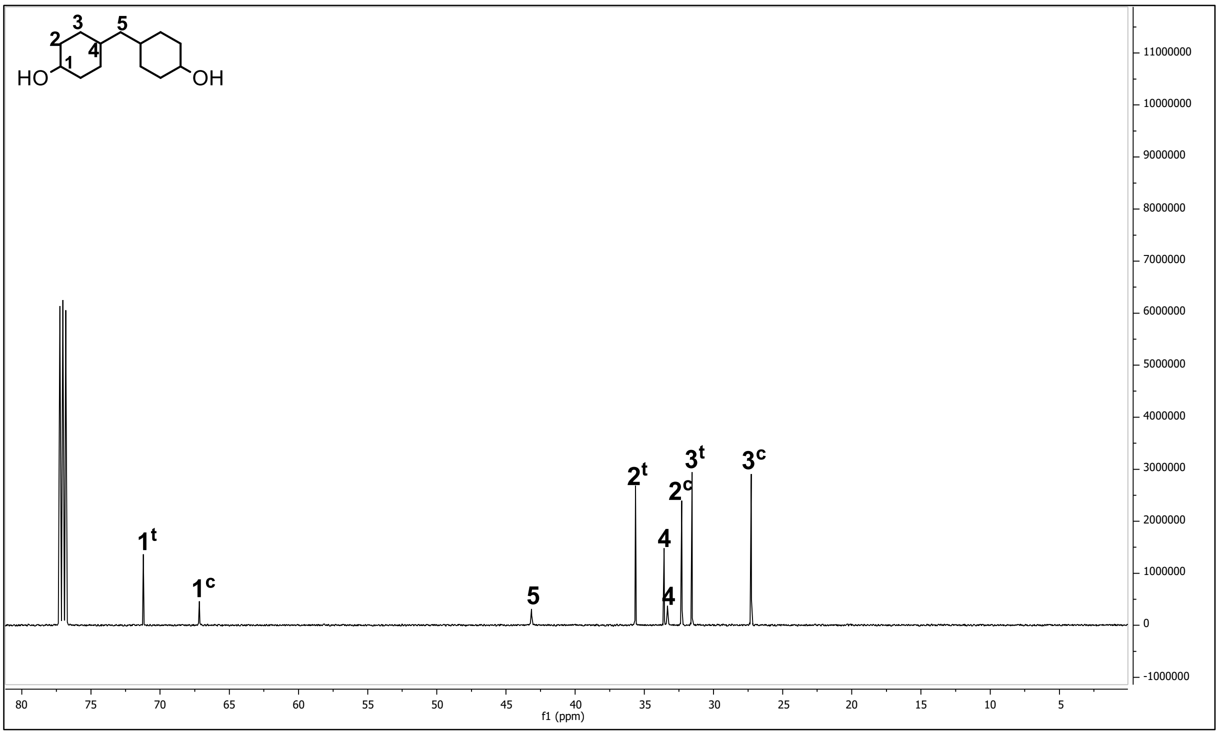

Figure S10 ^{13}C NMR spectrum of $MBC_{cis\text{-}trans}$

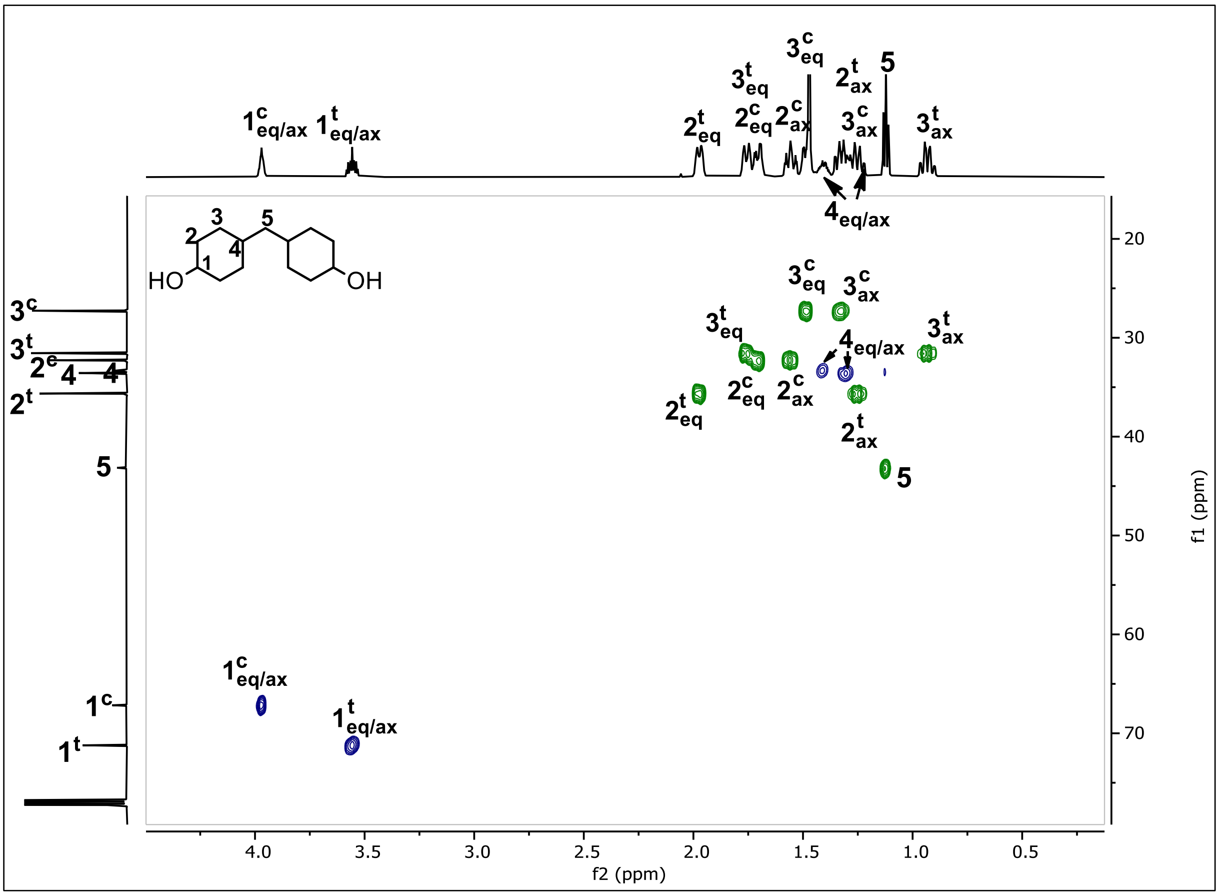

Figure S11 2D HSQC spectrum of $MBC_{cis\text{-}trans}$

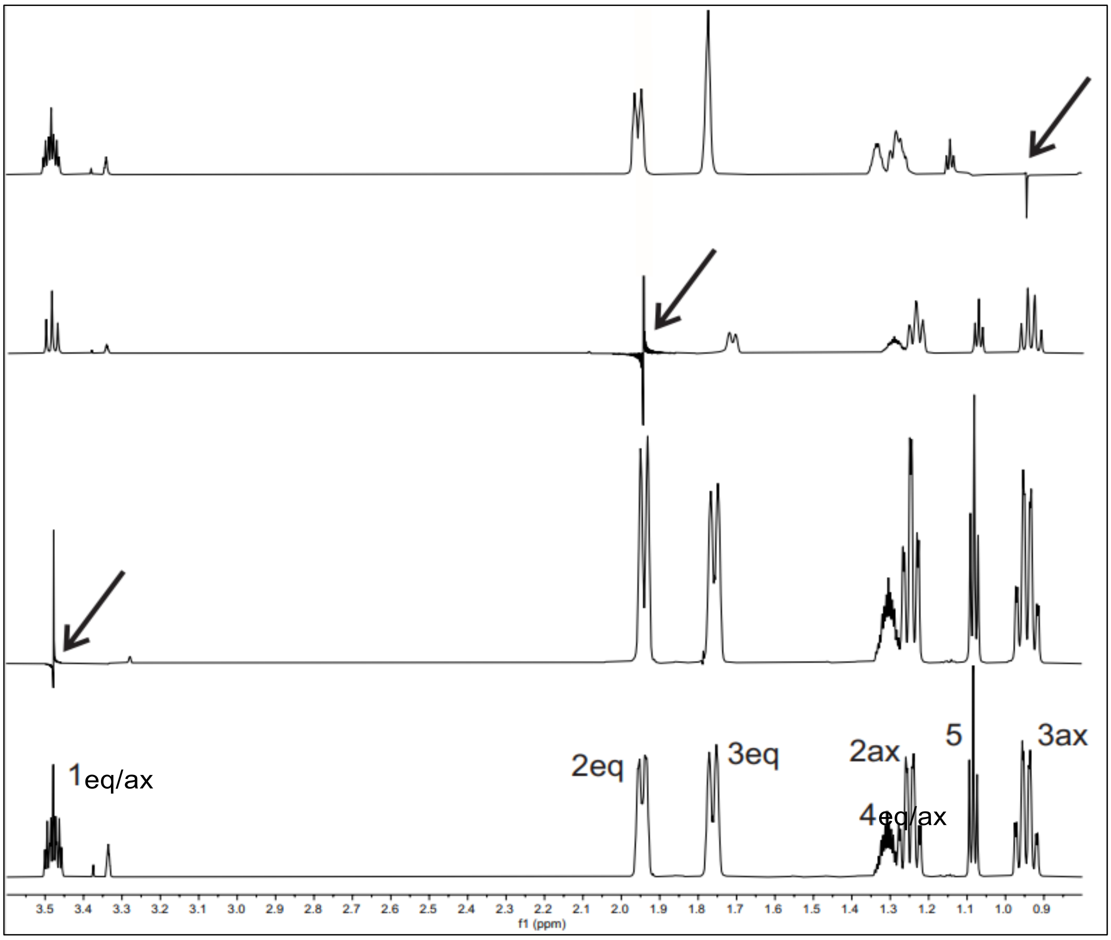

Figure S12 Conventional ^{1}H and various selectively decoupled ^{1}H spectra of $MBC_{trans\text{-}trans}$

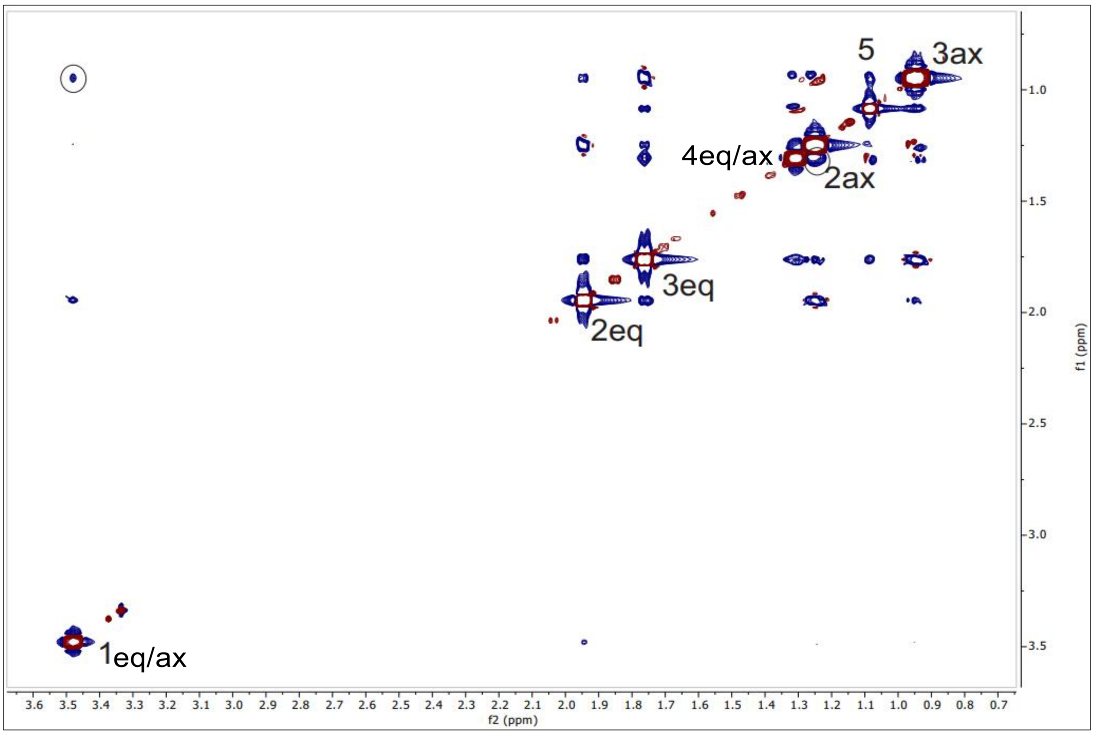

Figure S13 NOESY spectrum of $MBC_{trans\text{-}trans}$ with NOEs indicative of axial Hs circled

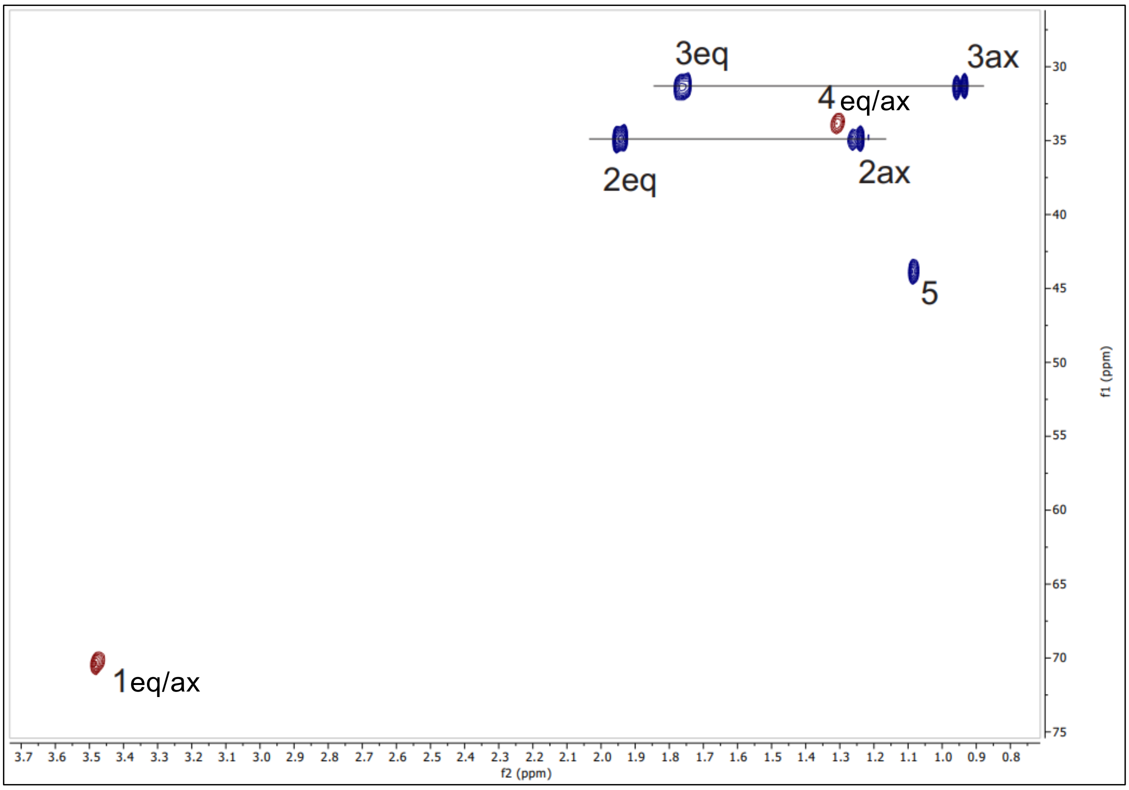

Figure S14 Multiplicity-edited, sensitivity-enhanced ^{1}H-^{13}C HSQC spectra of $MBC_{trans\text{-}trans}$

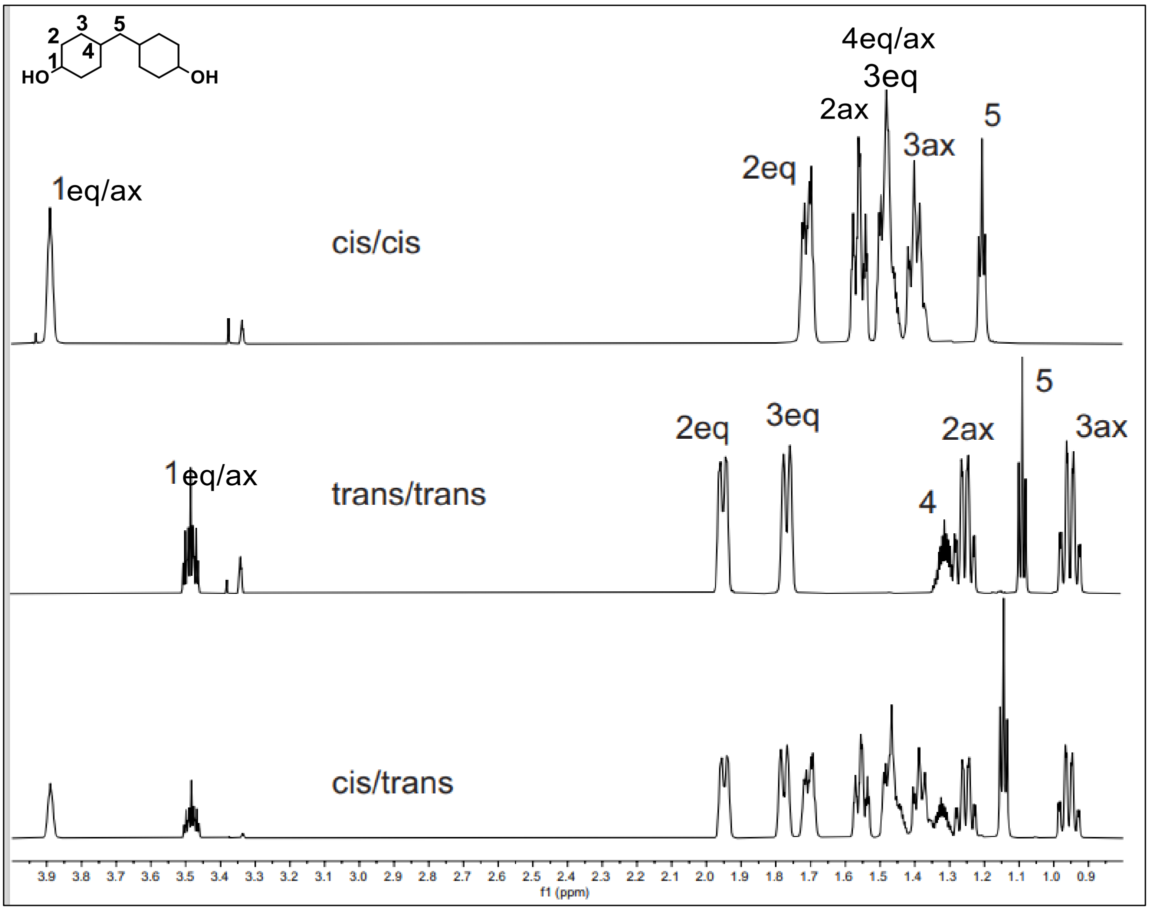

Figure S15 ^{1}H spectra of $MBC_{cis\text{-}cis}$, $MBC_{cis\text{-}trans}$ and $MBC_{trans\text{-}trans}$

The carbon-proton connectivities of the investigated compounds were established using 1D ^{1}H, 2D COSY, ^{1}H-^{13}C HSQC (multiplicity-edited and sensitivity-enhanced for direct CH-CH connectivities) as well as 2D ^{1}H-^{13}C HMBC spectra. For the determination of the configuration on the 6-membered rings, e.g. equatorial and axial orientations of the hydrogens on the ring, information about ^{1}H-^{1}H NOEs and the size of 3-bound ^{1}H-^{1}H scalar coupling constants is used. NOEs between protons separated by 4 bonds can only be found between axial protons on the same side of the ring (e.g. position H1 with H3 and H2 with H4). Semi-quantitative scalar coupling information is used to corroborate the axial orientations since large coupling constants (^{3}J>8 Hz) can only be found between neighboring axial protons, while all other coupling constants Heq-Heq and Heq-Hax are significantly smaller (^{3}J<5 Hz). The configurations of the investigated compounds are shown in Figure S16-19. As an example, for its determination, the individual spectra of the $MBC_{trans-trans}$ isomer are shown in Figure S18-21. In the 2D NOESY spectrum NOEs are found between H1-H3ax and H2ax-H4 (indicated by circles in Figure S17). Semi-quantative, relative J-values can be extracted from Figure S16, which shows the conventional ^{1}H spectrum together with selectively decoupled ^{1}H spectra, where the frequency of decoupling is indicated by an arrow. Decoupling of proton 1 leads to removal of only small couplings on proton 2eq, but a large coupling on proton 2ax (reduction of the quartet to a triplet). This indicated to an axial arrangement of both proton 1 and 2ax. Decoupling of proton 2eq removes only small couplings on protons 1, 3eq and 3ax, but a large geminal coupling to 2ax, indicative of its equatorial orientation. Finally, decoupling of 3ax results in a removal of the large geminal coupling to 3eq and a significant narrowing (removal of a large coupling) to proton 4. Equivalent arguments were used to determine the conformation of the $MBC_{cis-cis}$ isomer. The cis-trans isomer finally shows an exact combination of protons 1 to 4 in the trans-trans as well as cis-cis isomer (Figure S19), with only one signal for protons 5, which is located halfway between the corresponding signal in the $MBC_{trans-trans}$ and $MBC_{cis-trans}$ isomers. This results from an averaging of the chemical shift anisotropy on proton 5 on the cyclohexane ring in the cis and trans orientation. In addition, the proton 1 and 4 on the $MBC_{trans-trans}$ ring could be two different conformations, both axial or equatorial.

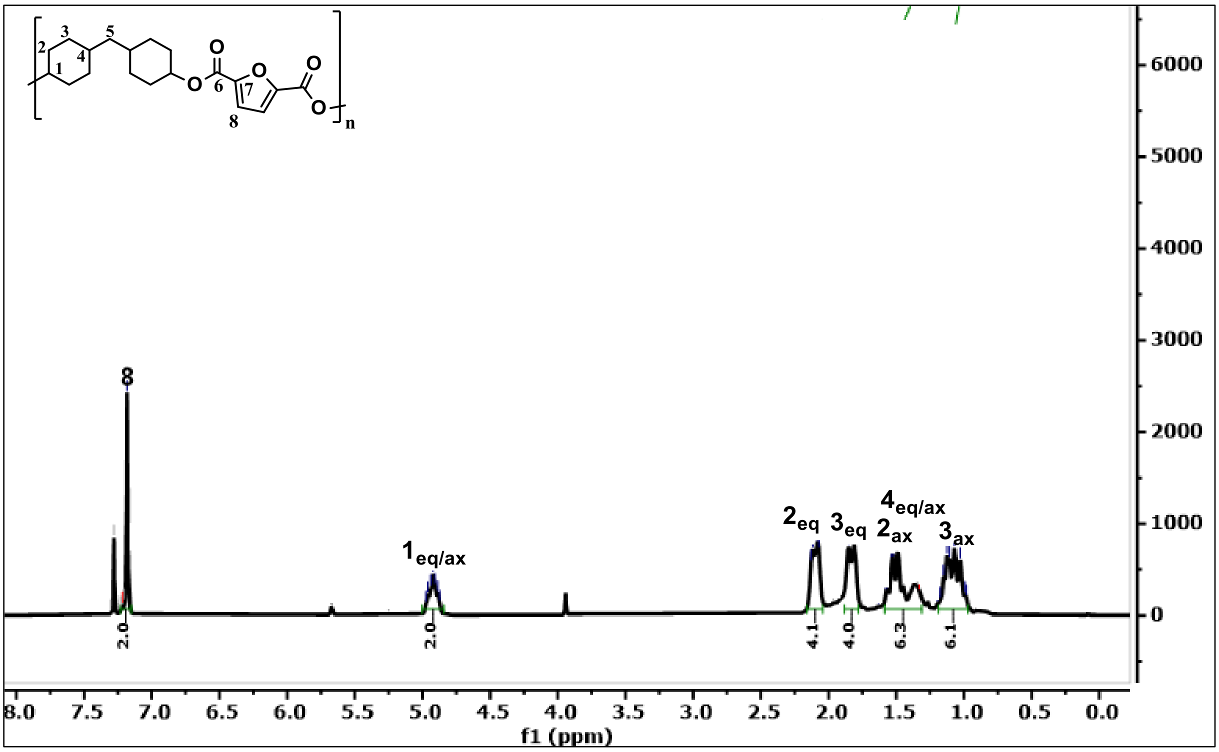

Figure S16 ^{1}H NMR spectrum of poly(MBC$_{trans\text{-}trans}$/FDCA)

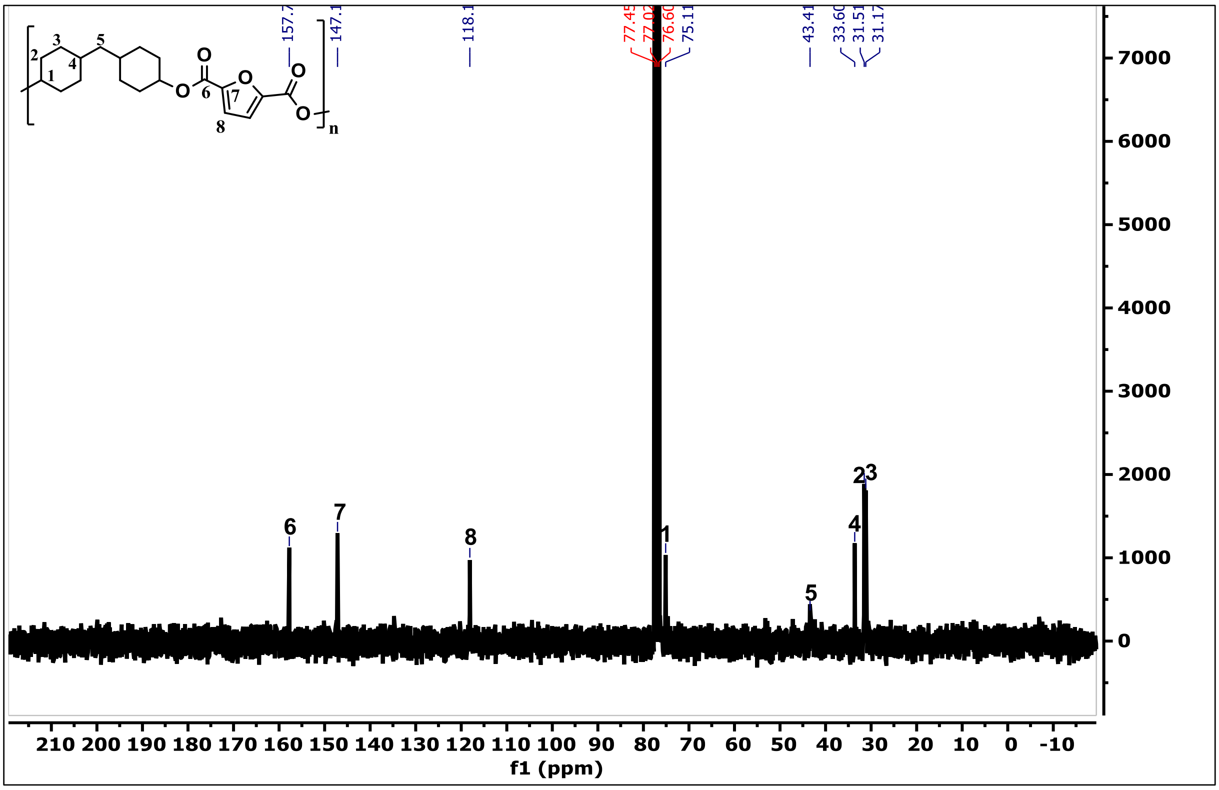

Figure S17 ^{13}C NMR spectrum of poly(MBC$_{trans\text{-}trans}$/FDCA)

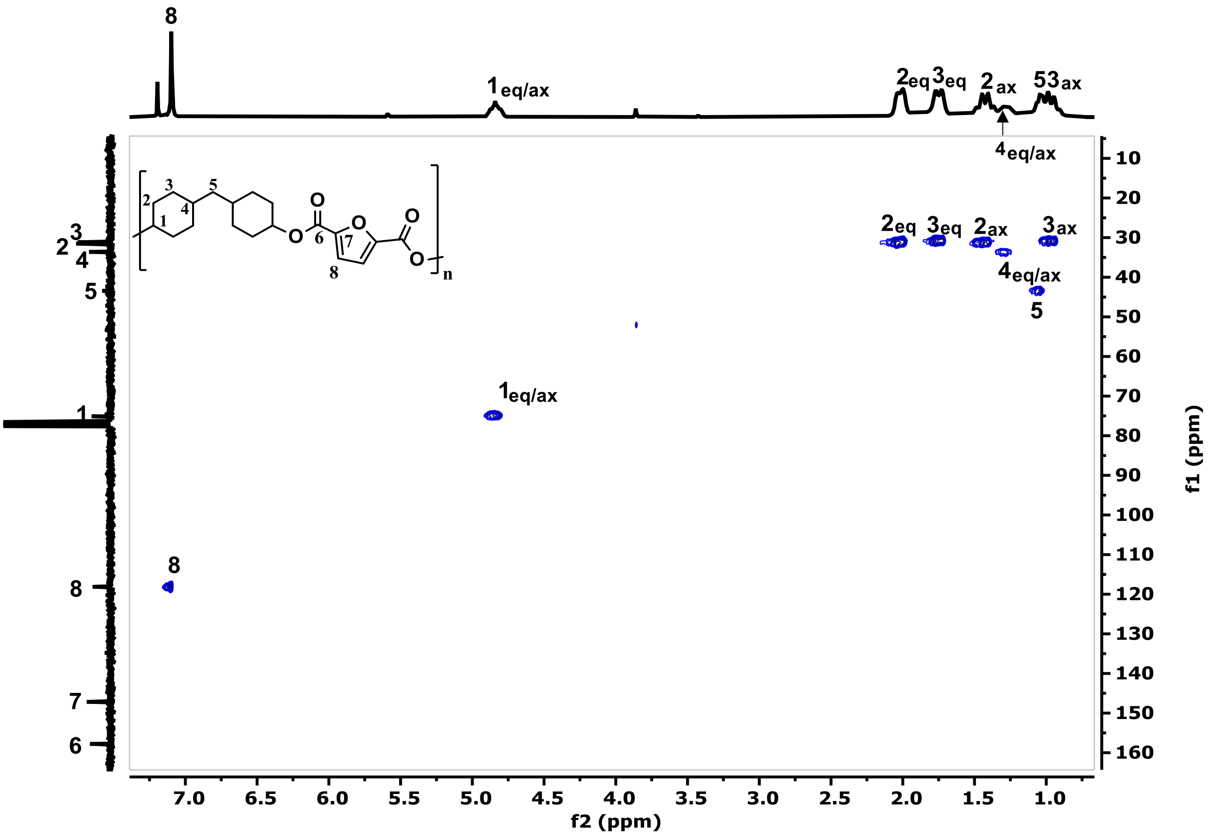

Figure S18 2D HSQC spectrum of poly(MBC$_{trans\text{-}trans}$/FDCA)

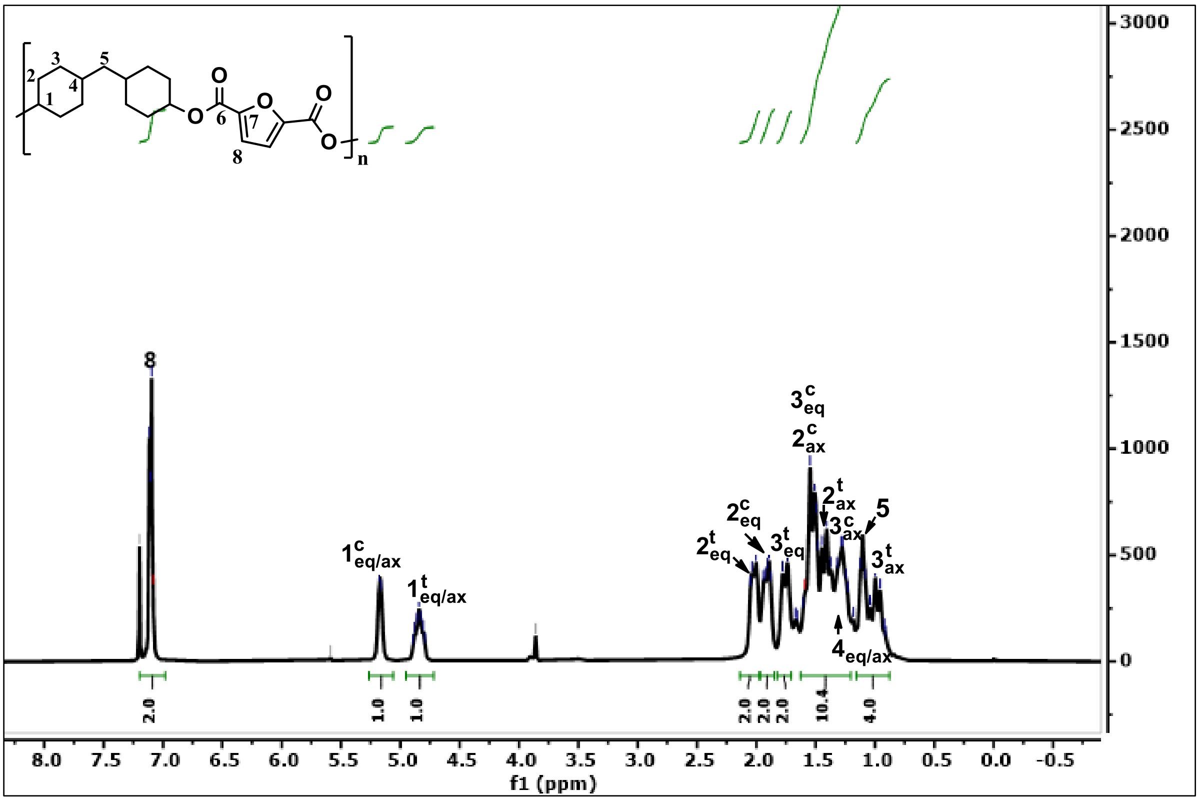

Figure S19 ^{1}H NMR spectrum of poly(MBC$_{cis\text{-}trans}$/FDCA)

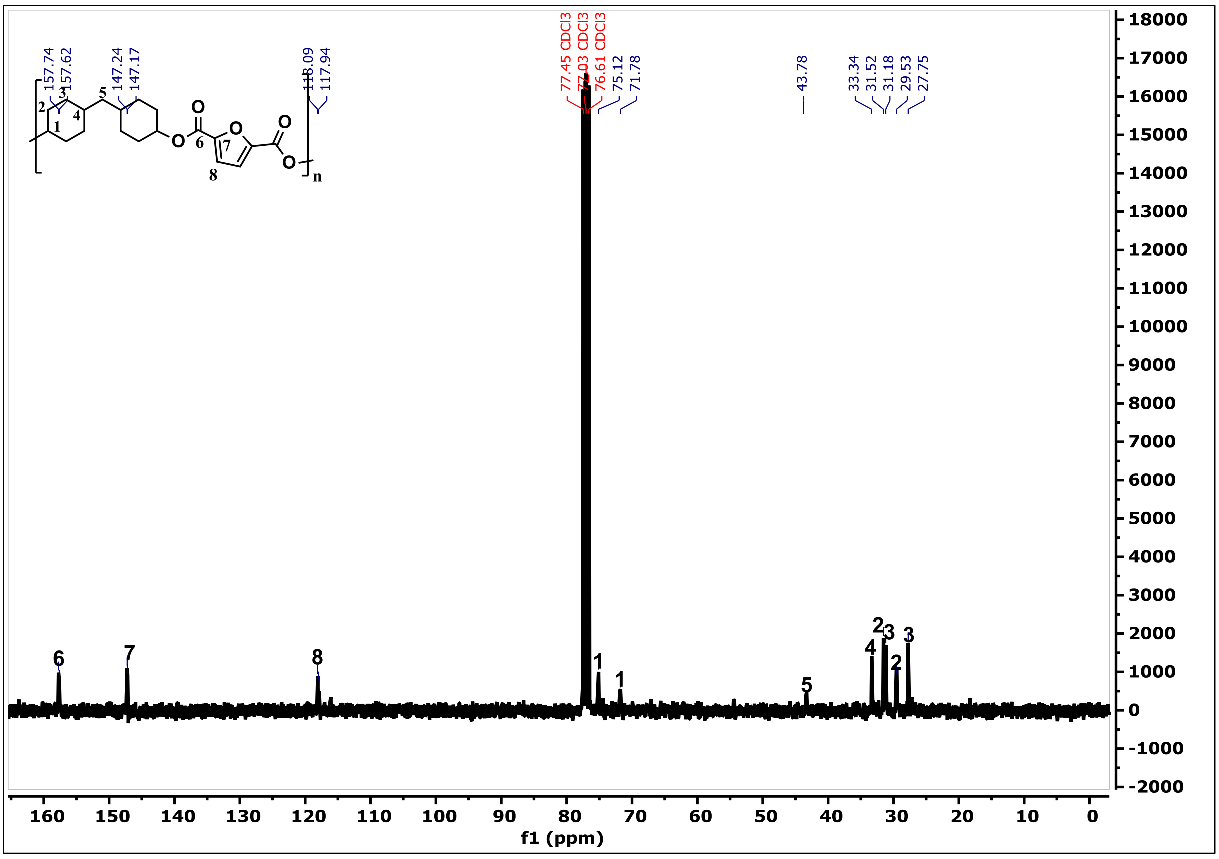

Figure S20 ^{13}C NMR spectrum of poly(MBC$_{\text{cis-trans}}$/FDCA)

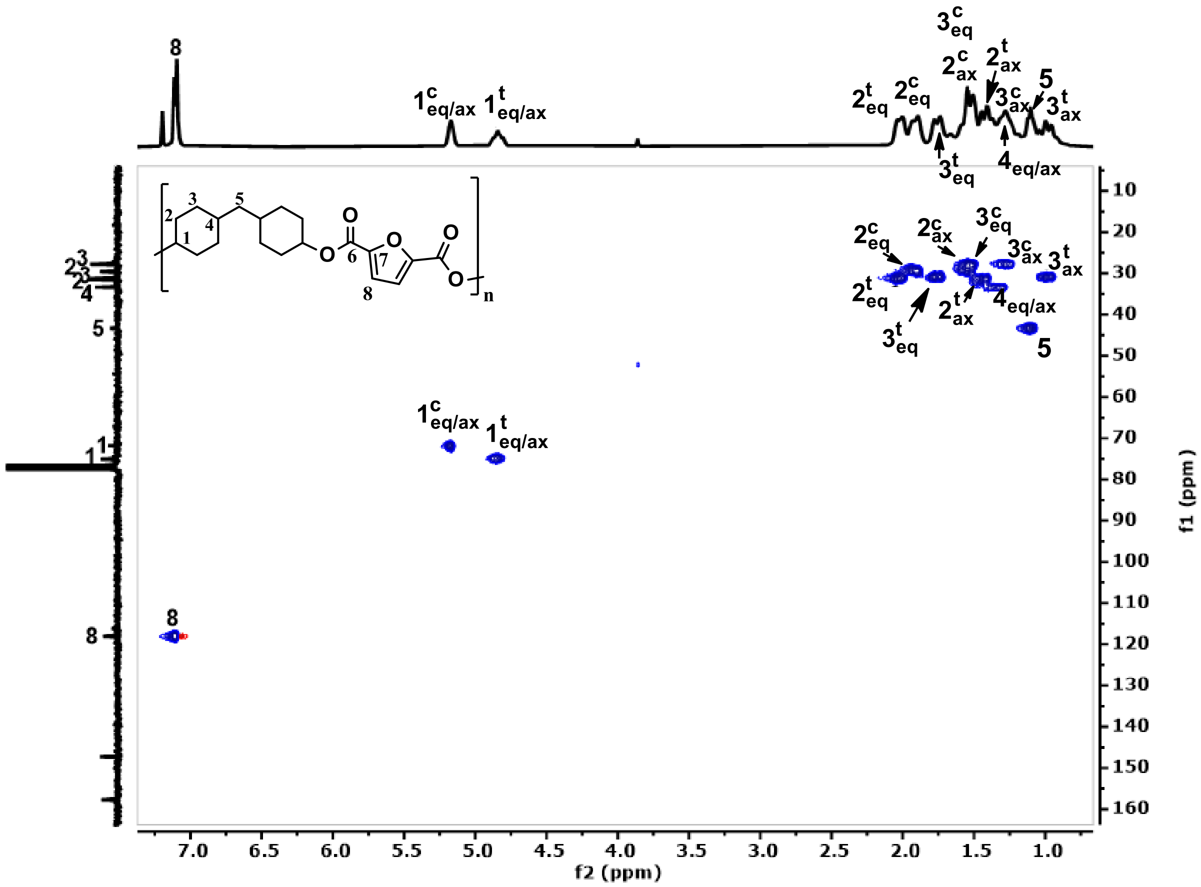

Figure S21 2D HSQC spectrum of poly(MBC$_{\text{cis-trans}}$/FDCA)

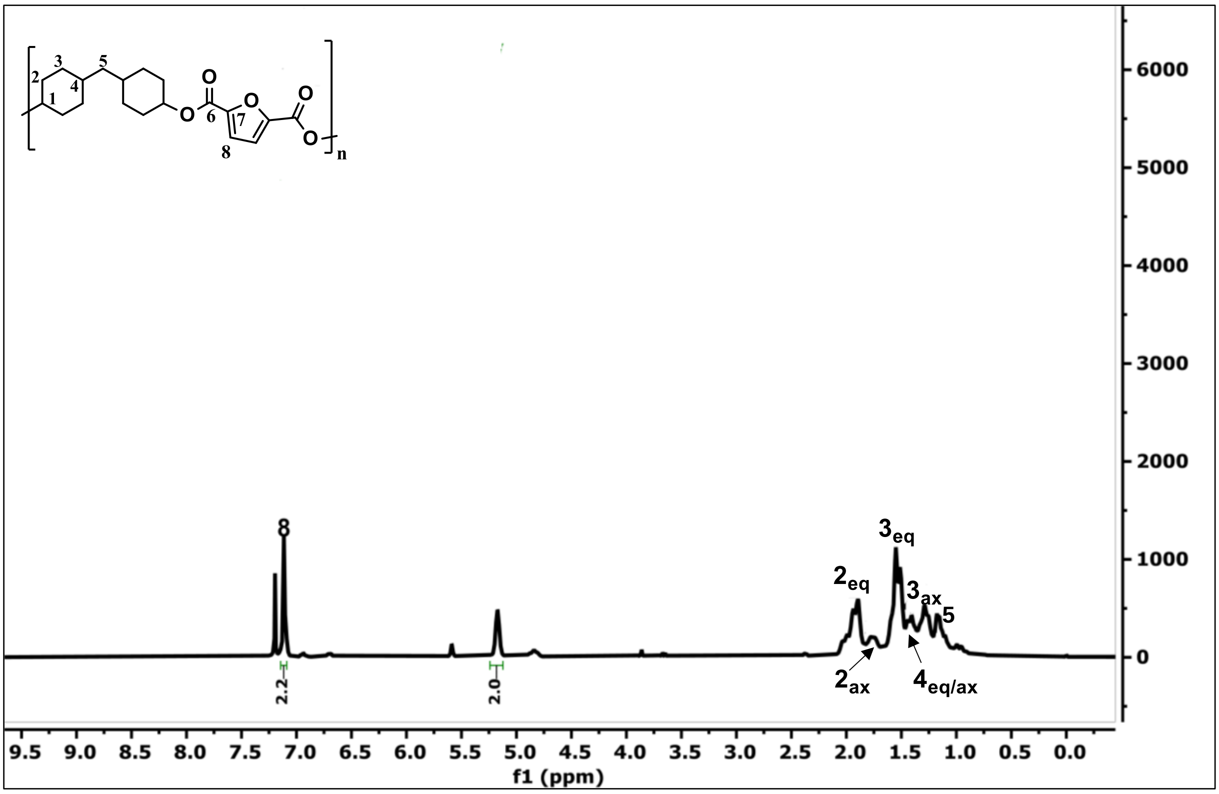

Figure S22 ^{1}H NMR spectrum of poly(MBC$_{cis\text{-}cis}$/FDCA)

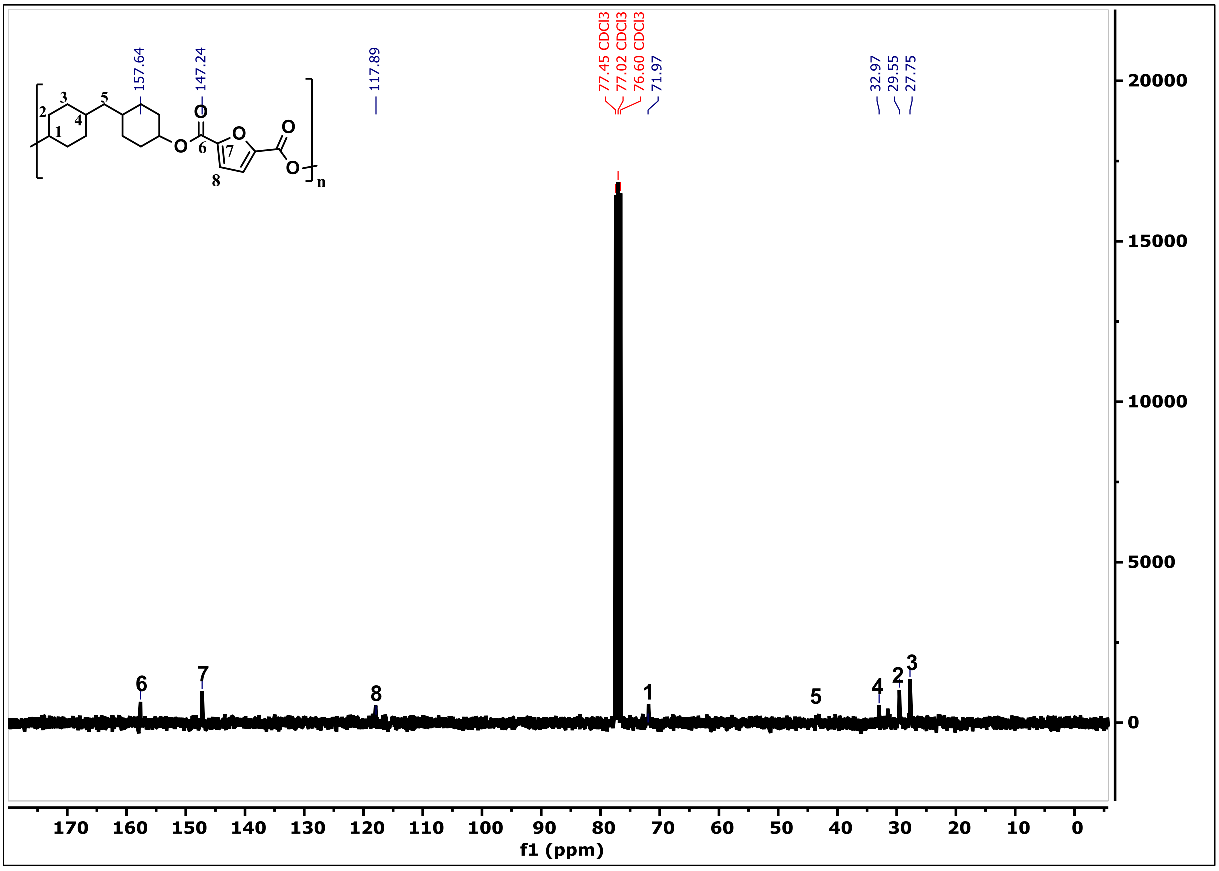

Figure S23 ^{13}C NMR spectrum of poly(MBC$_{cis\text{-}cis}$/FDCA)

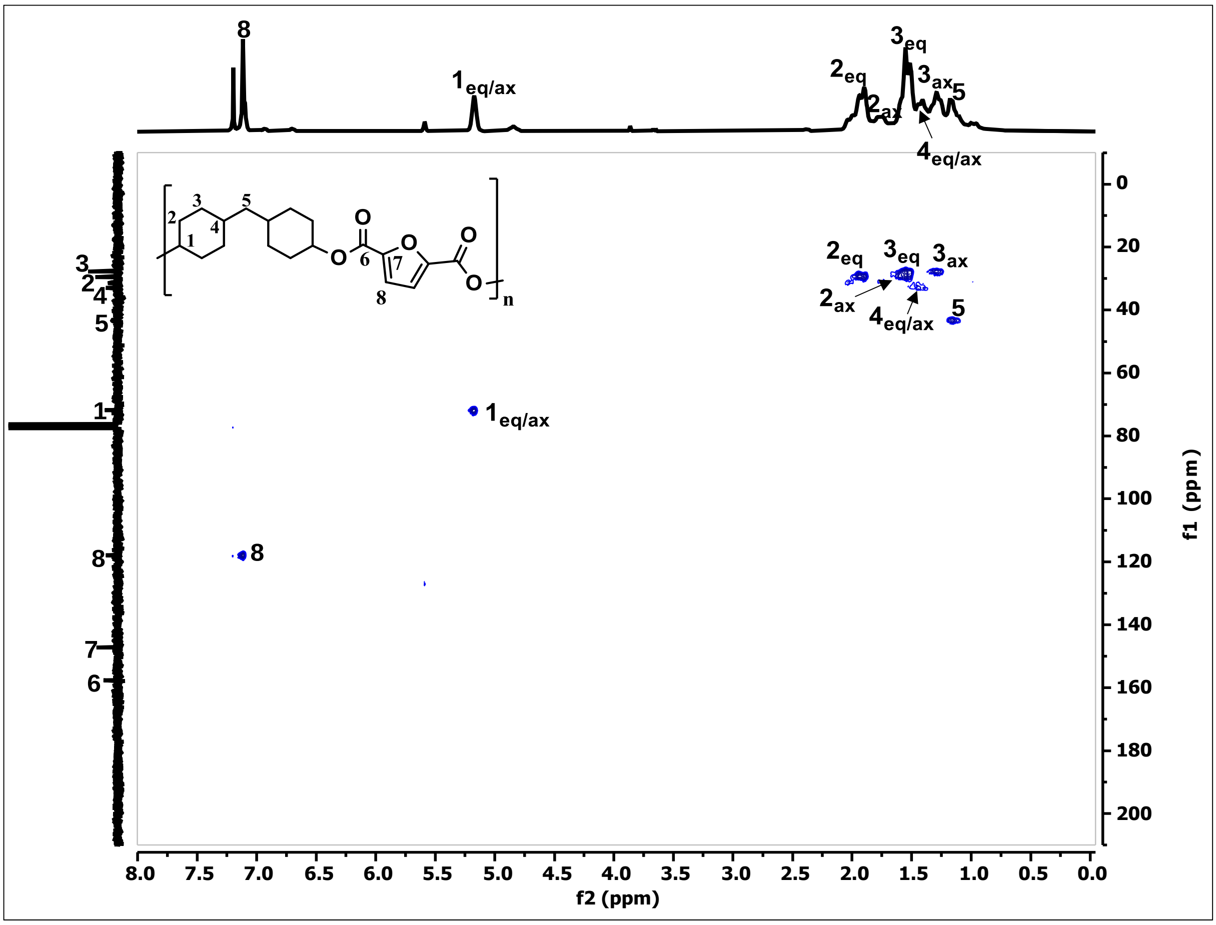

Figure S24 2D HSQC spectrum of poly(MBC$_{cis\text{-}cis}$/FDCA)

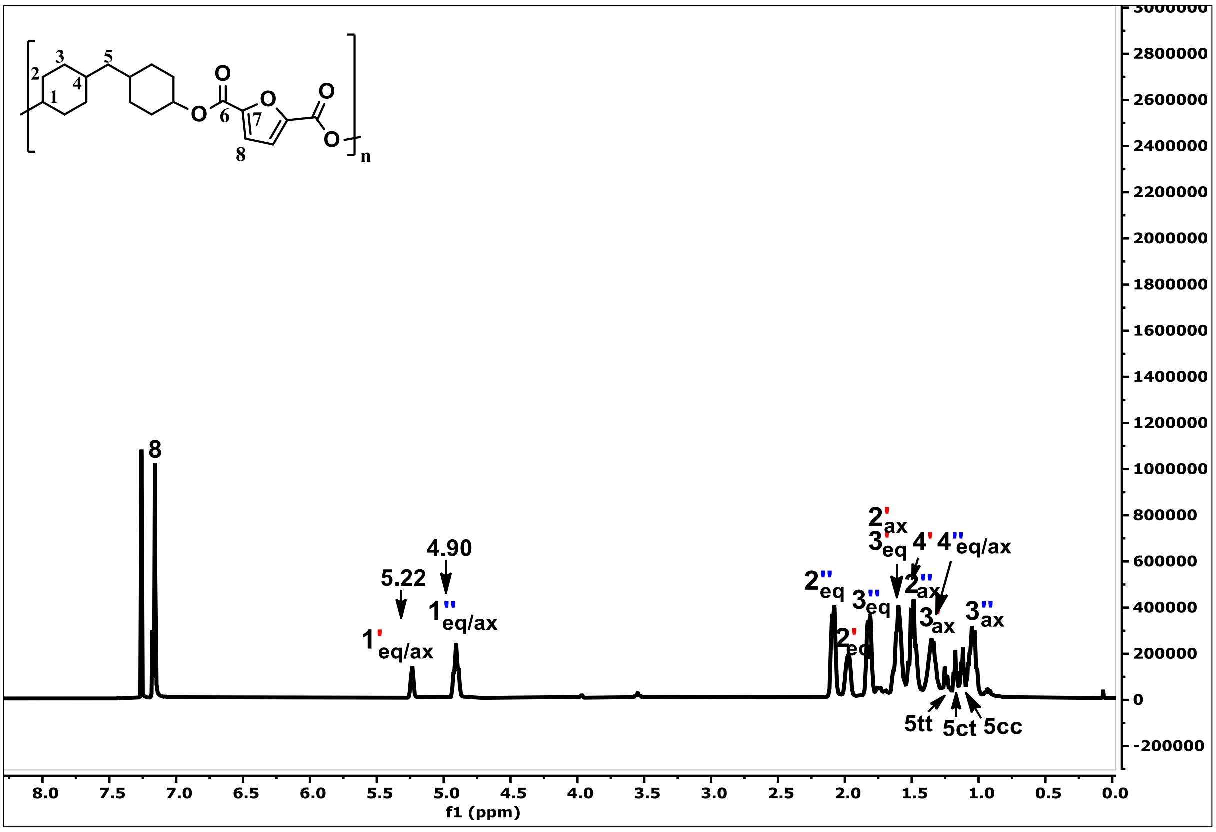

Figure S25 ^{1}H NMR spectrum of poly(MBC/FDCA)

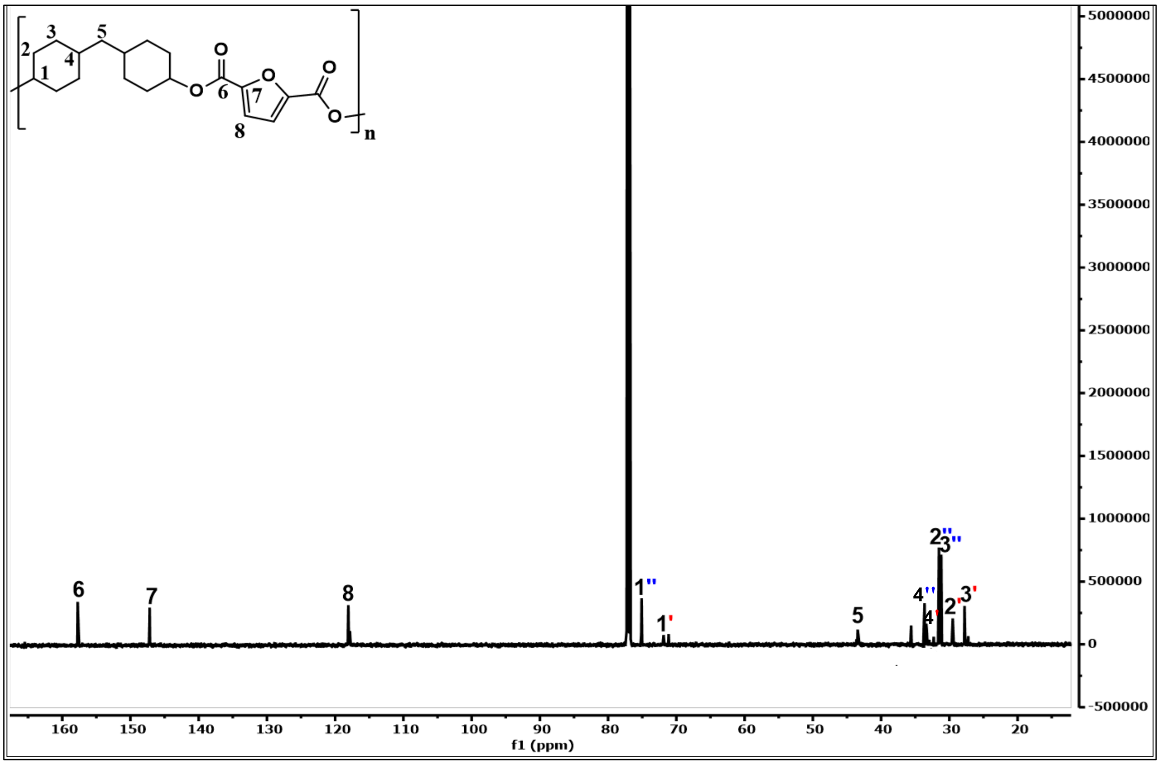

Figure S26 ^{13}C NMR spectrum of poly(MBC/FDCA)

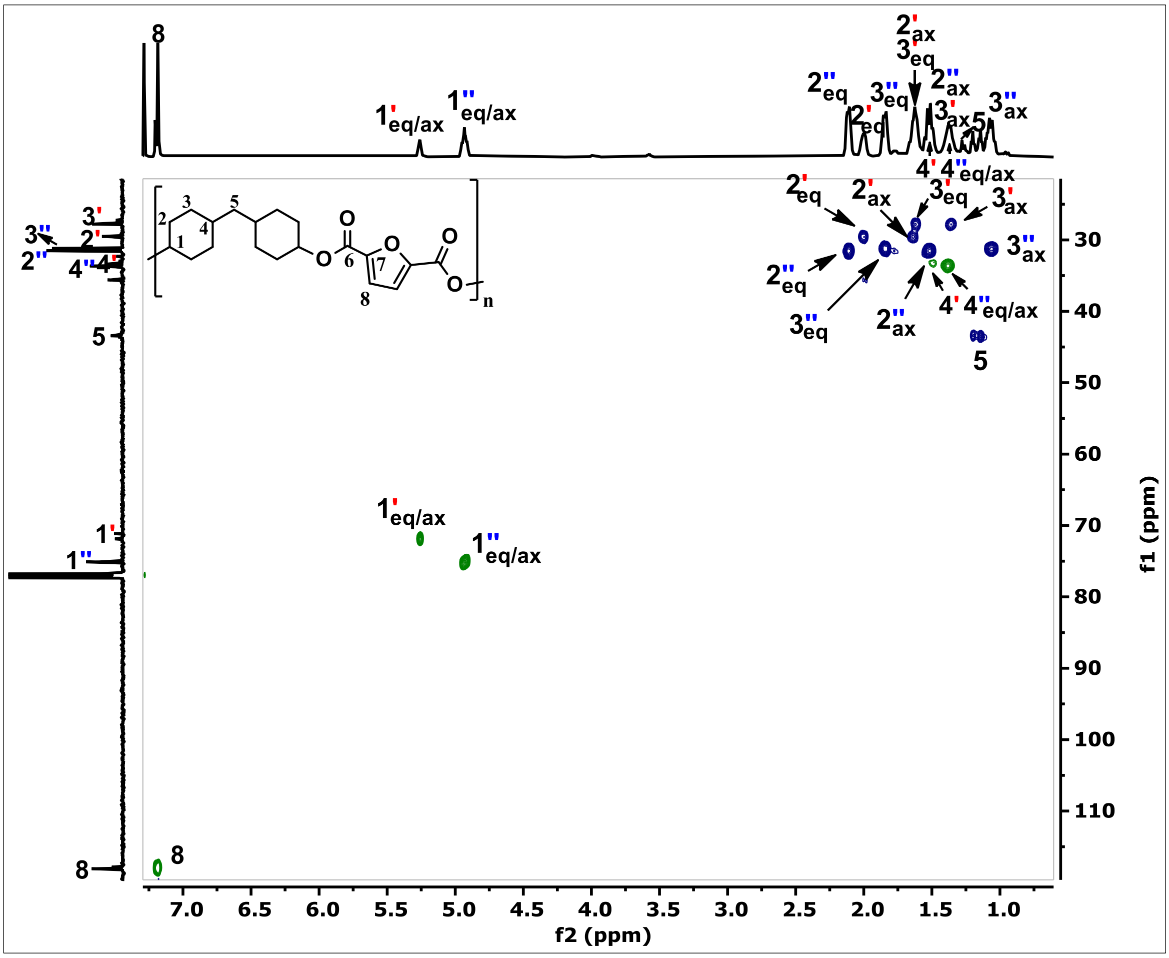

Figure S27 2D HSQC spectrum of poly(MBC/FDCA)

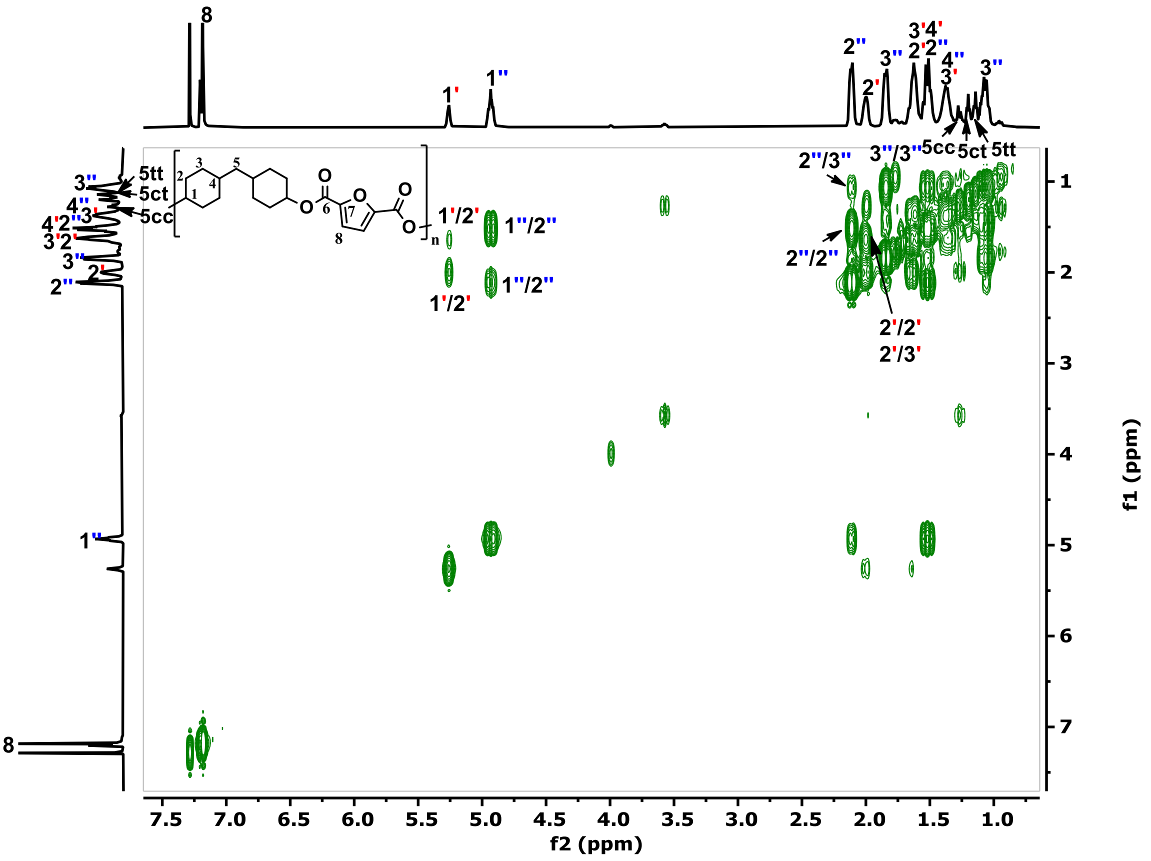

Figure S28 2D COSY spectrum of poly(MBC/FDCA)

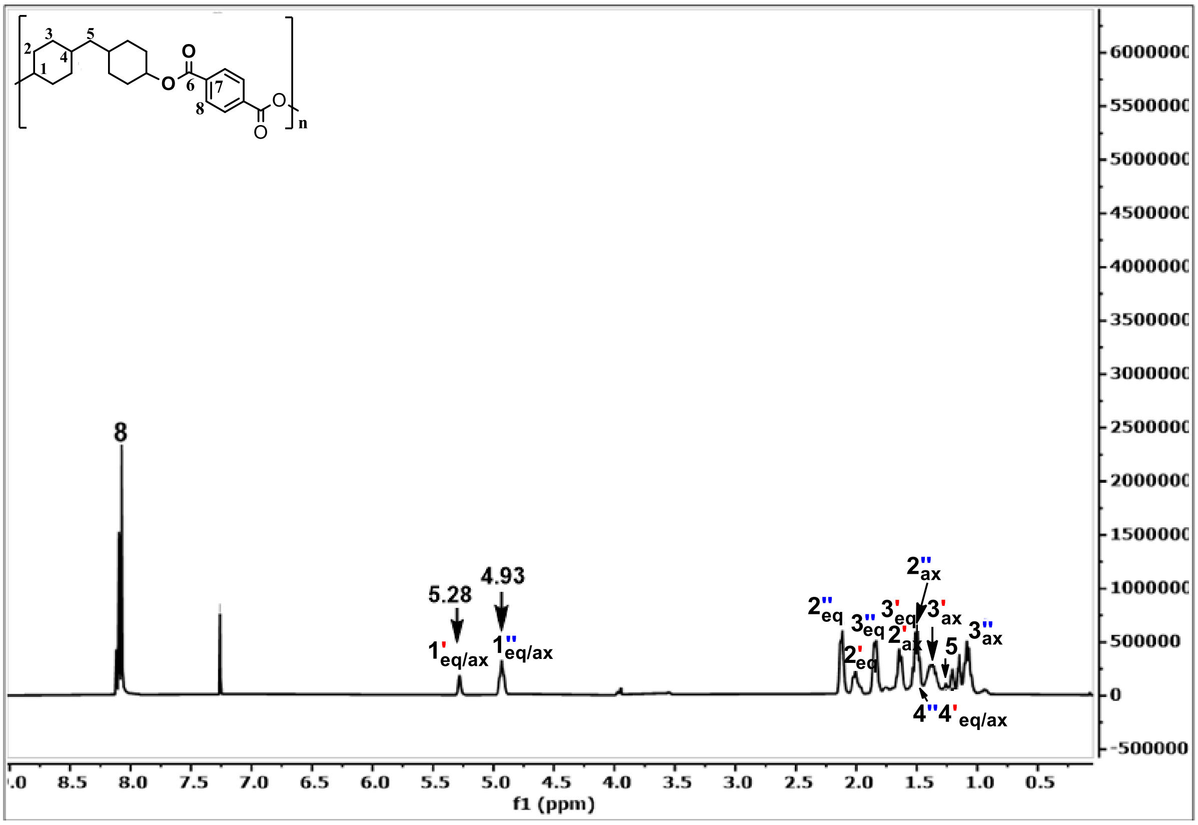

Figure S29 ^{1}H NMR spectrum of poly(MBC/TPA)

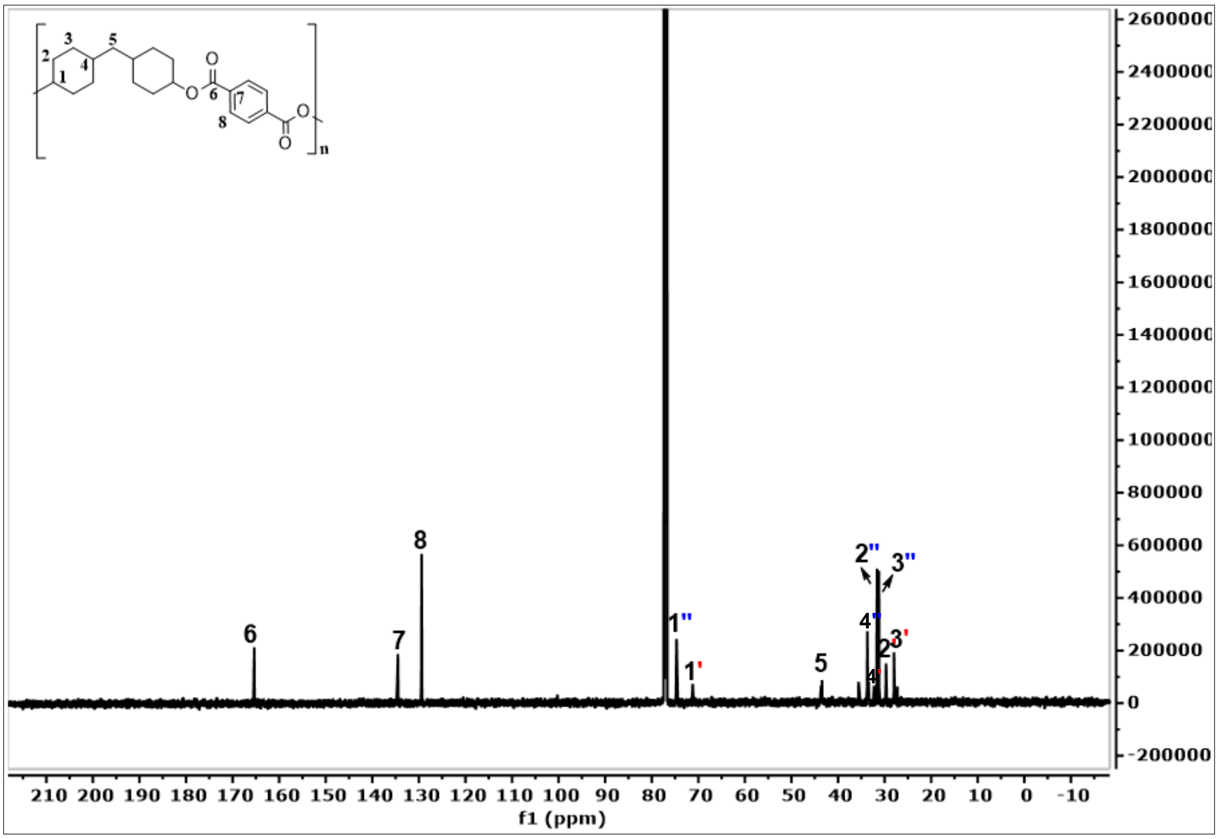

Figure S30 ^{13}C NMR spectrum of poly(MBC/TPA)

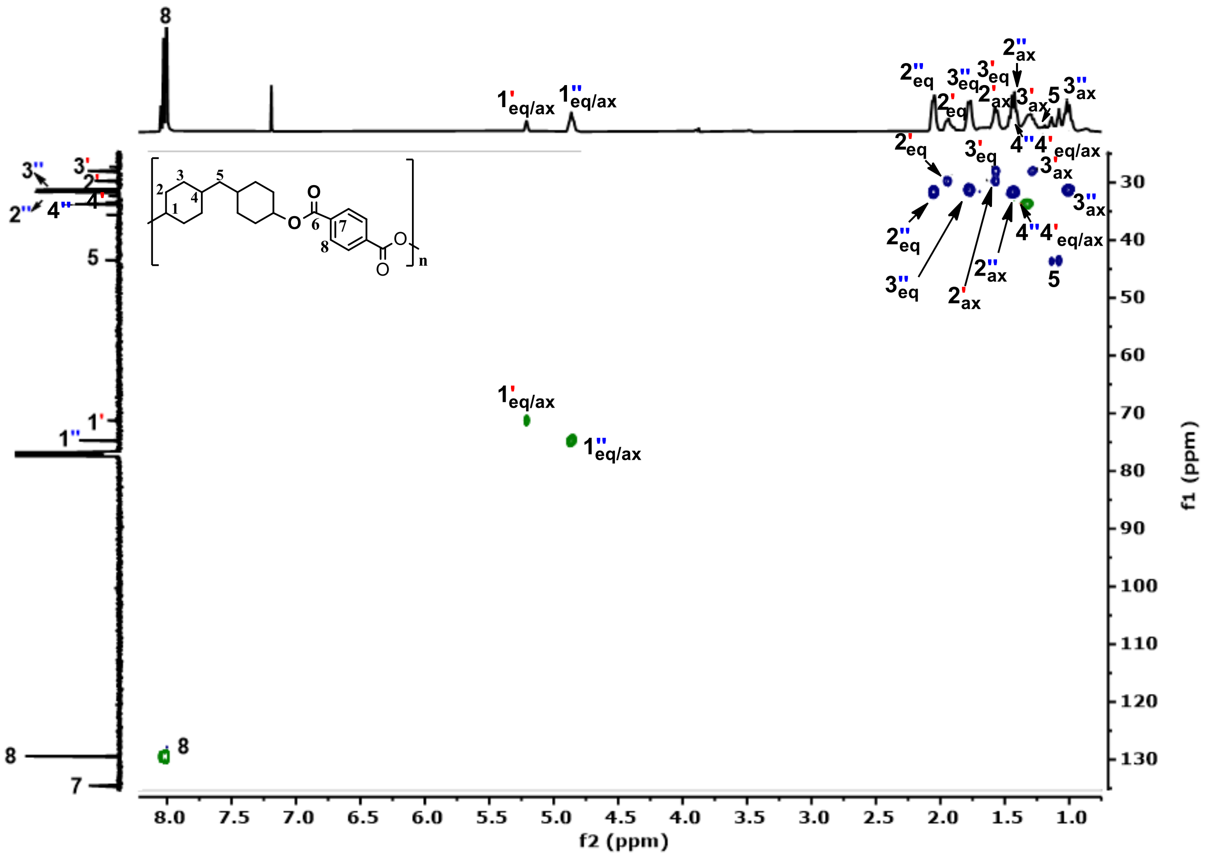

Figure S31 2D HSQC spectrum of poly(MBC/TPA)

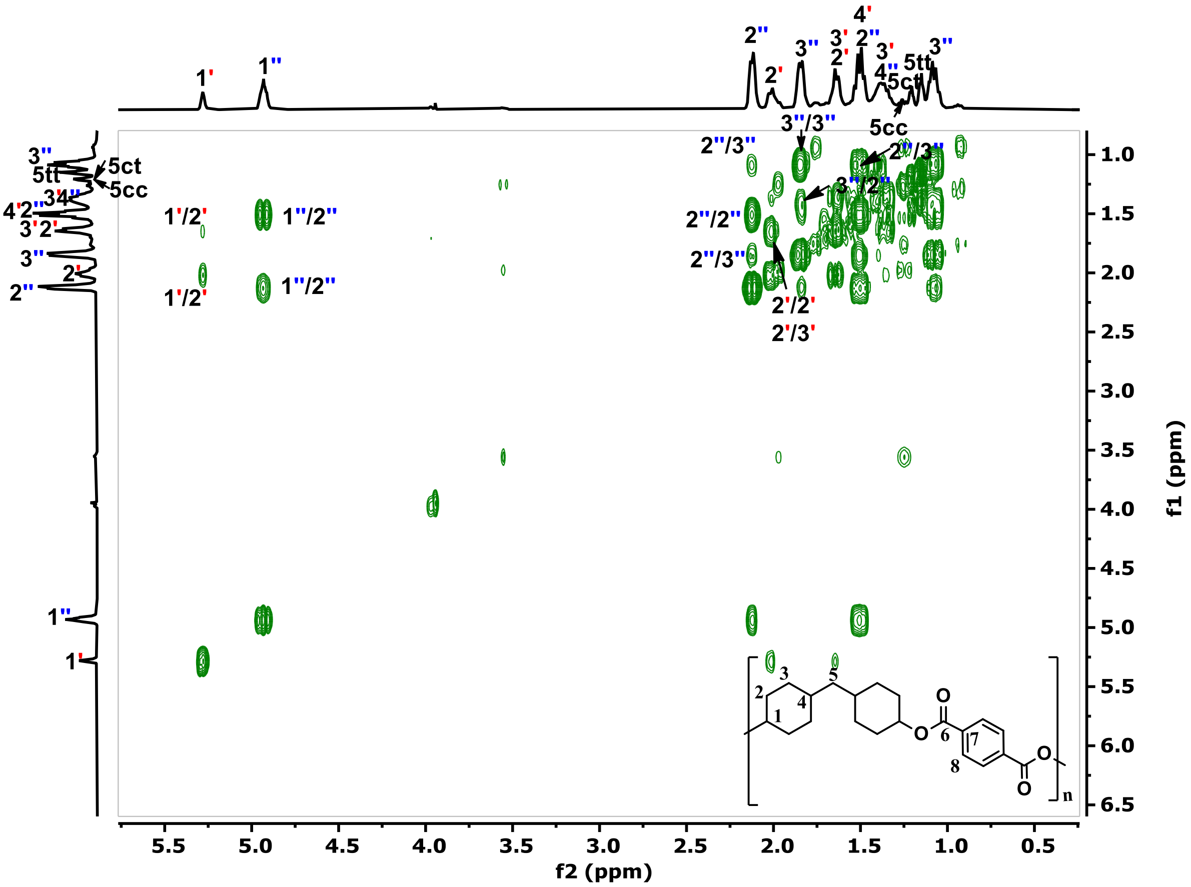

Figure S32 2D COSY spectrum of poly(MBC/TPA)

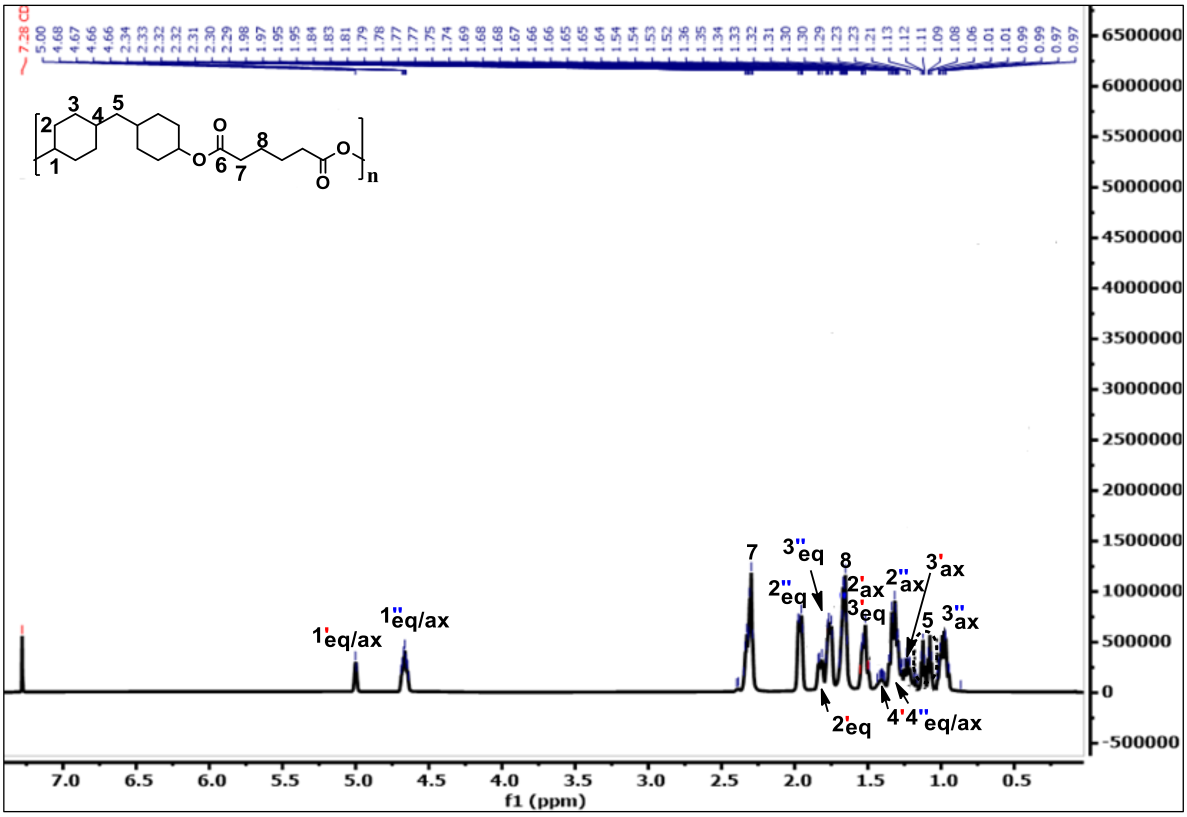

Figure S33 ^{1}H NMR spectrum of poly(MBC/AA)

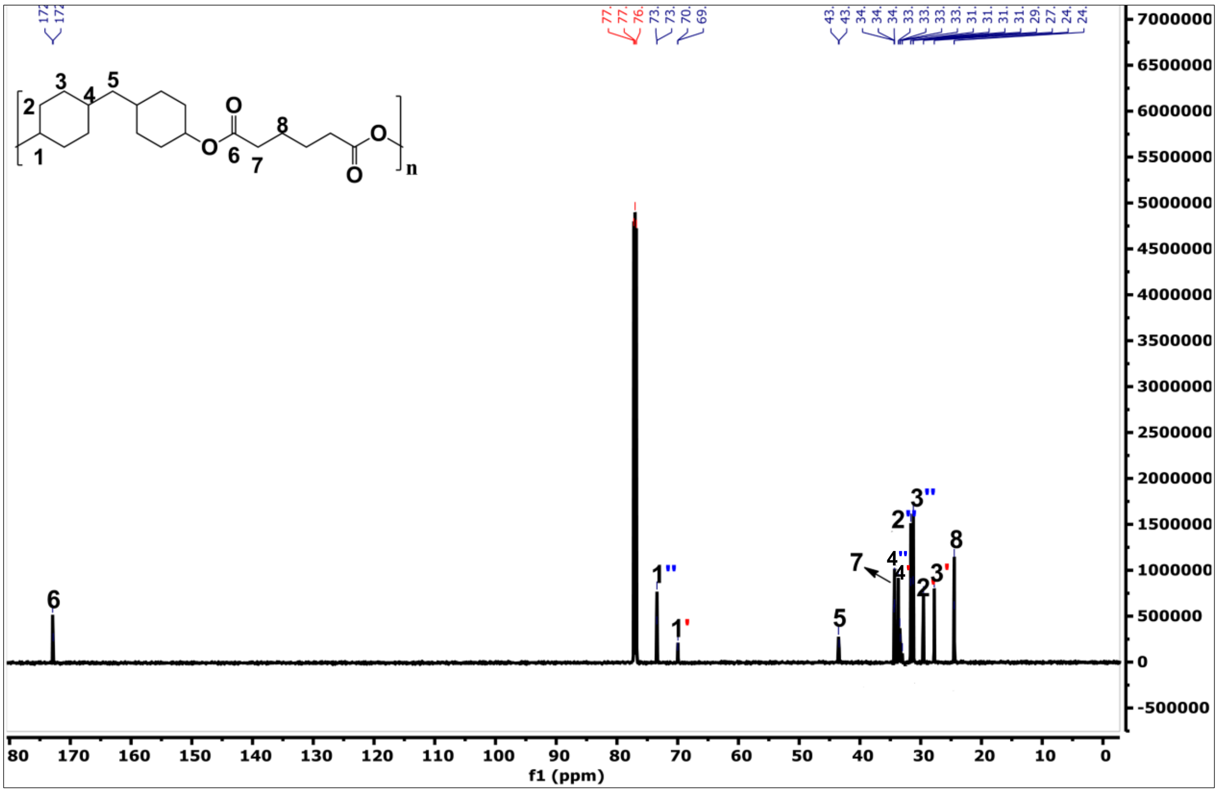

Figure S34 ^{13}C NMR spectrum of poly(MBC/AA)

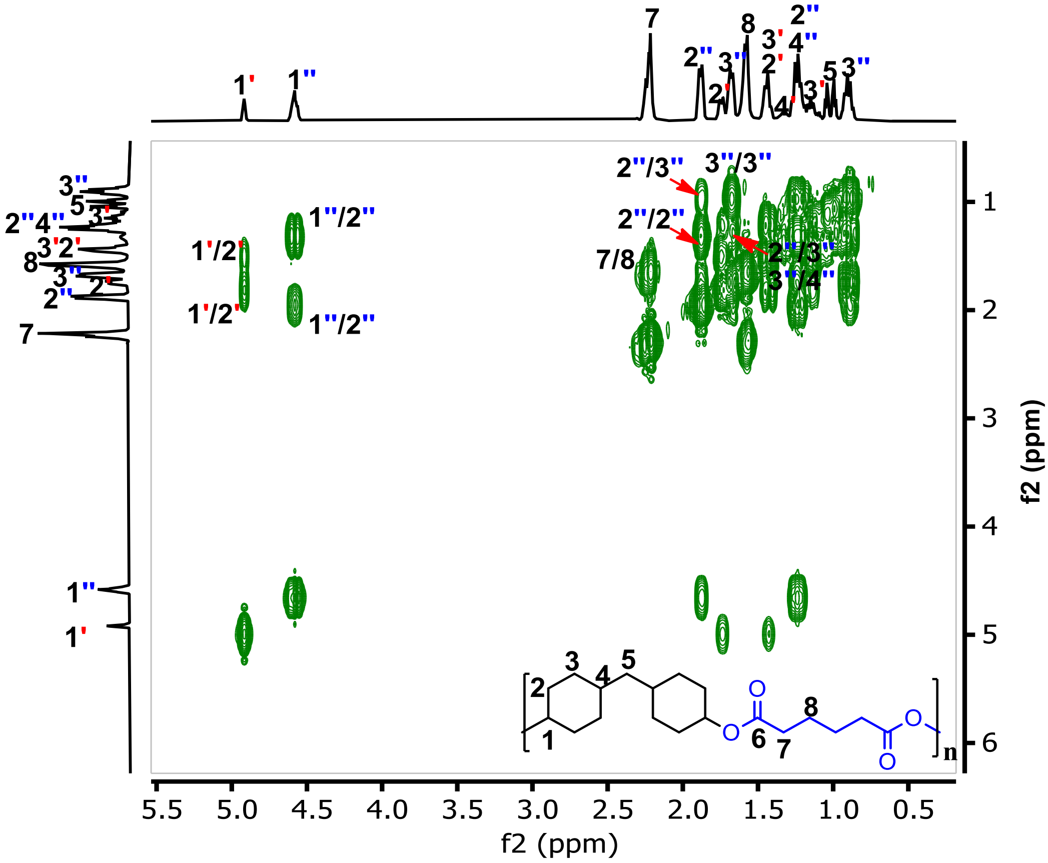

Figure S35 2D COSY spectrum of poly(MBC/AA)

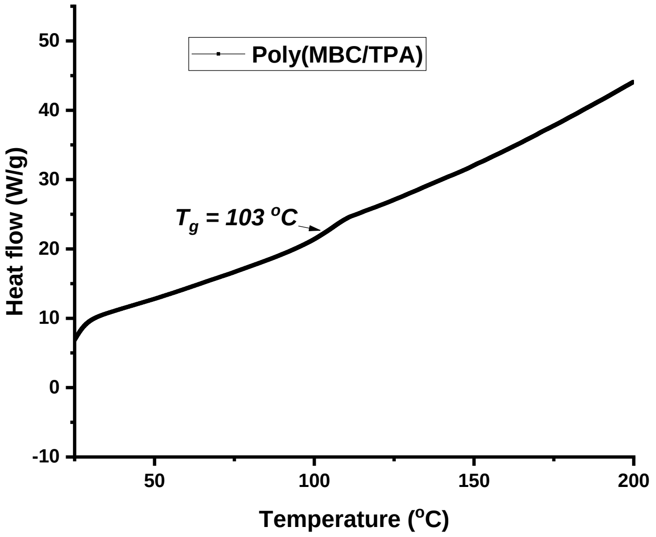

Figure S36 DSC thermogram of poly(MBC/TPA)

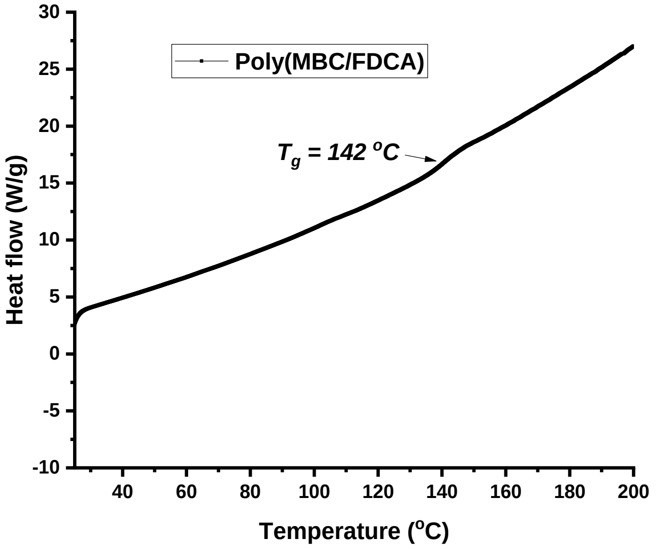

Figure S37 DSC thermogram of poly(MBC/FDCA)

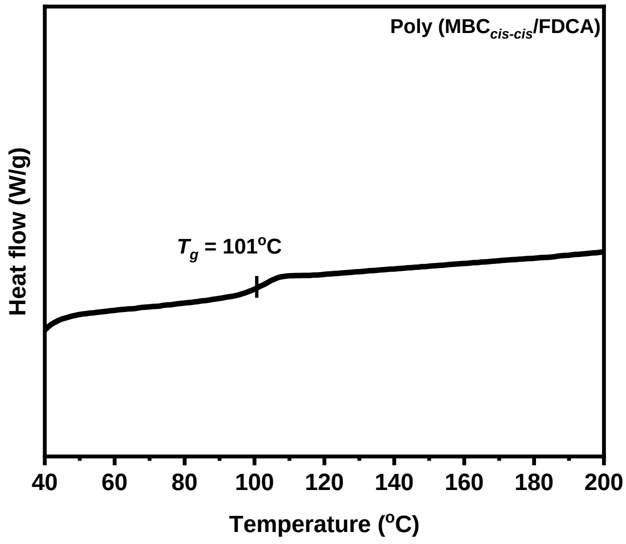

Figure S38 DSC thermogram of poly(MBC$_{\text{cis-cis}}$/FDCA)

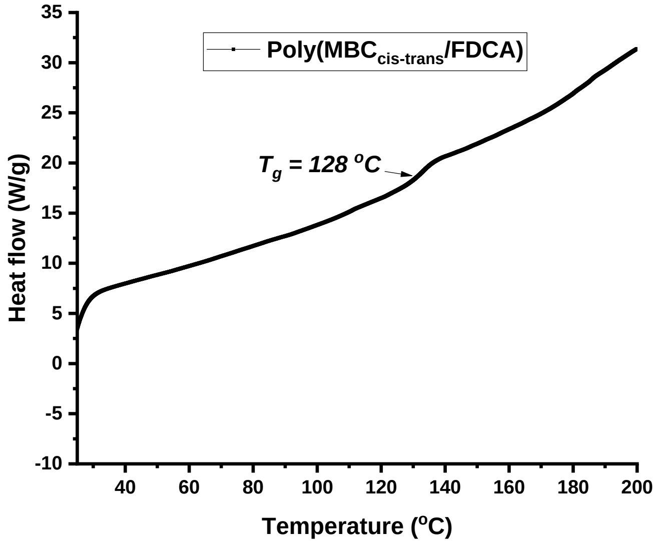

Figure S39 DSC thermogram of poly(MBC$_{\text{cis-trans}}$/FDCA)

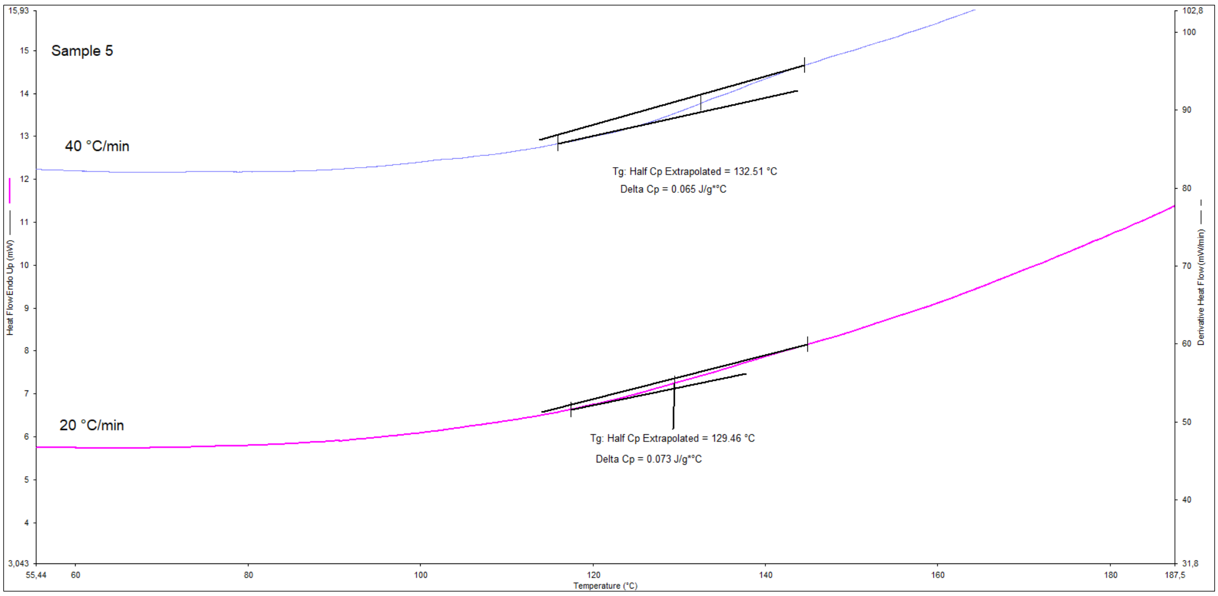

Figure S40 DSC thermograms of poly(MBC$_{trans-trans}$/FDCA) with heating rates of 20 °C min^{-1} and 40 °C min^{-1}

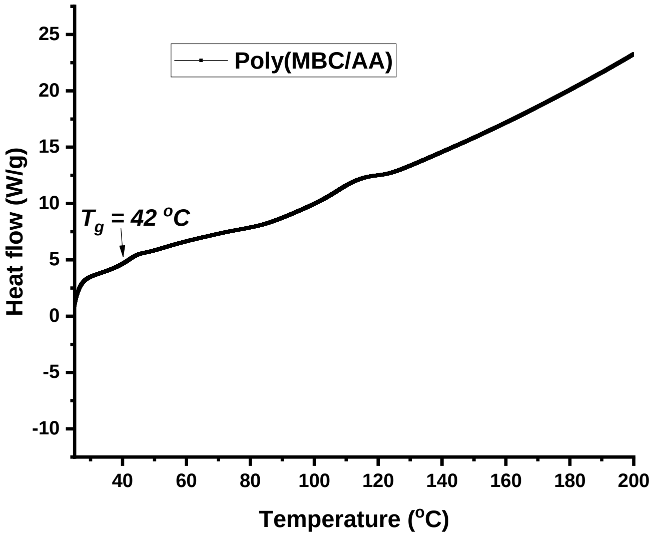

Figure S41 DSC thermogram of poly(MBC/AA)

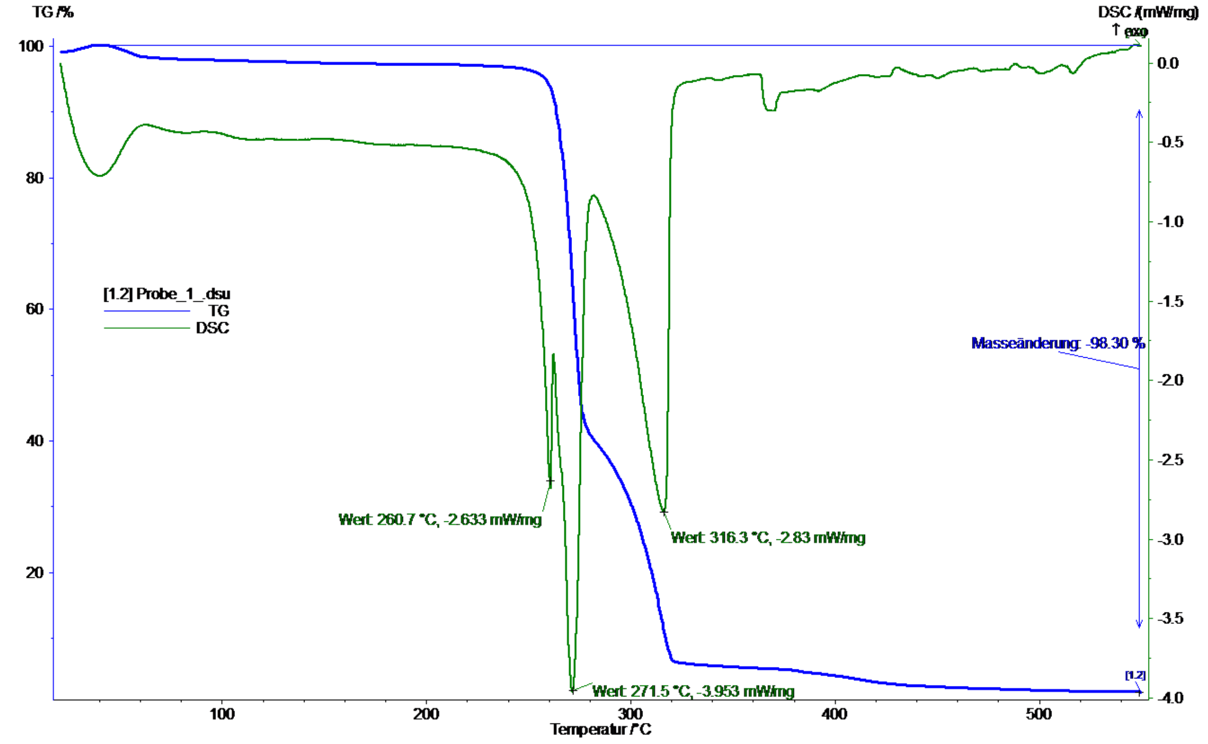

Figure S42 TGA plot of poly(MBC/TPA)

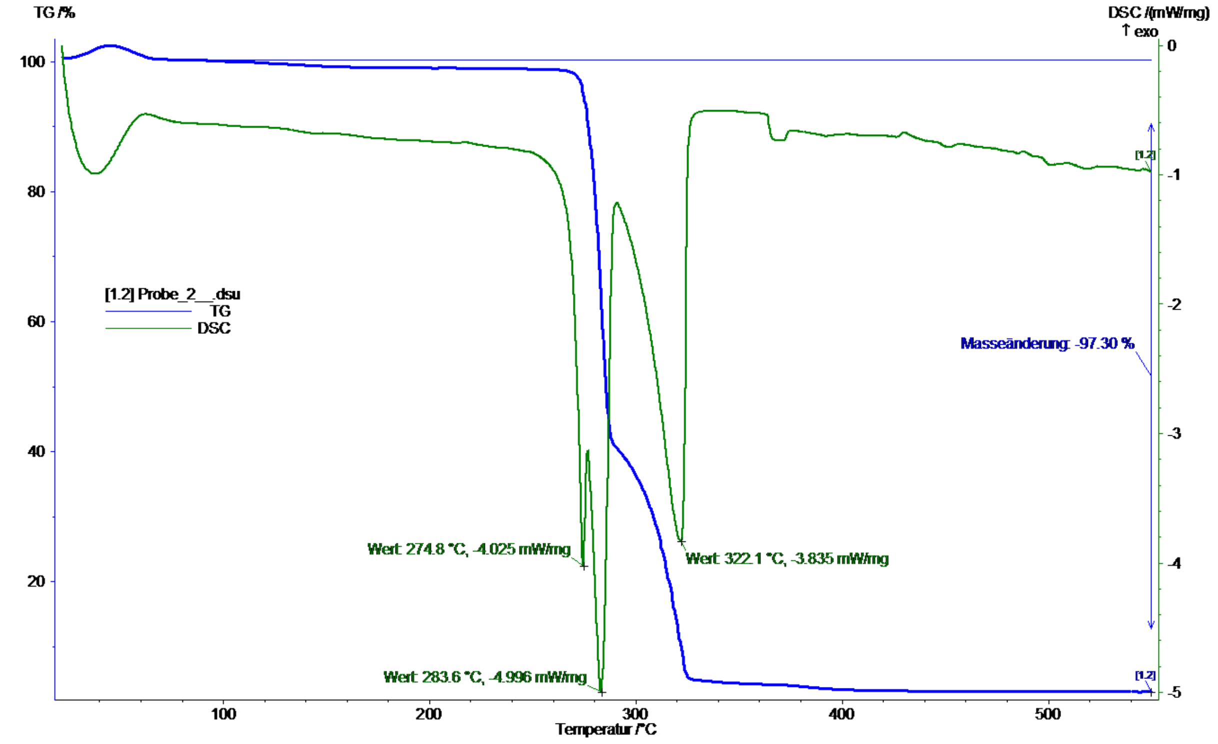

Figure S43 TGA plot of poly(MBC/FDCA)

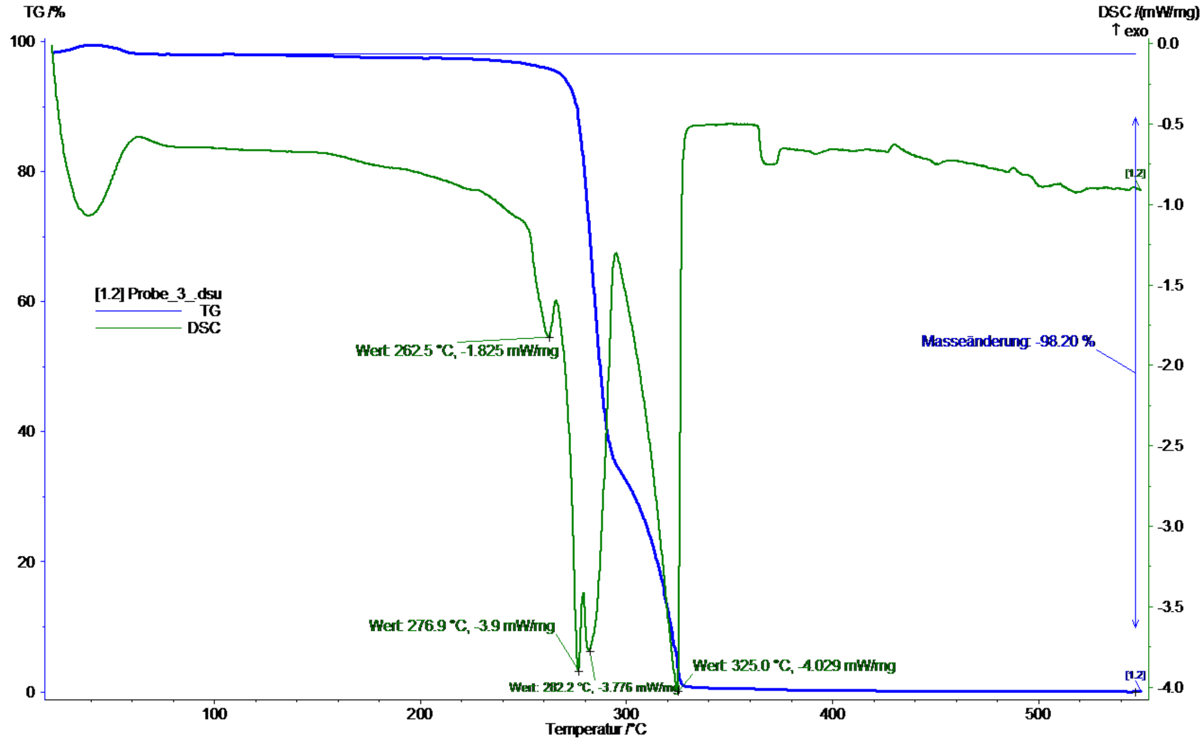

Figure S44 TGA plot of poly(MBC$_{cis\text{-}cis}$/ FDCA)

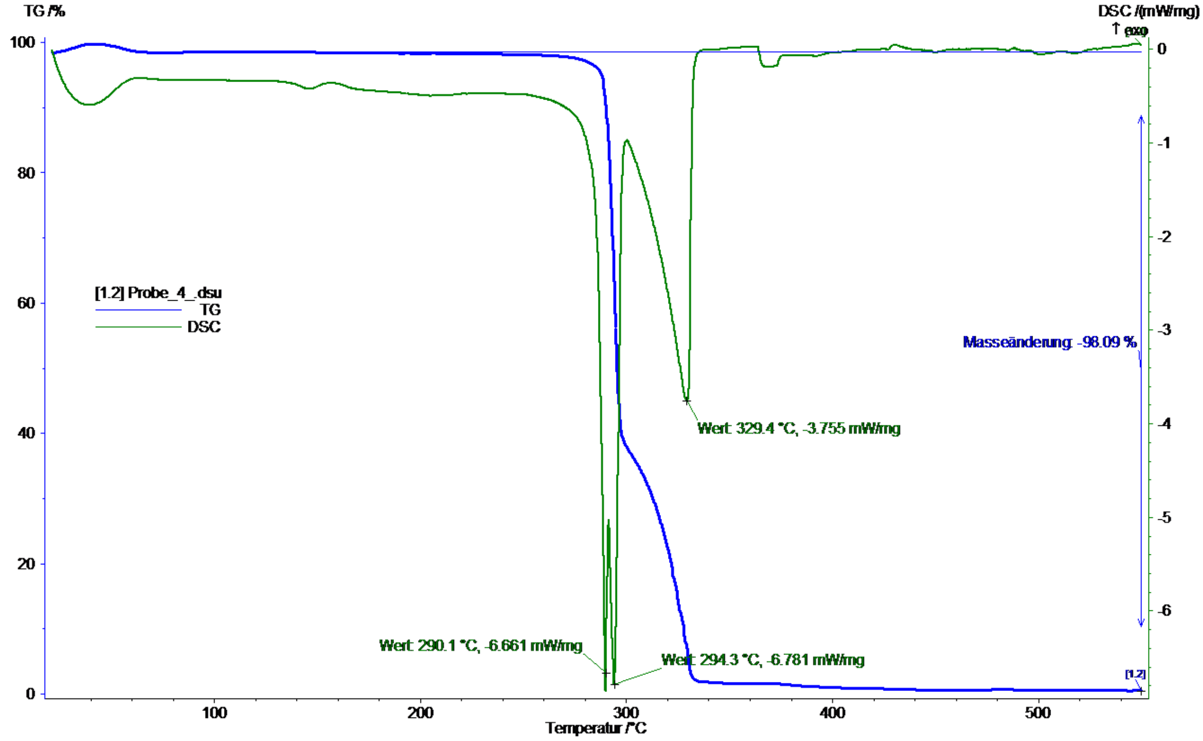

Figure S45 TGA plot of poly(MBC$_{cis\text{-}trans}$/FDCA)

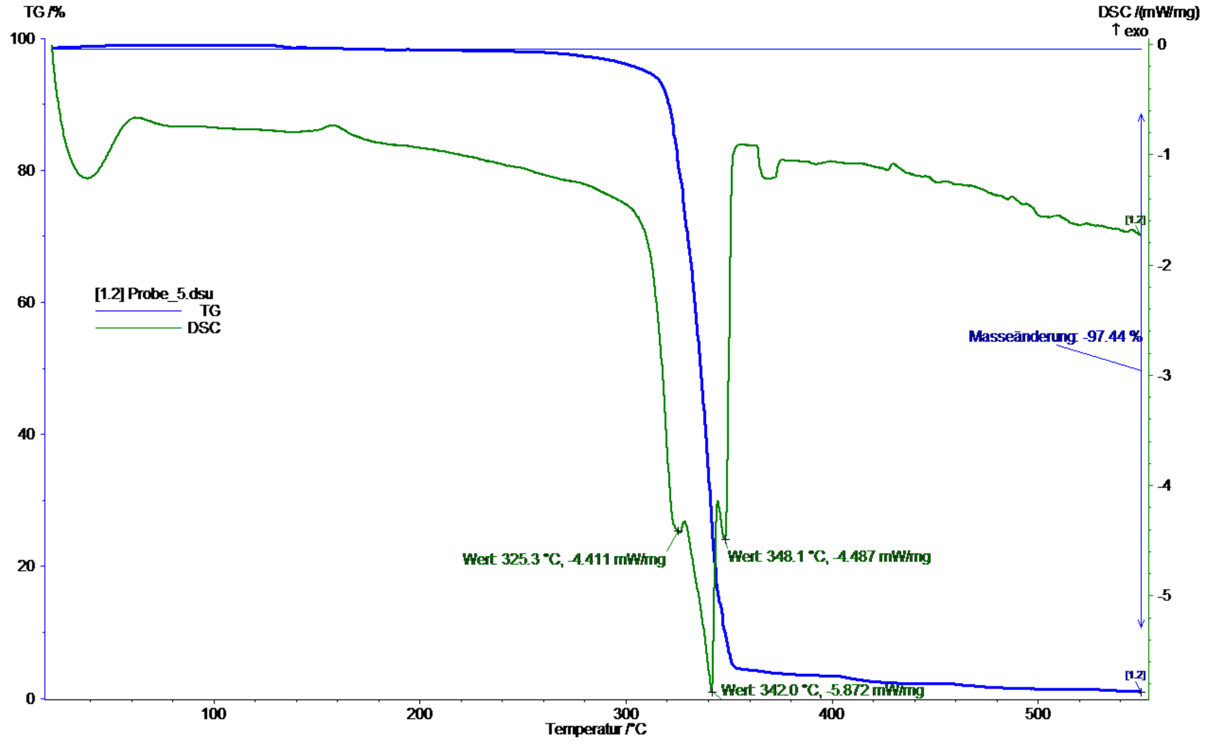

Figure S46 TGA plot of poly($MBC_{trans\text{-}trans}$/FDCA)

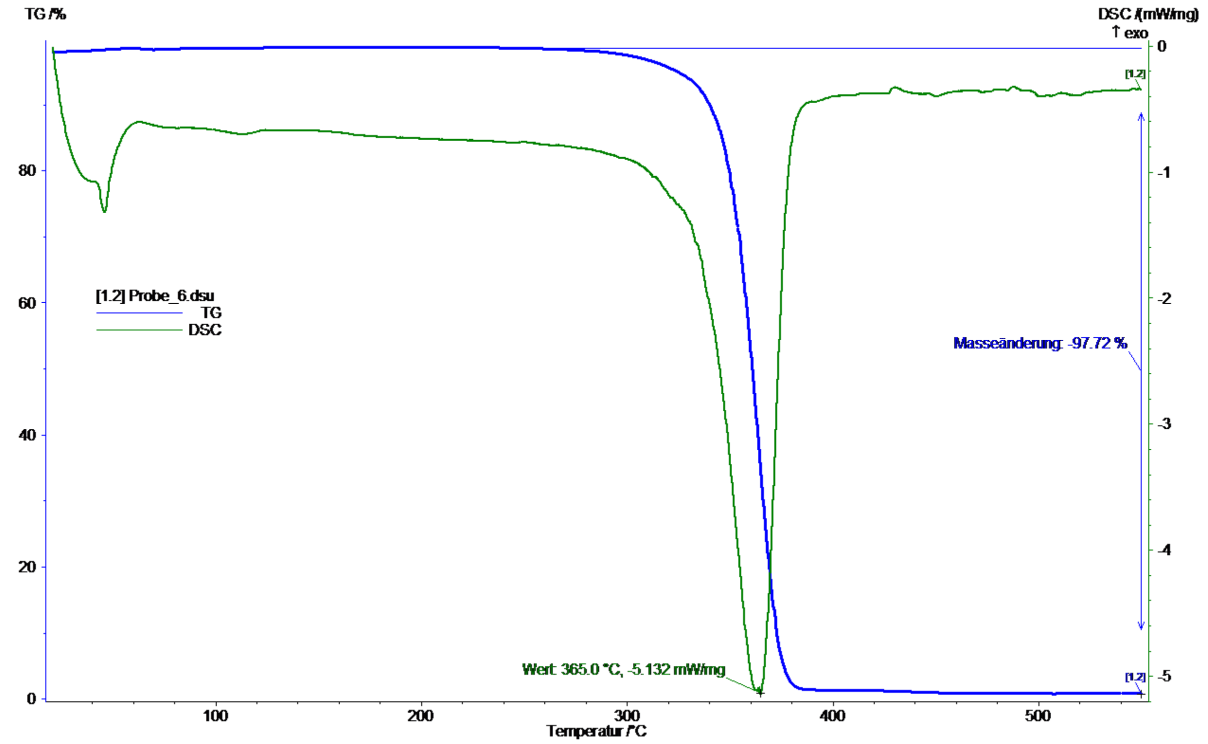

Figure S47 TGA plot of poly(MBC/AA)

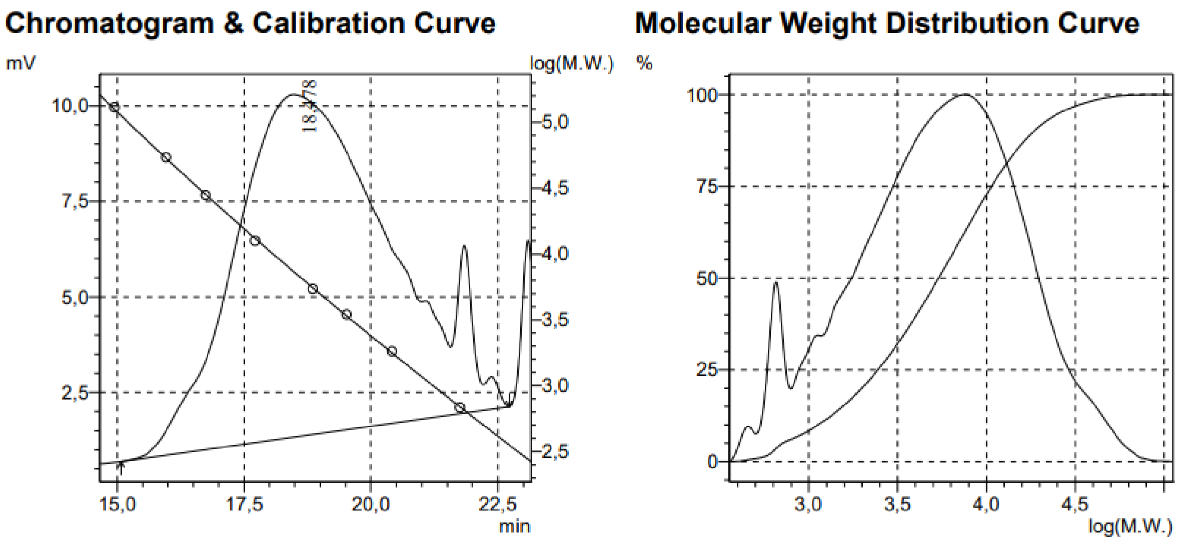

Figure S48 GPC traces of poly(MBC/TPA)

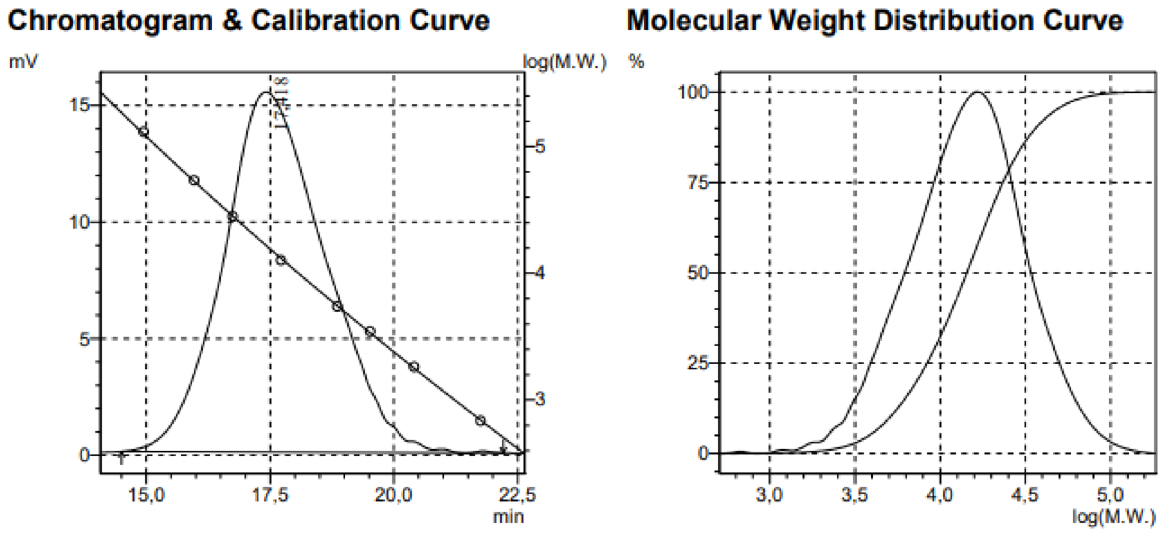

Figure S49 GPC traces of poly(MBC/FDCA)

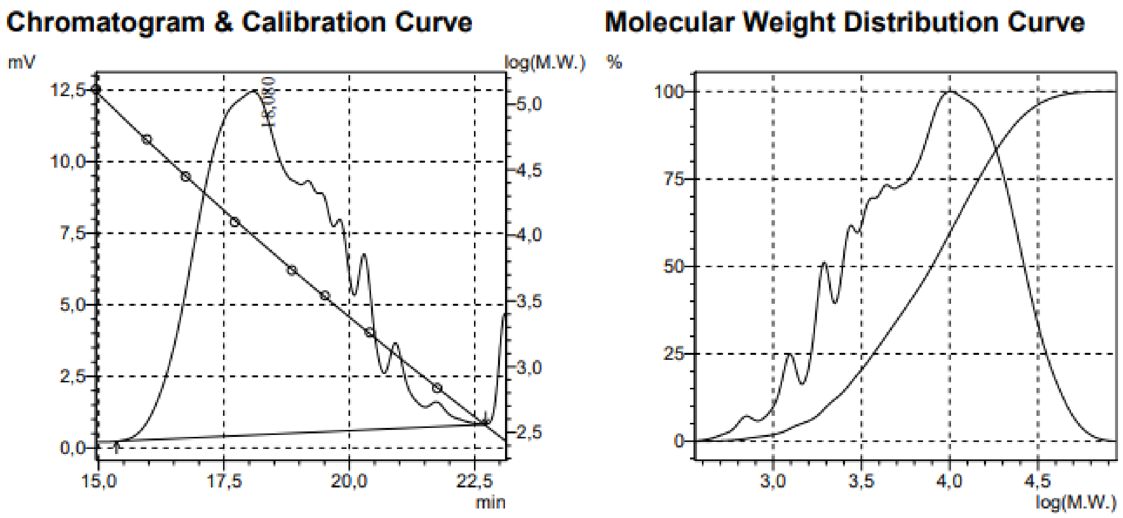

Figure S50 GPC traces of poly($MBC_{cis\text{-}cis}$/FDCA)

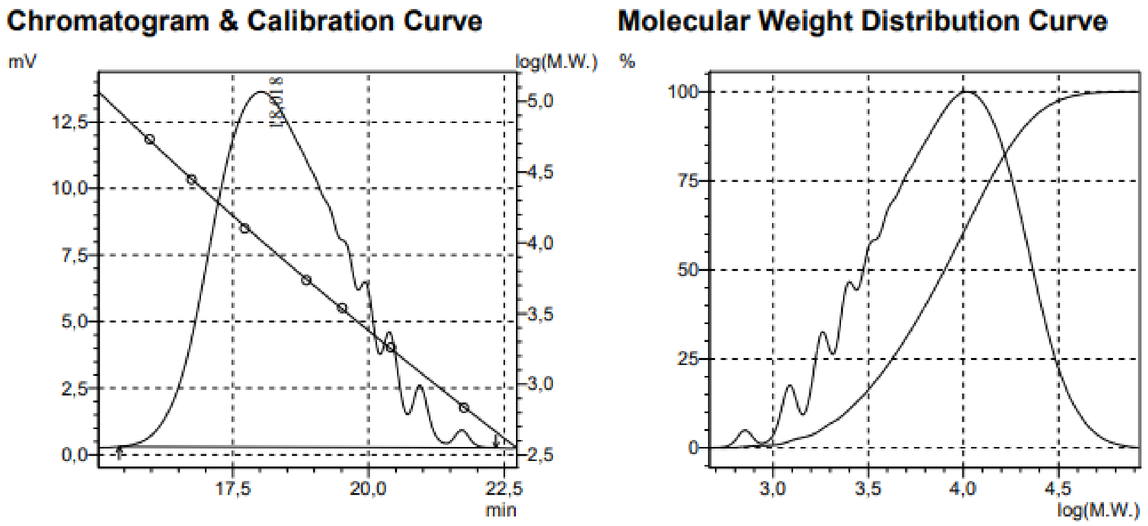

Figure S51 GPC traces of poly(MBC$_{cis\text{-}trans}$/FDCA)

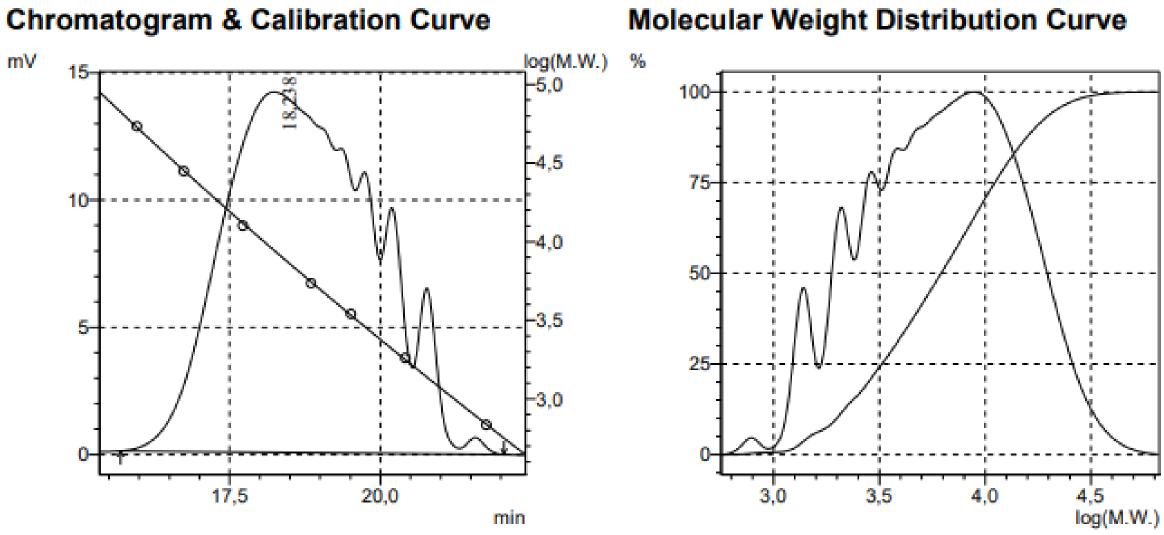

Fig. S52 GPC traces of poly(MBC$_{trans\text{-}trans}$ /TPA)

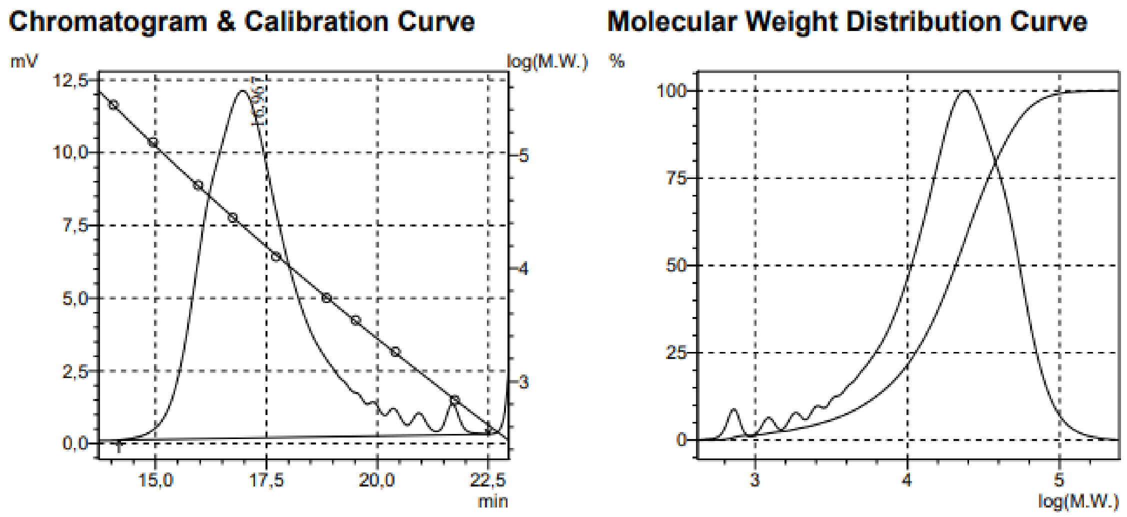

Fig. S53 GPC traces of poly(MBC/AA)

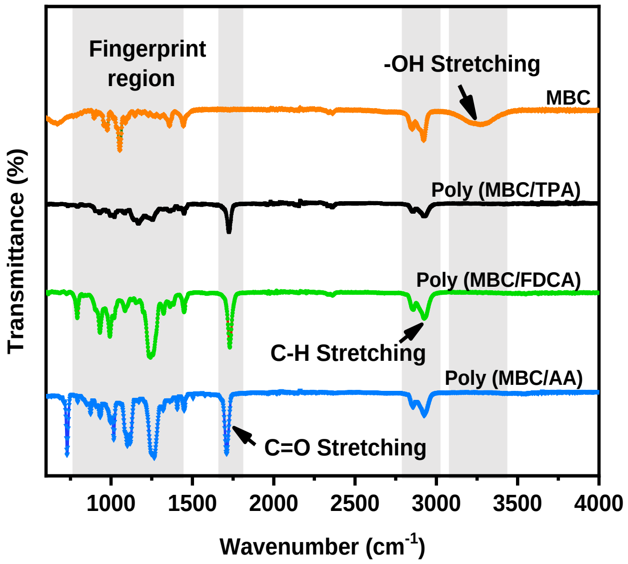

Fig. S54 FTIR spectroscopy of poly(MBC/TPA), poly(MBC/FDCA) and poly(MBC/AA)

3.1 XRD patterns of poly(MBC/TPA), poly(MBC/FDCA) and poly(MBC/AA)

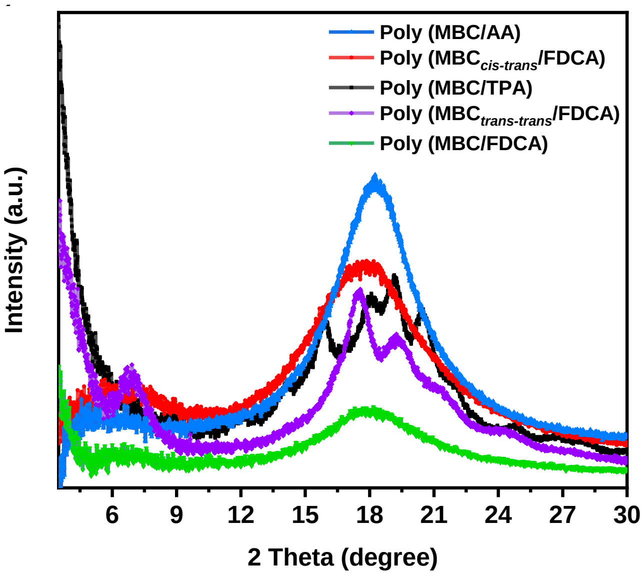

Fig. S55 XRD patterns of poly(MBC/TPA), poly(MBC/FDCA) and poly(MBC/AA)

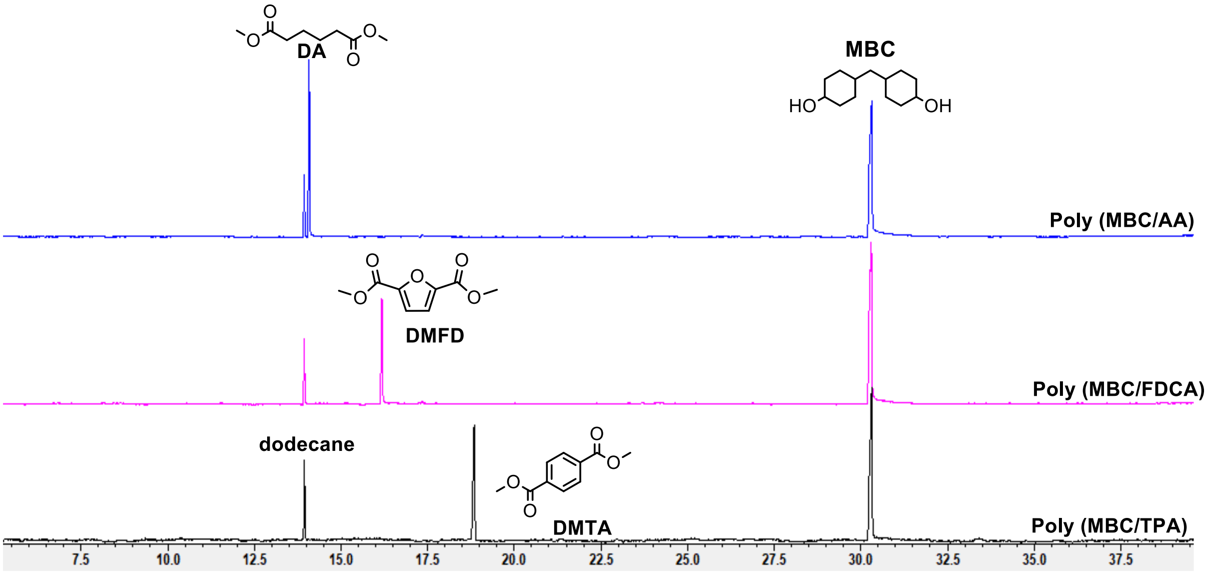

Figure S56 GC-FID traces of crude product mixtures obtained from methanolysis of the poly(MBC/TPA), poly(MBC/FDCA) and poly(MBC/AA).

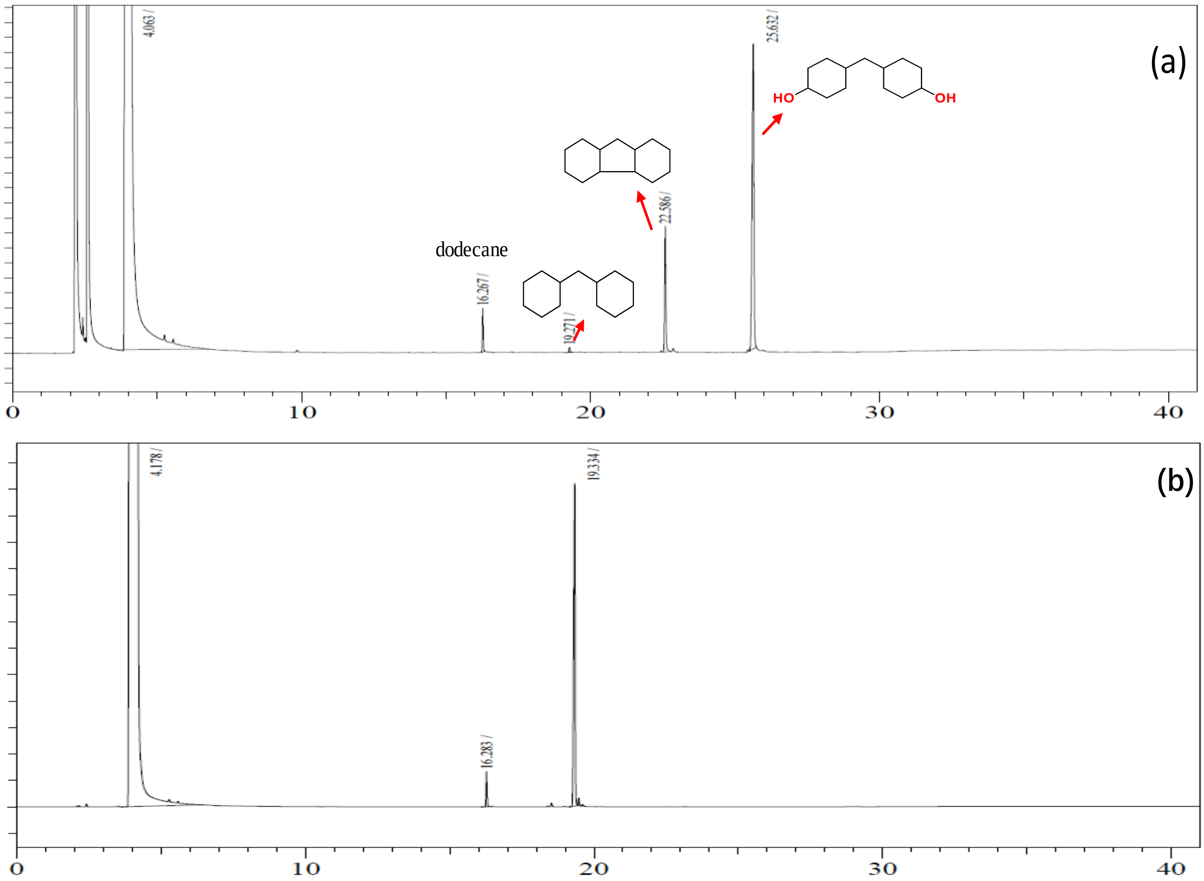

Figure S57. GC-FID traces of the crude hydrodeoxygenation mixture of MBC as obtained in the presence of Ni/HZSM-5 as the catalyst at different reaction temperatures for 4h. (a) 140 °C, (b) 180 °C.

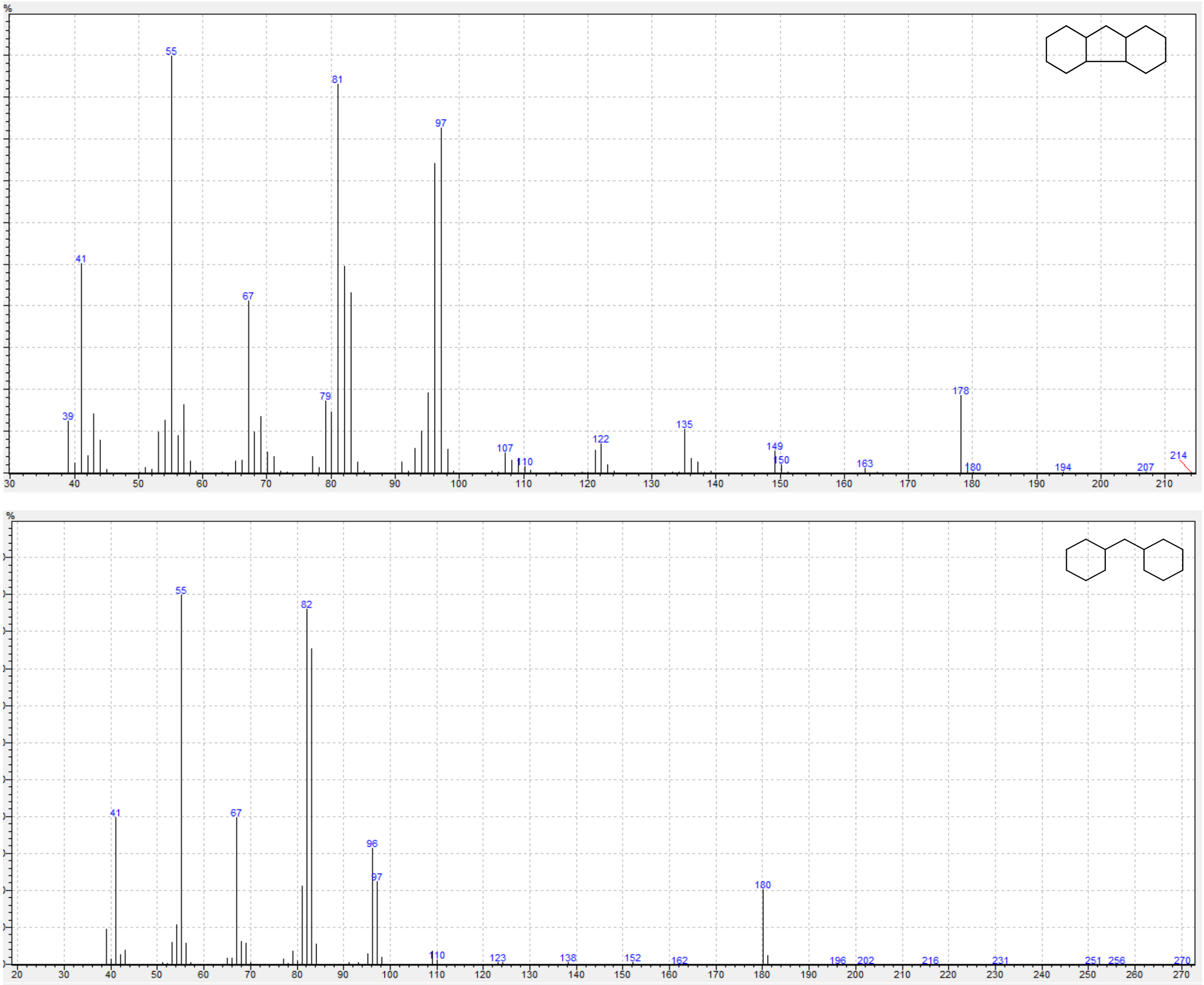

Figure S58 Mass spectra of JF-1 (up) and JF-2 (down)

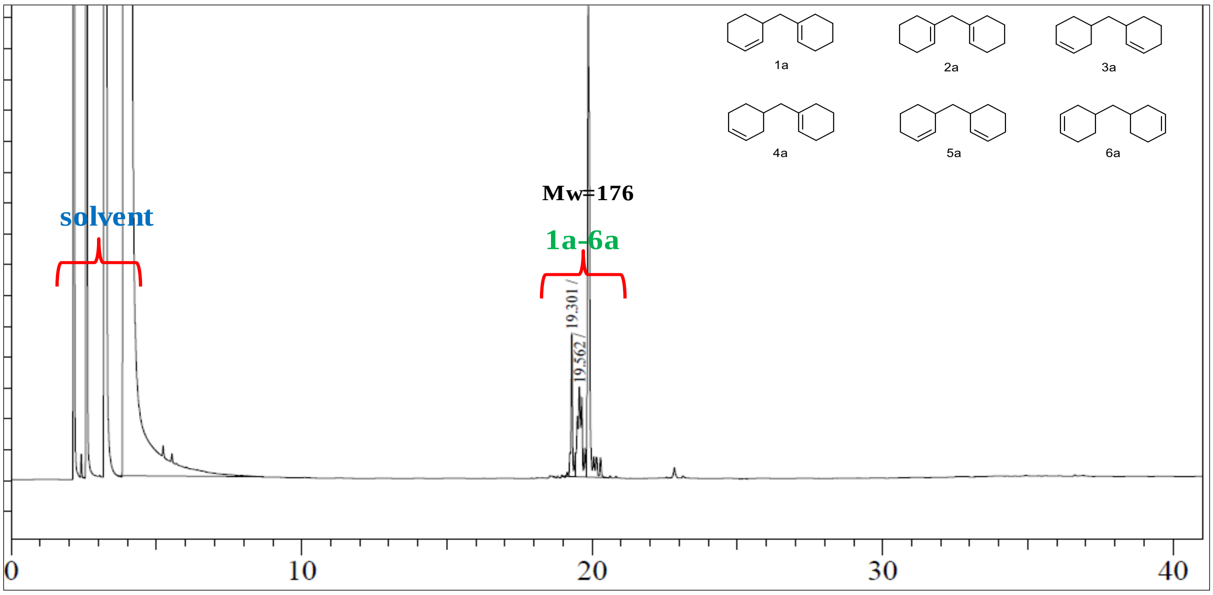

Figure S59 GC-MS trace of catalytic dehydration of MBC over HZSM-5 catalyst at 180 °C for 4 h

Supplementary References

1. Wu X. Y.; Galkin M. V.; and Barta, K.; A well-defind diamine from lignin depolymerization mixtures for constructing bio-based polybenzoxazines, *Chem. Catal.*, **2021**, *1* (7), 1360-1362. https://doi.org/10.1016/j.checat.2021.10.022
2. Gomes, M.; Gandini, A.; Silvestre, A. J.; Reis, B., Synthesis and characterization of poly (2, 5-furan dicarboxylate) s based on a variety of diols. *J. Polym. Sci. A Polym. Chem.* **2011**, *49* (17), 3759-3768. https://doi.org/10.1002/pola.24812
3. Guidotti, G.; Soccio, M.; Garcia-Gutierrez, M. C.; Ezquerra, T.; Siracusa, V.; Gutierrez-Fernandez, E.; Munari, A.; Lotti, N., Fully Biobased Superpolymers of 2,5-Furandicarboxylic Acid with Different Functional Properties: From Rigid to Flexible, High Performant Packaging Materials. *ACS Sustainable Chem. Eng.* **2020**, *8* (25), 9558-9568. https://doi.org/10.1021/acssuschemeng.0c02840
4. Wang, J. G.; Liu, X. Q.; Jia, Z.; Sun, L. Y.; Zhu, J., Highly crystalline polyesters synthesized from furandicarboxylic acid (FDCA): Potential bio-based engineering plastic. *Eur. Polym. J.* **2018**, *109*, 379-390. https://doi.org/10.1016/j.eurpolymj.2018.10.014
5. Terzopoulou, Z.; Kasmi, N.; Tsanaktsis, V.; Doulakas, N.; Bikiaris, D. N.; Achilias, D. S.; Papageorgiou, G. Z., Synthesis and Characterization of Bio-Based Polyesters: Poly(2-methyl-1,3-propylene-2,5-furanoate), Poly(isosorbide-2,5-furanoate), Poly(1,4-cyclohexanedimethylene-2,5-furanoate). *Materials* **2017**, *10* (7), 801. https://doi.org/10.3390/ma10070801
6. Curia, S.; Biundo, A.; Fischer, I.; Braunschmid, V.; Gübitz, G. M.; Stanzione III, J. F., Towards Sustainable High-Performance Thermoplastics: Synthesis, Characterization, and Enzymatic Hydrolysis of Bisguaiacol-Based Polyesters. *ChemSusChem* **2018**, *11* (15), 2529-2539 https://doi.org/10.1002/cssc.201801059
7. Gubbels, E.; Jasinska-Walc, L.; Koning, C. E., Synthesis and characterization of novel

renewable polyesters based on 2, 5-furandicarboxylic acid and 2, 3-butanediol. *J. Polym. Sci. A Polym. Chem.* **2013**, *51* (4), 890-898. https://doi.org/10.1002/pola.26446

8. Llevot, A.; Grau, E.; Carlotti, S.; Greliera, S.; Cramail, H., Renewable (semi)aromatic polyesters from symmetrical vanillin-based dimers. *Polym. Chem.* **2015**, *6* (33), 6058-6066. https://doi.org/10.1039/C5PY00824G
9. Geng, Y. T.; Wang, Z.; Hu, X. R.; Li, Y.; Zhang, Q. N.; Li, Y. J.; Wang, R. G.; Zhang, L. Q., Bio-based polyesters based on 2,5-furandicarboxylic acid as 3D-printing materials: Design, preparation and performances. *Eur. Polym. J.* **2019**, *114*, 476-484. https://doi.org/10.1016/j.eurpolymj.2018.10.041
10. Genovese, L.; Lotti, N.; Siracusa, V.; Munari, A., Poly(Neopentyl Glycol Furanoate): A Member of the Furan-Based Polyester Family with Smart Barrier Performances for Sustainable Food Packaging Applications. *Materials* **2017**, *10* (9), 1028. https://doi.org/10.3390/ma10091028
